# Supplementary material for: Reaching elimination of onchocerciasis transmission with long-term vector control and ivermectin treatment in Togo
Source: Nat Commun. 2025 Dec 19;17:779. doi: 10.1038/s41467-025-67451-8 (PMC12823694; doi:10.1038/s41467-025-67451-8)
Supplement: Supplementary file 1 — Supplementary Information [file 41467_2025_67451_MOESM1_ESM.pdf]

Reaching Elimination of Onchocerciasis Transmission with Long-term Vector Control and Ivermectin Treatment in Togo

Supplementary Material: History of onchocerciasis control in Togo, modelled infection trends in villages without recorded baseline microfilarial prevalence estimates, and probabilities of elimination of transmission

Luís-Jorge Amaral[1,2,*], Rachel N. Bronzan[3,4], Anders Seim[5], Marie-Denise Milord[3], Koffi Padjoudoum[6], Ibrahim Gado Telou[7], Sibabe Agoro[7], Michel Datagni[8], Piham Gnossike[9], Jonathan I. D. Hamley[1,10], Martin Walker[1,11] & Maria-Gloria Basáñez[1,*]

[1] MRC Centre for Global Infectious Disease Analysis, Department of Infectious Disease Epidemiology, School of Public Health, Imperial College London, London, UK

[2] Global Health Institute, University of Antwerp, Antwerp, Belgium

[3] Health & Development International, Newburyport, Massachusetts, USA

[4] Gates Foundation, Seattle, Washington, USA

[5] Health & Development International, Fjellstrand, Norway

[6] National Onchocerciasis Control Program, Kara, Togo

[7] Ministère de la Santé et de l'Hygiène Publique, Lomé, Togo

[8] Health and Development International, Lomé, Togo

[9] Neglected Tropical Diseases Coordinator, Ministère de la Santé et de l'Hygiène Publique, Lomé, Togo

[10] Department of Visceral Surgery and Medicine, and Multidisciplinary Center for Infectious Diseases, University of Bern, Switzerland

[11] Department of Pathobiology and Population Sciences, Royal Veterinary College, Hatfield, UK

Correspondence: Prof. María-Gloria Basáñez (m.basanez@imperial.ac.uk) and Luís-Jorge Amaral (luis.amaral20@imperial.ac.uk; luisjtmamaral@gmail.com), Department of Infectious Disease Epidemiology, Faculty of Medicine (White City campus), School of Public Health Building, Imperial College London, 90 Wood Lane, London W12 0BZ, UK.

Table of Contents

Supplementary Text 1. Togo heterogeneous onchocerciasis endemicity and history of control 6

1.1 Onchocerciasis pre-control endemicity in Togo 6

Supplementary Figure 1. Baseline endemicity of onchocerciasis across Togo by prefecture. 7

Supplementary Table 1. Number of villages surveyed for *Onchocerca volvulus* microfilariae per region, prefecture and endemicity level in Togo 9

1.2 History of onchocerciasis control in Togo 13

Supplementary Figure 2. Onchocerciasis control in Togo 14

Supplementary Figure 3. Temporal trends of crude microfilarial prevalence in villages located in Special Intervention Zone (SIZ) and non-SIZ areas from 1975 to 2017 16

Supplementary Figure 4. Box-and-whisker plots of crude microfilarial prevalence in non-SIZ (No) and SIZ (Yes) villages in Togo across different intervention periods 17

Supplementary Table 2. History of onchocerciasis control between 1975 and 2018 per region and prefecture in Togo 18

Supplementary Table 2. Continued 19

Supplementary Table 2. Continued 20

Supplementary Table 2. Continued 21

Supplementary Table 2. Continued 22

Supplementary Table 2. Continued 23

Supplementary Table 2. Continued 24

Supplementary Table 2. Continued 25

Supplementary Text 2. Data sources 26

Supplementary Figure 5. Geographical distribution of villages for onchocerciasis monitoring across regions in Togo 26

Relationship between crude and age- and sex-standardised microfilarial prevalence 27

Supplementary Figure 6. Linear relationship between crude microfilarial prevalence and age- and sex-standardised microfilarial prevalence 27

Supplementary Text 3. Estimation of Annual Biting Rates (ABRs) 28

Supplementary Table 3. Modelled annual biting rate (ABR) for each pre-control endemicity (microfilarial baseline prevalence) level 28

Supplementary Table 4. Annual biting rates measured at vector capture points prior to vector control, and baseline microfilarial prevalence (BMP) in surveyed villages 29

Supplementary Table 4. Continued 30

Supplementary Table 4. Continued 31

Supplementary Text 4. Minimal, reference and enhanced intervention scenarios 32

Reported ivermectin treatment coverage of total population (%) per region and prefecture from 1991 to 2018 in Togo 33

Supplementary Table 5. Reported coverage (% of total population) of ivermectin MDA for 1991-2018 in Savanes 33

Supplementary Table 6. Reported coverage (% of total population) of ivermectin MDA for 1991-2018 in Kara 34

Supplementary Table 7. Reported coverage (% of total population) of ivermectin MDA for 1991-2018 in Centrale 35

Supplemantary Table 8. Reported coverage (% of total population) of ivermectin MDA for 1991-2018 in Plateaux 36
Supplementary Table 9. Reported coverage (% of total population) of ivermectin MDA for 1991-2018 in Maritime 37

Supplementary Text 5. Proportion of the population surveyed over time 38

Supplementary Figure 7. Box-and-whisker plots of the proportion of the population surveyed per village according to survey years 38

Villages with recorded baseline microfilarial prevalence estimates in the OCP database 39

Supplementary Table 10. Villages with baseline microfilarial prevalence (BMP) estimates of *Onchocerca volvulus* by region, endemicity level and special intervention zone (SIZ) status in Togo 39
Supplementary Table 10. Continued 40

Supplementary Text 6. Modelled infection trends by region and Special Intervention Zone (SIZ) status for villages without recorded baseline microfilarial prevalence estimates of *Onchocerca volvulus* 41

Supplementary Table 11. Best-fit intervention scenarios, indicated by the smallest (median) mean square error (MSE) values across 100 model repeats for villages with and without recorded baseline microfilarial prevalence (BMP), by region, Special Intervention Zone (SIZ) status and endemicity 42
Supplementary Table 11. Continued 43
Supplementary Figure 8. *Onchocerca volvulus* microfilarial prevalence trends simulated using EPIONCHO-IBM (until 2030) and survey data for villages without recorded baseline microfilarial prevalence (BMP) estimates for Savanes Region within the Special Intervention Zone 44
Supplementary Figure 9. *Onchocerca volvulus* microfilarial prevalence trends simulated using EPIONCHO-IBM (until 2030) and survey data for villages without recorded baseline microfilarial prevalence (BMP) estimates for Savanes Region outside the Special Intervention Zone 45
Supplementary Figure 10. *Onchocerca volvulus* microfilarial prevalence trends simulated using EPIONCHO-IBM (until 2030) and survey data for villages without recorded baseline microfilarial prevalence (BMP) estimates for Kara Region within the Special Intervention Zone 46
Supplementary Figure 11. *Onchocerca volvulus* microfilarial prevalence trends simulated using EPIONCHO-IBM (until 2030) and survey data for villages without recorded baseline microfilarial prevalence (BMP) estimates for Centrale Region within the Special Intervention Zone 48
Supplementary Figure 12. *Onchocerca volvulus* microfilarial prevalence trends simulated using EPIONCHO-IBM (until 2030) and survey data for villages without recorded baseline microfilarial prevalence (BMP) estimates for Centrale Region outside the Special Intervention Zone 49
Supplementary Figure 13. *Onchocerca volvulus* microfilarial prevalence trends simulated using EPIONCHO-IBM (until 2030) and survey data for villages without recorded baseline microfilarial prevalence (BMP) estimates for Plateaux Region, not included in the Special Intervention Zone 51
Supplementary Figure 14. *Onchocerca volvulus* microfilarial prevalence trends simulated using EPIONCHO-IBM (until 2030) and survey data for villages without recorded baseline microfilarial prevalence (BMP) estimates for Maritime Region, not included in the Special Intervention Zone 53

Projected probabilities of elimination of onchocerciasis transmission 54

Supplementary Table 12. Probability of elimination of onchocerciasis transmission (EOT) when simulating that ivermectin mass drug administration (MDA) stops in 2024, 2027 or 2030 per modelled (minimal, reference and enhanced) intervention scenarios in Savanes 54
Supplementary Table 12. Continued 55
Supplementary Table 13. Probability of elimination of onchocerciasis transmission (EOT) when simulating that ivermectin mass drug administration (MDA) stops in 2024, 2027 or 2030 per modelled (minimal, reference and enhanced) intervention scenarios in Kara 56

Supplementary Table 14. Probability of elimination of onchocerciasis transmission (EOT) when simulating that ivermectin mass drug administration (MDA) stops in 2024, 2027 or 2030 per modelled (minimal, reference and enhanced) intervention scenarios in Centrale 57
Supplementary Table 15. Probability of elimination of onchocerciasis transmission (EOT) when simulating that ivermectin mass drug administration (MDA) stops in 2024, 2027 or 2030 per modelled (minimal, reference and enhanced) intervention scenario in Plateaux 58
Supplementary Table 16. Probability of elimination of onchocerciasis transmission (EOT) when simulating that ivermectin mass drug administration (MDA) stops in 2014 or 2020 per modelled (minimal, reference and enhanced) intervention scenario in Maritime 59
Supplementary Table 17. Probability of elimination of onchocerciasis transmission (EOT) when simulating that ivermectin mass drug administration (MDA) stops in 2024, 2027 or 2030 per modelled (minimal, reference and enhanced) intervention scenario in Maritime 60

Supplementary Text 7. Villages projected not to reach elimination of onchocerciasis transmission (EOT) if ivermectin MDA stops in 2027, per region and special intervention zone (SIZ) status 61

7.1. Villages with recorded baseline microfilarial prevalence estimates (with BMP) 61
Supplementary Table 18. Villages in Savanes not included in the special intervention zone (non-SIZ) 61
Supplementary Table 19. Villages in Kara included in the special intervention zone (SIZ) 62
Supplementary Table 20. Villages in Centrale included in the special intervention zone (SIZ) 62
Supplementary Table 21. Villages in Centrale not included in the special intervention zone (non-SIZ) 63
Supplementary Table 22. Villages in Plateaux not included in the special intervention zone (non-SIZ) 64
Supplementary Table 22. Continued 65
Supplementary Table 22. Continued 66
Supplementary Table 23. Villages in Maritime not included in the special intervention zone (non-SIZ) 67

7.2. Villages without recorded baseline microfilarial prevalence (without BMP) 68
Supplementary Table 24. Villages in Savanes included in the special intervention zone (SIZ) 68
Supplementary Table 24. Continued 69
Supplementary Table 25. Villages in Kara included in the special intervention zone (SIZ) 70
Supplementary Table 25. Continued 71
Supplementary Table 26. Villages in Centrale included in the special intervention zone (SIZ) 72
Supplementary Table 27. Villages in Centrale not included in the special intervention zone (non-SIZ) 73
Supplementary Table 28. Villages in Plateaux not included in the special intervention zone (non-SIZ) 74
Supplementary Table 28. Continued 75
Supplementary Table 28. Continued 76
Supplementary Table 29. Villages in Maritime not included in the special intervention zone (non-SIZ) 77

Supplementary Text 8. Calculation of prefecture-level likelihood of reaching elimination of onchocerciasis transmission 78

Supplementary Table 30. Assigned midpoint values for village-level EOT probability ranges 78
Supplementary Table 31. Definitions of prefecture-level EOT likelihood categories 79
Supplementary Table 32. Prefecture-level likelihood of reaching EOT when simulating that ivermectin MDA stops in 2024 80
Supplementary Table 32. Continued 81
Supplementary Table 32. Continued 82
Supplementary Table 33. Prefecture-level likelihood of reaching EOT when simulating that ivermectin MDA stops in 2027 83
Supplementary Table 33. Continued 84
Supplementary Table 33. Continued 85
Supplementary Table 34. Prefecture-level likelihood of reaching EOT when simulating that ivermectin MDA stops in 2030 86
Supplementary Table 34. Continued 87

Supplementary Table 34. Continued 88
Supplementary Table 35. Prefecture-level EoT likelihood category, current interventions and recommendations 89
Supplementary Table 35. Continued 90
Supplementary Table 35. Continued 91

Supplementary Text 9. Modelling for policy: PRIME-NTD **92**

Supplementary Table 36. Policy-Relevant Items for Reporting Models in Epidemiology of Neglected Tropical Diseases (PRIME-NTD) summary table 92

Supplementary References **94**

Supplementary Text 1. Togo heterogeneous onchocerciasis endemicity and history of control

1.1 Onchocerciasis pre-control endemicity in Togo

Supplementary Fig. 1 illustrates baseline endemicity across Togo, according to different data sources and methodological approaches. The maps for Togo, and its regions and prefectures were drawn using the R package *geodata* version 0.6-2 (https://github.com/rspatial/geodata). Figure 1a shows endemicity based on initial (1970-76) surveys by the Onchocerciasis Control Programme in West Africa (OCP) at the prefecture level (village-specific data not available) [1,2]. Fig. 1b presents endemicity for villages with recorded baseline microfilarial prevalence (BMP) (Supplementary Text 2 describes data sources). Figure 1c integrates endemicity for villages with and without recorded BMP. For the latter, putative BMP was inferred from subsequent surveys and EPIONCHO-IBM simulations (Supplementary Fig. 8-14). Figure 1d depicts a synthesised map of endemicity across Togo, combining information from Fig. 1a and 1c. In Fig. 1b-1c, endemicity levels are categorised based on BMP as: non-endemic (0%); hypoendemic (>0% but <40%); mesoendemic (≥40% but <60%); hyperendemic (≥60% but <80%); holoendemic (≥80%). In Fig. 1a and 1d, non-endemic and hypoendemic categories have been combined (<40%).

Supplementary Table 1 summarises the data used in this work for 400 onchocerciasis-endemic villages organised by Togo regions, prefectures and endemicity levels. The few hypoendemic villages with recorded BMP are likely not to be representative of the true number of hypoendemic villages in Togo. During the OCP, the goal was the elimination of onchocercal blindness as a public health problem (EPHP) and, therefore, most survey efforts focused on highly-endemic villages (those closest to vector breeding sites and with high blindness prevalence) [1]. Onchocerciasis is highly focal and most prevalent in rural populations living in close proximity to vector breeding sites [3]. In Togo, the onchocerciasis vectors belong to the *Simulium damnosum sensu lato* (s.l.) species complex [4].

Savanes and Kara regions have fewer villages with recorded BMP (21%) than Centrale, Plateaux and Maritime (44%; chi-square p-value<0.001). Vector control (VC) started earlier in the former two regions [5]. The region with the largest rural population is Plateaux, whereas Savanes is the most rural region. Maritime is the most populated region, with the majority of

its population residing in urban areas, and hence the least onchocerciasis-endemic region (see Supplementary Text 2 and Supplementary Fig. 5 for data sources) [6].

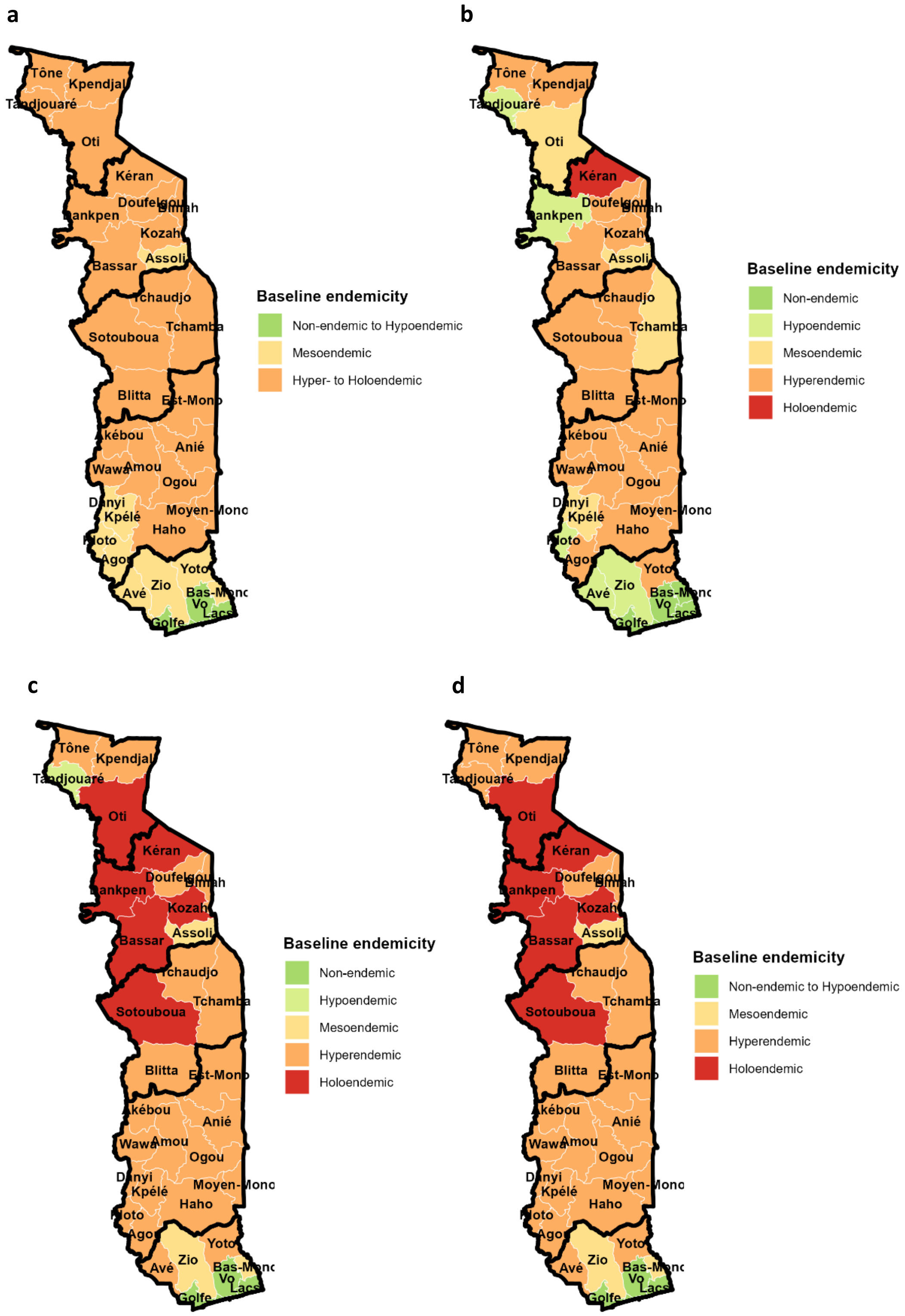

Supplementary Figure 1. Baseline endemicity of onchocerciasis across Togo by prefecture. (a) Endemicity based on initial Onchocerciasis Control Programme in West Africa (OCP) surveys (1970-76) [1,2]. (b) Endemicity based on villages with recorded baseline microfilarial prevalence (BMP, data sources described in Supplementary Text 2). (c) Endemicity based on both villages with and without recorded BMP. (d) Map

synthesising data from (a) and (c). In 1b-1c, endemicity levels are categorised based on BMP as: non-endemic (0%); hypoendemic (>0% but <40%); mesoendemic (≥40% but <60%); hyperendemic (≥60% but <80%); holoendemic (≥80%). In 1a and 1d, non-endemic and hypoendemic categories have been combined (<40%). Black thick borders indicate regions: from North to South: Savanes, Kara, Central, Plateaux and Maritime. White thin borders denote prefectures up to 2012 (see Supplementary Table 1).

Supplementary Table 1. Number of villages surveyed for *Onchocerca volvulus* microfilariae per region, prefecture and endemicity level in Togo

**Region** Prefecture[a]	**Total No. of villages surveyed**	**Endemicity level according to baseline microfilarial prevalence (BMP)**				**Without recorded BMP (%)**	**Proportion of overall population** (%)	**Proportion of rural population**[b] (%)	**Observations** (Modelled prevalence trends followed by villages without BMP)
		**Hypoendemic**	**Mesoendemic**	**Hyperendemic**	**Holoendemic**				
**Savanes**	49	4	5	3	–	37	13.4	18.4	
Kpendjal, including Kpendjal-Ouest	9	–	1	1	–	7	2.5	3.9	Hyperendemic
Oti, including Oti-Sud	29	1	2	–	–	26	3.1	4.3	Hypo- to Holoendemic
Tandjoaré or Tandjouaré	5	3[c]	–	–	–	2	1.9	3.0	–
Tône, including Cinkassé	6	–	1 + 1[c]	1 + 1[c]	–	2	4.6	5.9	–
**Kara**	88	2	4	7	2	73	12.4	15.2	
Assoli	3	1	1	–	–	1	0.8	0.9	Hypoendemic
Bassar	17	–	1	2	–	14	1.9	2.5	Hypo- to Holoendemic
Binah/Bimah	4	–	2[c]	1[c]	–	1	1.1	1.7	–
Dankpen	19	1	–	–	–	18	2.1	3.1	Hypo- to Holoendemic
Doufelgou	5	–	–	2	–	3	1.3	1.5	Meso- to Hyperendemic
Kéran	13	–	–	–	2	11	1.5	2.1	Hyper- to Holoendemic

Supplementary Table 1. Continued

**Region**<br>Prefecture[a]	**Total No. of villages surveyed**	**Endemicity level according to baseline microfilarial prevalence (BMP)**				**Without recorded BMP (%)**	**Proportion of overall population** (%)	**Proportion of rural population**[b] (%)	**Observations** (Modelled prevalence trends followed by villages without BMP)
		**Hypoendemic**	**Mesoendemic**	**Hyperendemic**	**Holoendemic**				
Kozah	27	–	–	2	–	25	3.7	3.4	Hypo- to Holoendemic
**Centrale**	86	13	26	7	–	40	10.0	12.1	
Blitta	28	7	5	5	–	11	2.2	3.3	Hypo- to Hyperendemic
Sotouboua, including Mô	34	3	10	–	–	21	2.0	2.5	Hypo- to Holoendemic
Tchamba	16	2	8	–	–	6	2.1	2.8	Hypo- to Hyperendemic
Tchaoudjo/Tchaudjo	8	1	3	2	–	2	3.1	2.5	Hypoendemic
**Plateaux**	136	16	27	25	–	68	22.2	28.6	
Agou	13	1	–	1	–	11	1.4	2.1	Hypo- to Hyperendemic
Akébou	2	1	–	1	–	–	1.0	1.4	–
Amou	8	–	1	1	–	6	1.7	2.6	Hypo- to Hyperendemic
Anié	12	2	3	2	–	5	1.5	1.5	Hypo- to Hyperendemic
Danyi	5	0	3	–	–	2	0.6	0.9	Meso- to Hyperendemic
Est-Mono	15	4	7	4	–	–	2.0	3.0	–

Supplementary Table 1. Continued

**Region** Prefecture[a]	**Total No. of villages surveyed**	**Endemicity level according to baseline microfilarial prevalence (BMP)**				**Without recorded BMP (%)**	**Proportion of overall population** (%)	**Proportion of rural population**[b] (%)	**Observations** (Modelled prevalence trends followed by villages without BMP)
		**Hypoendemic**	**Mesoendemic**	**Hyperendemic**	**Holoendemic**				
Haho	21	3	3	3	–	12	4.0	5.5	Hypo- to Hyperendemic
Kloto	5	2	–	–	–	3	2.3	1.7	Hyperendemic
Kpélé	2	–	1	–	–	1	1.2	1.8	Hyperendemic
Moyen-Mono	6	1	2	3	–	–	1.2	1.8	–
Ogou	37	1	5	9	–	22	3.7	4.0	Hypo- to Hyperendemic
Wawa	10	1	2	1	–	6	1.6	2.3	Hypo- to Hyperendemic
**Maritime**	41	6	–	1	–	34	42.0	25.7	
Avé	5	1	–	–	–	4	1.6	2.4	Hypo- to Hyperendemic
Bas-Mono	2	–	–	–	–	2	1.4	2.1	Mesoendemic
Golfe, including Lomé and Agoè-Nyivé	0	–	–	–	–	–	25.3	2.4	Non-endemic
Lacs	0	–	–	–	–	–	2.8	3.8	Non-endemic
Vo	0	–	–	–	–	–	3.4	5.0	Non-endemic

Supplementary Table 1. Continued

**Region** Prefecture[a]	**Total No. of villages surveyed**	**Endemicity level according to baseline microfilarial prevalence (BMP)**				**Without recorded BMP (%)**	**Proportion of overall population** (%)	**Proportion of rural population**[b] (%)	**Observations** (Modelled prevalence trends followed by villages without BMP)
		**Hypoendemic**	**Mesoendemic**	**Hyperendemic**	**Holoendemic**				
Yoto	22	2	–	1	–	19	2.7	3.7	Hypo- to Hyperendemic
Zio	12	3	–	–	–	9	4.8	6.3	Hypo- to Mesoendemic
**Total**	**400**	**41**	**62**	**43**	**2**	**252**	**100**	**100**	–

[a]Over the past 15 years, Togo has undergone administrative changes affecting its prefectures. In 2012, Tône was split into Tône and Cinkassé; Lacs was divided into Lacs and Bas-Mono (with the latter being historically known as the original onchocerciasis-endemic area of Lacs); Kloto was separated into Kloto and Kpélé; Ogou was divided into Ogou and Anié, and Wawa was divided into Wawa and Akébou. In 2018-19, Kpendjal was separated into Kpendjal-Ouest and Kpendjal; Oti was divided into Oti and Oti-Sud; Sotouboua was split into Sotouboua and Mô, and Lomé Capital comprised 5 prefectures.

[b]Proportion of the rural population of the total rural population in Togo.

[c]Recorded in 1970-74 preparatory surveys prior to the commencement of the OCP [1,7] (not in OCP database).

1.2 History of onchocerciasis control in Togo

Prior to control interventions, most of Togo was classified as meso- to holoendemic for onchocerciasis, with only the southernmost Maritime region classified as mostly hypoendemic (Supplementary Fig. 1b). The country's two principal river basins, the Oti (also known as Pendjari or Oti/Pendjari, with main tributaries including the Kara, Kéran and Mô rivers) and the Mono (with tributaries such as the Amou, Anié, Kra, Haho, Ogou, Yoto and Zio rivers), had some of the highest annual biting rates in the country (reaching up to 100,000 bites/person/year, Supplementary Table 4). Other, smaller river basins, in which onchocerciasis endemicity has been documented, include the White Volta (Volta Blanche) and the Todzie rivers, as well as tributaries of Lake Volta (Asukawkaw, Gban Hou and Kpaza Koue rivers) and Lake Togo (Haho, Yoto and Zio rivers).

Most of the Togolese territory was gradually incorporated in the OCP (Supplementary Fig. 2a), firstly, in the programme's Phase II (upper left corner of Savanes), second in the Phase III East (rest of Savanes, Kara and upper part of Centrale), and finally in the Southern Extension (rest of Centrale, Plateaux and most of Maritime), beginning vector control (VC) with aerial larviciding in January 1976, March 1977 and February 1988, respectively [5]. The Southern Extension aimed to tackle the reinvasion of areas under VC by *Simulium damnosum* s.l. [8,9]. In 1987, delivery of annual ivermectin mass drug administration (MDA) started in the OCP area, in the first instance by mobile teams, and ultimately by community-directed distribution of ivermectin (CDTI), which aimed to expand geographical coverage and increase sustainability [9]. Combined larviciding and ivermectin treatment lowered transmission substantially, effecting a 90% reduction of the annual transmission potential (ATP, number of L3 larvae per person per year) after the first two years of implementation in some areas [8]. However, control was not effective in interrupting transmission around the Oti tributaries (Kara, Keran and Mô River basins) in Togo [8], where the entomo-epidemiological situation remained unsatisfactory, namely, in Kara and parts of Savanes and Centrale. Therefore, following the closure of the OCP in December 2002, some of Togo's persistent foci were included in Special Intervention Zones (SIZ) that were launched in December 2002 (Supplementary Fig. 2b), in which VC continued until 2007 and biannual ivermectin MDA was introduced from 2003 until 2012 [10,11].

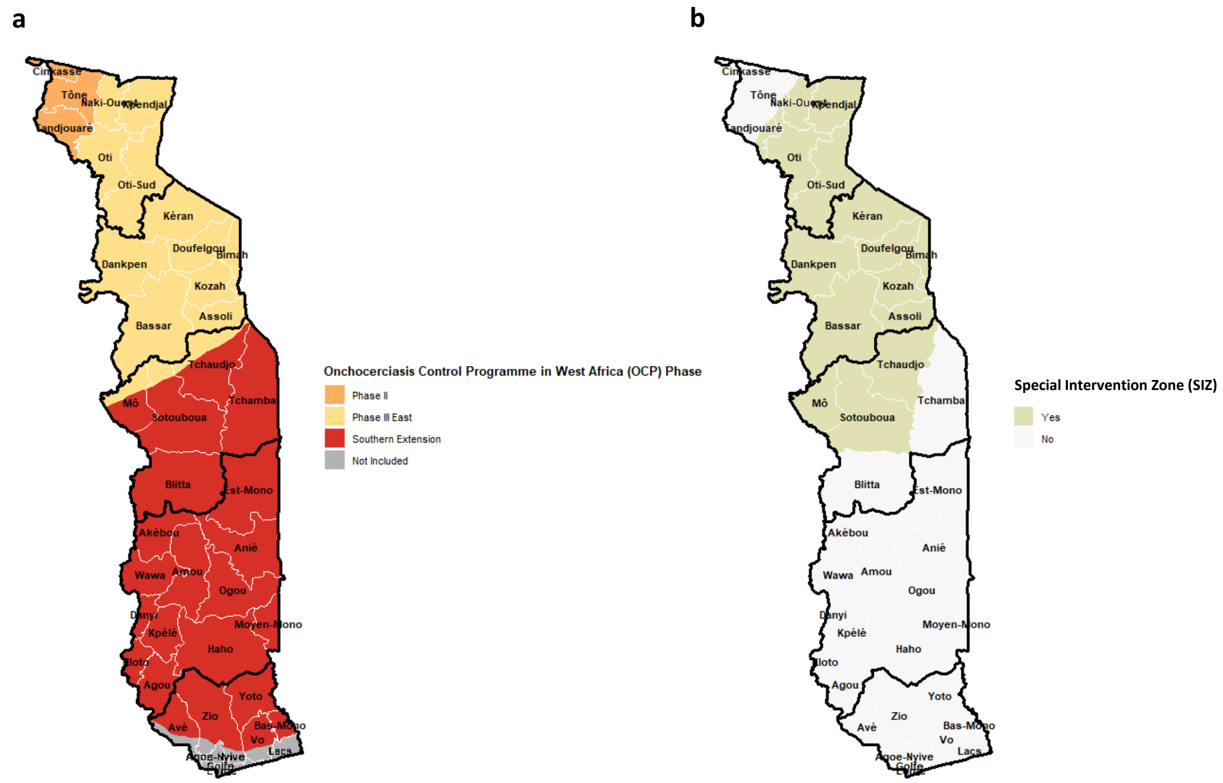

Supplementary Figure 2. Onchocerciasis control in Togo. (a) Phases under the Onchocerciasis Control Programme in West Africa (OCP). (b) Special Intervention Zone (SIZ). Black thick borders indicate regions; white thin borders denote current prefectures. Data obtained from sources cited in Text S2 for OCP and SIZ. The maps for Togo, and its regions and prefectures were drawn using the R package geodata version 0.6-2 (https://github.com/rspatial/geodata).

In particular, aerial VC was extended in the Upper Oti River Basin of Savanes, where it was deemed to be necessary to further reduce prevalence and mitigate the risk of infection resurgence, and in all the major river basins of Kara, as well as the Mô River Basin of Centrale, until 2007 [12]. Concurrently with VC, biannual CDTI was implemented until 2012 in 11 SIZ prefectures with historically high onchocerciasis prevalence [12]. A portion of southeast Tône was also part of the SIZ but only received VC. Currently, the former SIZ area comprises 16 prefectures, as Tône was divided into Tône and Cinkassé; Kpendjal was divided into Kpendjal and Kpendjal-Ouest (Naki-Ouest); Oti into Oti and Oti-Sud, and Sotouboua into Sotouboua and Mô prefectures.

Supplementary Fig. 3 illustrates trends in (crude) microfilarial prevalence from 1975 to 2017 in villages located in SIZ and non-SIZ areas. As the launch of SIZ started in December 2002, prior to this date all the villages would have been part of the OCP, but the colour coding helps to visualise the evolution of the prevalence situation from the beginning of the programme. Towards the end of the OCP, in the period between 1998 and 2002, the epidemiological

situation in some villages was of great concern, with some villages exhibiting microfilarial prevalence as high as 60% in the would-be SIZ villages in contrast with a maximum of 25% in those which were not included in the SIZ. By 2007 (end of the extended VC period in SIZ areas), prevalence had declined to levels comparable to those of non-SIZ villages by the closure of the OCP.

Supplementary Fig. 4 presents box-and-whiskers plots comparing crude microfilarial prevalence between SIZ and non-SIZ villages for the periods of: a) 1998–2002 (nearing the end of the OCP), b) 2007–2011 (post-OCP and, in SIZ, with continuation of VC until 2007 and switch to biannual MDA from 2003), and c) 2012 –2017 (without VC but with continuation of biannual MDA in SIZ and of annual MDA in non-SIZ, with the exception of some areas of Plateaux (all non-SIZ) which switched to biannual MDA in 2014). In 1998–2002 (Fig. 4a), microfilarial prevalence (compared using the Mann-Whitney U test) was significantly higher in the would-be SIZ villages compared to those which were not included in SIZ (p-value <0.001). In 2007–2011 (Fig. 4b), microfilarial prevalence in SIZ villages decreased significantly compared to 1998–2002 (p-value <0.001) but remained higher than in non-SIZ (p-value = 0.008). In 2012–2017 (Fig. 4c), prevalence in SIZ villages increased significantly compared to 2007–2011 (p-value = 0.005), and was higher than in non-SIZ villages (p-value <0.001).

After the closure of the OCP and the SIZ, the Ministry of Health (MoH) of Togo maintained annual or biannual MDA as they were during the SIZ [13]. Since 2002, the reported therapeutic coverage of ivermectin has been around 80% (of the total population) recommended for onchocerciasis elimination of transmission (EOT) [13]. Since 2014, the National Onchocerciasis Control Programme (NOCP) of the MoH extended biannual MDA from the 11 prefectures that were part of the SIZ to 16 prefectures, to include five additional prefectures in the Plateaux region, which at the time had microfilarial prevalence values exceeding 5% [14,15]. Villages with more than 2,000 inhabitants had not been incorporated in ivermectin MDA programmes until 2018-2020, as they were deemed to have a lower blindness risk [15,16]. A detailed record of onchocerciasis control history by region and prefecture is described in Supplementary Table 2.

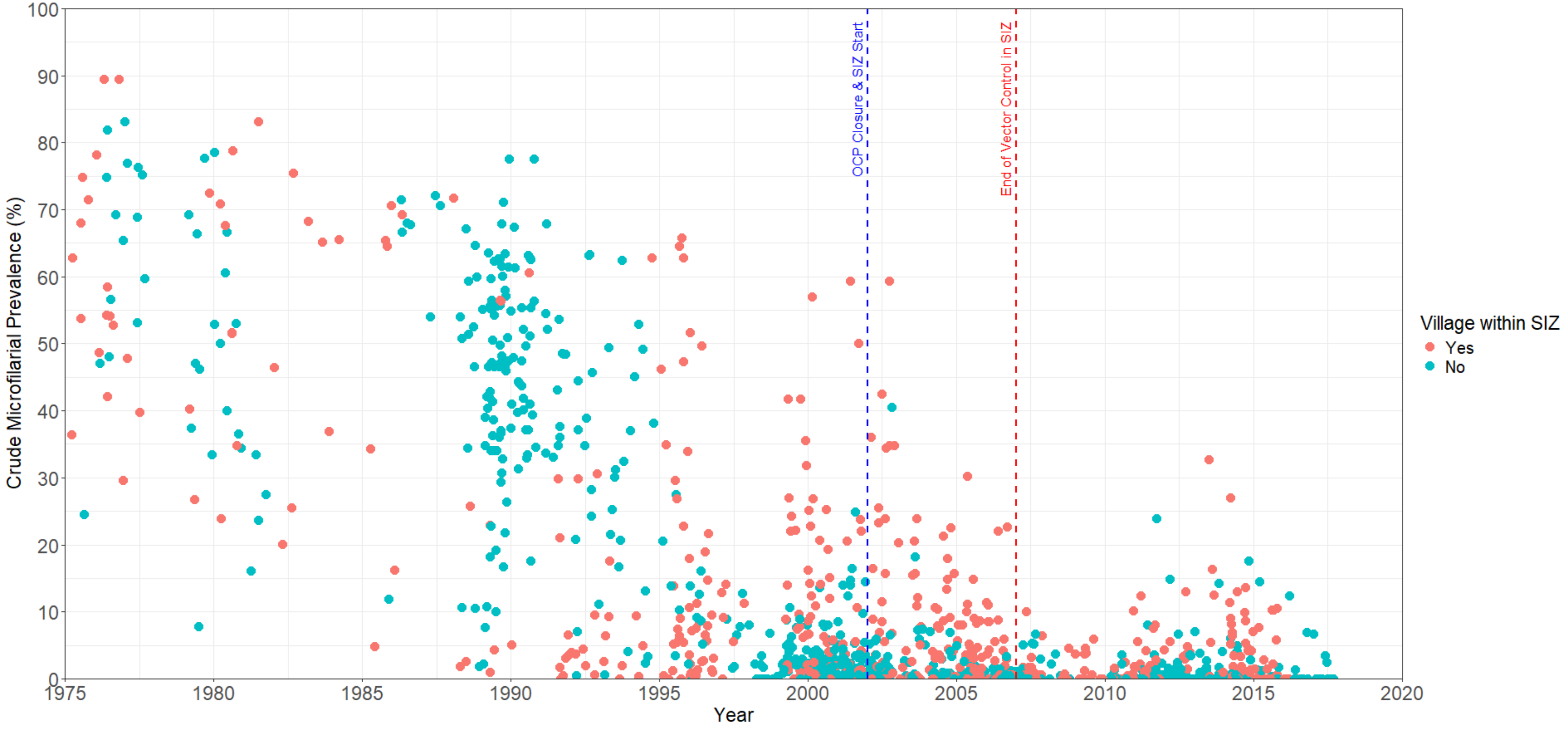

Supplementary Figure 3. Temporal trends of crude microfilarial prevalence in villages located in Special Intervention Zone (SIZ) and non-SIZ areas from 1975 to 2017. Red circles represent villages located in SIZ areas; blue circles represent villages located in non-SIZ areas. The blue vertical dashed line indicates the end of the Onchocerciasis Control Programme in West Africa (OCP) and the start of SIZ in 2002; the red vertical dashed line indicates the end of vector control in SIZ villages.

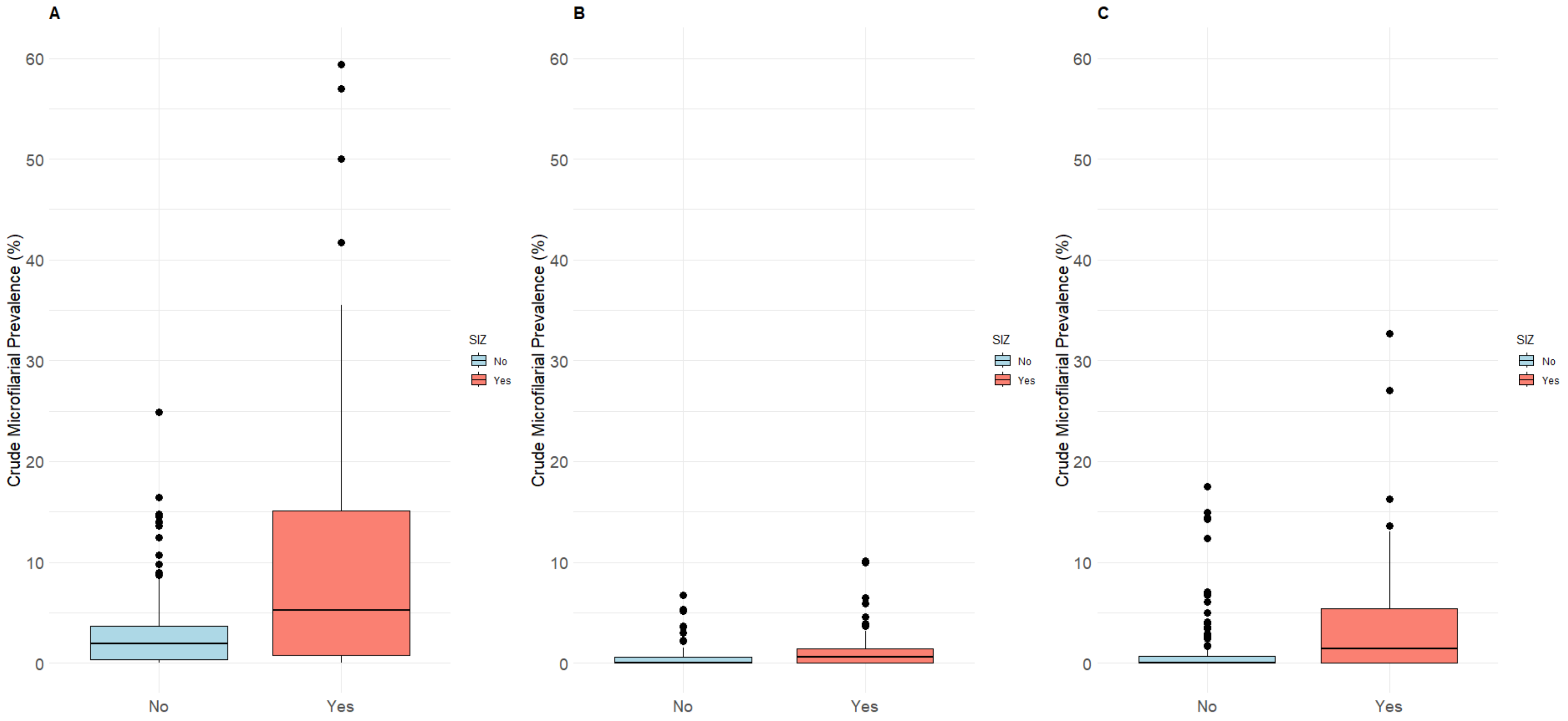

Supplementary Figure 4. Box-and-whisker plots of crude microfilarial prevalence in non-SIZ (No) and SIZ (Yes) villages in Togo across different intervention periods. (a) 1998–2002 (nearing the end of the OCP). (b) 2007–2011 (post-OCP and, in SIZ, at the end of VC but continuing biannual MDA). (c) 2012–2017 (under biannual and/or annual MDA but without VC). The horizontal lines within each box represent the median microfilarial prevalence, with the lower and upper box edges indicating the 25th and 75th percentiles, respectively (interquartile range, IQR). Whiskers extend to 1.5 times the IQR, and the black dots indicate outliers.

Supplementary Table 2. History of onchocerciasis control between 1975 and 2018 per region and prefecture in Togo

**Region** Prefecture (no. villages surveyed)	**OCP phase**	**SIZ**	**Aerial larviciding Vector Control (VC)**		**Ivermectin MDA**		**Observations**
			**Start year**	**End year**	**Start year**	**Biannual MDA and stop-MDA**	
**Savanes (49)**	II and III E	Mostly	1976 (7%)<br>1977 (93%)	1993	1993	Biannual MDA since 2003 in three prefectures<br>Stop-MDA considered since 2018	Western parts of Tône, Tandjouaré and Oti prefectures were included in OCP Phase II<br>In some prefectures, MDA might have started later, as control focused on VC<br>VC ended either in 1993 or at the beginning of 1994.<br>Evidence of ongoing transmission in 2022 (anti-Ov16 ELISA seroprevalence in children samples in Tône, Tandjouaré and Kpendjal above 0.1% threshold) [17]
Kpendjal, including Kpendjal-Ouest (9)[a]	III E	Mostly (90%)	1976 (22%)<br>1977 (78%)	1993	1993	Biannual MDA since 2003	Focal control on a hyperendemic focus until 2007<br>MDA may have started sooner (between 1988 and 1993)
Oti, including Oti-Sud (29)[a]	II (5%)<br>III E (95%)	Yes	1977	1993	1993	Biannual MDA in 1993 and since 2003	–
Tandjoaré or Tandjouaré (5)	III E	Yes	1977, possibly 1976	1993	NA	Biannual MDA in 2004, 2005 and from 2007 to 2011	Tandjouaré was later included in the SIZ to participate in the biannual MDA
Tône, including Cinkassé (6)[a]	III E	No	1976 (25%)<br>1977 (75%)	1993	NA	No	Includes two villages with no follow-up surveys and a baseline endemicity similar to Samomoni village [5]

Supplementary Table 2. Continued

**Region** Prefecture (no. villages surveyed)	**OCP phase**	**SIZ**	**Aerial larviciding Vector Control (VC)**		**Ivermectin MDA**		**Observations**
			**Start year**	**End year**	**Start year**	**Biannual MDA and stop-MDA**	
**Kara (88)**	III E	Yes	1977	1993/2002 /2007	1988 (75%) 1992 (5%) 1995 (20%)	Biannual MDA since 2003 in all prefectures	Most but possibly not all the river basins in Kara had VC until 2007 (potentially ending in 1993 and 2002). However, it was not possible to be precise, as each prefecture is under the influence of several water courses. Therefore, for modelling purposes, it was assumed that VC ended in 2007
Assoli (3)	III E	Yes	1977	1993/2002 /2007	1992	Biannual MDA since 2003	–
Bassar (17)	III E	Yes	1977	2002/2007	1988	Biannual MDA in 1992, 1995, 1998 and since 2003	Besides the prefecture interventions, VC was implemented at least in 1981-1988 in those river basins (Kassa River) where the Djodji form of *Simulium sanctipauli* (a highly competent vector of *O. volvulus*) had been found [18]
Binah or Bimah (4)	III E	No	1977	2002/2007	1992	Biannual MDA since 2003	–
Dankpen (19)	III E	Yes	1977	1993/2002 /2007	1995	Biannual MDA since 2003	–

Supplementary Table 2. Continued

**Region** Prefecture (no. villages surveyed)	**OCP phase**	**SIZ**	**Aerial larviciding Vector Control (VC)**		**Ivermectin MDA**		**Observations**
			**Start year**	**End year**	**Start year**	**Biannual MDA and stop-MDA**	
Doufelgou (5)	III E	Yes	1977	1993/2002 /2007	1988	Biannual MDA in 1988, 1992 and since 2003	–
Kéran (13)	Mostly III E	Yes	1977	2002/2007	1988	Biannual MDA in 1996 and since 2003<br>Triannual MDA in 1993	–
Kozah (27)	III E	Yes	1977	2002/2007	1988	Biannual MDA in 1988, 1992, 1998 and since 2003	–
**Centrale (86)**	III E and SE	Partially	1977 (20%)<br>1988 (5%)<br>1989 (75%)	2002 (70%)<br>2007 (30%)	1991	Biannual MDA since 2003 in two prefectures	–
Blitta (28)	SE	No	1988 (4%)<br>1989 (96%)	2002	1991	No	Besides the prefecture interventions, VC was implemented at least in 1981-1988 in those river basins (Anié and Arukaukau Rivers) where the Djodji form of *Simulium sanctipauli* (a highly competent vector of *O. volvulus*) had been found [18]

Supplementary Table 2. Continued

**Region** Prefecture (no. villages surveyed)	**OCP phase**	**SIZ**	**Aerial larviciding Vector Control (VC)**		**Ivermectin MDA**		**Observations**
			**Start year**	**End year**	**Start year**	**Biannual MDA and stop-MDA**	
Sotouboua, including Mô (34)[a]	III E (40%)<br>SE (60%)	Partially (40%)	1977 (40%)<br>1988 (6%)<br>1989 (54%)	2002 (60%)<br>2007 (40%)	1991	Biannual MDA since 2003	Besides the prefecture interventions, VC was implemented at least in 1981-1988 in those river basins (Kpaza Koue, Anié and Arukaukau Rivers) where the Djodji form of *Simulium sanctipauli* (a highly competent vector of *O. volvulus*) had been found [18]<br>Part of the rivers of this prefecture were included in the SIZ until 2007 (Mô River basin).
Tchamba (16)	SE	No	1989	2002	1991	No	–
Tchaoudjo or Tchaudjo (8)	III E (20%)<br>SE (80%)	Partially (20%)	1977 (22%)<br>1988 (33%)<br>1989 (45%)	2002 (80%)<br>2007 20%)	1991	Biannual MDA since 2003	–
**Plateaux (136)**	SE	No	1976 (1%)<br>1988 (10%)<br>1989 (89%)	2002	1991 (55%)<br>1992 (30%)<br>1993 (5%)	Biannual MDA since 2014 in four prefectures	–
Agou (13)	SE	No	1988	2002	1991	No	–

Supplementary Table 2. Continued

**Region** Prefecture (no. villages surveyed)	**OCP phase**	**SIZ**	**Aerial larviciding Vector Control (VC)**		**Ivermectin MDA**		**Observations**
			**Start year**	**End year**	**Start year**	**Biannual MDA and stop-MDA**	
Akébou, including areas previously from Wawa (2)[a]	SE	No	1989	NA	1993	No	–
Amou (8)	SE	No	1989	2002	1992	Biannual MDA since 2014	Besides the prefecture interventions, VC was implemented at least in 1981-1988 in those river basins (Anié River) where the Djodji form of *Simulium sanctipauli* (a highly competent vector of *O. volvulus*) had been found [18]
Anié, including areas previously from Ogou (12)[a]	SE	No	1989	2002	1991	Biannual MDA in 1993, 1995 and 1996	Besides the prefecture interventions, VC was implemented at least in 1981-1988 in those river basins (Anié River) where the Djodji form of *Simulium sanctipauli* (a highly competent vector of *O. volvulus*) had been found [18]
Danyi (5)	SE	No	1976 (25%) 1989 (75%)	2002	1993	Biannual MDA since 2014	Besides the prefecture interventions, VC was implemented at least in 1981-1988 in those river basins (Anié and Gban-Houa/Wawa Rivers) where the Djodji form of *Simulium sanctipauli* (a highly competent vector of *O. volvulus*) had been found [18]
Est-Mono (15)	SE	No	1989	2002	1991	No	–

Supplementary Table 2. Continued

**Region** Prefecture (no. villages surveyed)	**OCP phase**	**SIZ**	**Aerial larviciding Vector Control (VC)**		**Ivermectin MDA**		**Observations**
			**Start year**	**End year**	**Start year**	**Biannual MDA and stop-MDA**	
Haho (21)	SE	No	1988 (38%) 1989 (62%)	2002	1992	Biannual MDA since 2014	–
Kloto, may include areas of Kpélé (5)	SE	No	1989	2002	1993	No	–
Kpélé, including areas previously from Kloto (2)[a]	SE	No	1988	2002	1993	No	–
Moyen-Mono (6)	SE	No	1989	2002	1992	Biannual MDA in 1993	–
Ogou, may include areas of Anié (37)[a]	SE	No	1989	2002	1991	Biannual MDA in 1992 and since 2014 Triannual MDA in 1993	Besides the prefecture interventions, VC was implemented at least in 1981-1988 in those river basins (Anié River) where the Djodji form of *Simulium sanctipauli* (a highly competent vector of *O. volvulus*) had been found [18]
Wawa, may include areas from Akébou (10)[a]	SE	No	1988 (13%) 1989 (87%)	2002	1991	No	Besides the prefecture interventions, VC was implemented at least in 1981-1988 in those river basins (Anié, Gban-Houa/Wawa, Domi and Ove Rivers) where the Djodji form of *Simulium sanctipauli* (a highly competent vector of *O. volvulus*) had been found [18,19]

Supplementary Table 2. Continued

**Region** Prefecture (no. villages surveyed)	**OCP phase**	**SIZ**	**Aerial larviciding Vector Control (VC)**		**Ivermectin MDA**		**Observations**
			**Start year**	**End year**	**Start year**	**Biannual MDA and stop-MDA**	
**Maritime (41)**	SE	No	1988 (97%) 1989 (3%)	2002	1993	Some prefectures implemented stop-MDA surveys in 2014, 2018 or 2020.	–
Avé (5)	SE	No	1988	2002	1993	No<br>Stop-MDA assessment successful in 2022 [20]	–
Bas-Mono, including areas previously from Lacs (2)[a]	SE	No	1988	2002	1993	Stop-MDA assessment (2014-2017) detected ongoing transmission. MDA was re-started in 2017 [20]. Stop-MDA assessment successful in 2022 [20]	–
Golfe, including Lomé and Agoè-Nyivé (0)[a]	SE	No	–	–	–	The stop-MDA assessment (2014-2017) completed in 2017 was successful [20]	Non-endemic for onchocerciasis; most of the prefecture did not need control. However, some focal foci had low prevalence at baseline (no updated information)
Lacs (0)	SE	No	1989	2002	1993	Started stop-MDA assessment in 2018, which was successful [20]	The historically known endemic part of Lacs was recently divided into Lacs and Bas-Mono. Only Bas-Mono is endemic

Supplementary Table 2. Continued

**Region** Prefecture (no. villages surveyed)	**OCP phase**	**SIZ**	**Aerial larviciding Vector Control (VC)**		**Ivermectin MDA**		**Observations**
			**Start year**	**End year**	**Start year**	**Biannual MDA and stop-MDA**	
Vo (0)	SE	No	–	–	–	The stop-MDA assessment (2014-2017) completed in 2017 was successful [20]	Non-endemic for onchocerciasis by 2006
Yoto (22)	SE	No	1988	2002	1993	No<br>Stop-MDA assessment detected ongoing transmission in 2022 [20]. Biannual MDA considered in 2023-2024.	–
Zio (12)	SE	No	1988 (100%)	2002	1993	No<br>Stop-MDA assessment successful in 2022 [20]	–
**All prefectures (400)**							

OCP, Onchocerciasis Control Programme in West Africa; SIZ, Special Intervention Zone; VC. Vector control; MDA, Mass Drug Administration with ivermectin.

[a]Over the past 15 years, Togo has undergone administrative changes affecting its prefectures. In 2012, Tône was split into Tône and Cinkassé; Lacs was divided into Lacs and Bas-Mono (with the latter being historically known as the original onchocerciasis-endemic area of Lacs); Kloto was separated into Kloto and Kpélé; Ogou was divided into Ogou and Anié, and Wawa was divided into Wawa and Akébou. In 2018-19, Kpendjal was separated into Kpendjal-Ouest and Kpendjal; Oti was divided into Oti and Oti-Sud; Sotouboua was split into Sotouboua and Mô, and Lomé Capital comprised 5 prefectures.

Supplementary Text 2. Data sources

Our study involved the integration of two national databases containing geographical, epidemiological and historical control information on VC and ivermectin MDA. The data were obtained from the OCP (EPICROSS) database, publicly available from Vinkeles Melchers et al. [15] and progress reports [21-24], SIZ reports, MoH of Togo reports, World Health Organization and Expanded Special Project for Elimination of Neglected Tropical Diseases (WHO-ESPEN), as well as from academic publications [13,25-30]. Data curation was conducted to address inconsistent formats and ensure compatibility of datasets prior to their analyses and modelling using R version 4.4.1 [31], and RStudio version 2024.04.2 [32], and Imperial College Research Computing Service [33]. Changes in Togo's prefecture organisation over time (see footnote of Supplementary Table 2) were tracked for the modelling. Data were primarily utilised at the village level within prefectures. Initial ivermectin MDA records (1988-2018) were only available at the prefecture level. The distribution of surveyed villages is presented in Supplementary Fig. 5. The relationship between crude and age- and sex-standardised microfilarial prevalence is presented in Supplementary Fig. 6.

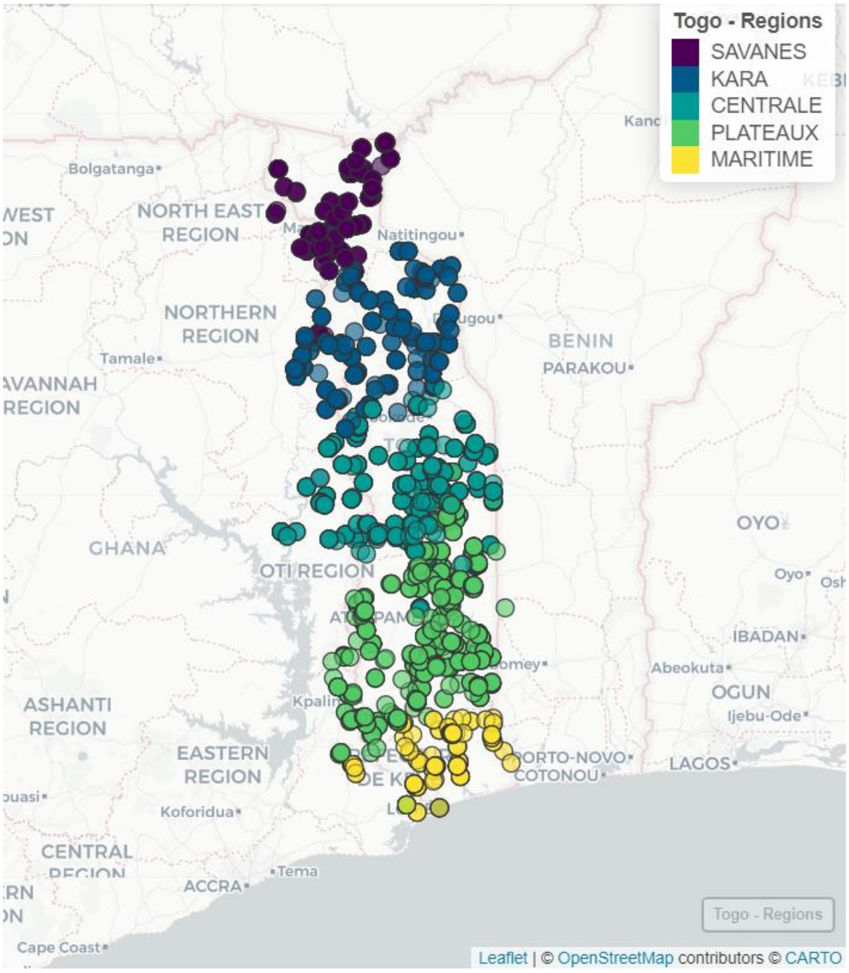

Supplementary Figure 5. Geographical distribution of villages for onchocerciasis monitoring across regions in Togo. Village GPS coordinates show approximate locations.

Relationship between crude and age- and sex-standardised microfilarial prevalence

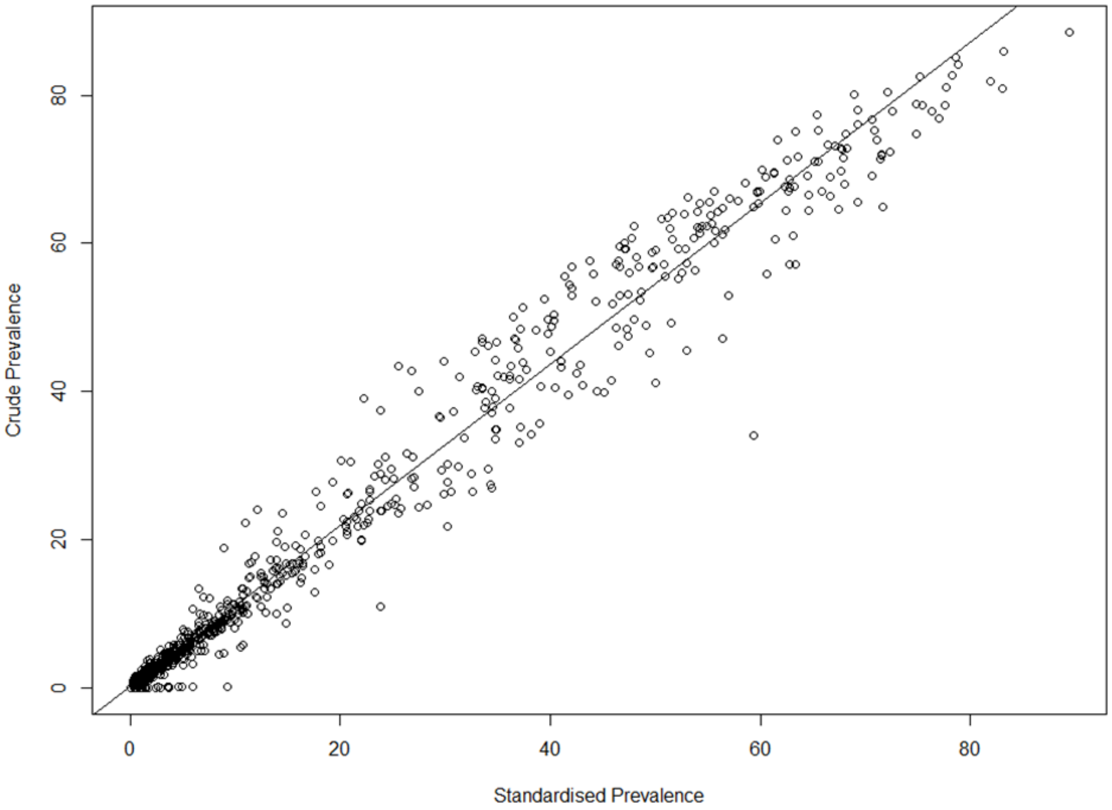

Supplementary Figure 6. Linear relationship between crude microfilarial prevalence and age- and sex-standardised microfilarial prevalence (n = 1,612 surveys). Pearson's correlation coefficient = 0.99, p-value <0.0001.

Supplementary Text 3. Estimation of Annual Biting Rates (ABRs)

The EPIONCHO-IBM transmission model reproduces the strongly non-linear relationship that has been observed between microfilarial prevalence and annual biting rate (ABR, number of bites/person/year) at pre-intervention endemicity conditions [34,35]. Calculation of ABR values to capture each baseline microfilarial prevalence (hypo- to holoendemic) level is necessary for simulating onchocerciasis dynamics [36]. The determined ABR values are given in Supplementary Table 3 assuming a value of $k_E = 0.3$ for the inter-individual exposure heterogeneity parameter and its associated parasite density-dependence parameters [36,37]. The high ABR values modelled that correspond to hyper- and holoendemicity are consistent with observed ABRs before the start of anti-vectorial interventions in Togo (Supplementary Table 4). ABRs measured at vector capture points (typically close to vector breeding sites) are likely higher than those at the villages [34].

Supplementary Table 3. Modelled annual biting rate (ABR) for each pre-control endemicity (microfilarial baseline prevalence) level

**Modelled baseline microfilarial prevalence (BMP, %)** (endemicity level)	**Modelled annual biting rate (ABR, bites/person/year)** (range)[a]
30 (hypoendemicity)	290 (240–450)
50 (mesoendemicity)	615 (430–1,054)
70 (hyperendemicity)	2,200 (1,210–6,320)
90 (holoendemicity)	60,000 (46,000–137,000)

[a]Range of annual biting rates (ABRs) sampled using the EPIONCHO-IBM model for hypoendemicity (30% BMP), mesoendemicity (50% BMP), hyperendemicity (70% BMP) [37], and holoendemicity (90% BMP).

Only the ABR values (not the ranges) were used in this work, but the ranges are included here to provide a better appreciation of the variability in ABR values associated with a given BMP.
NB: For 85% BMP, the mode (and range) of ABR values sampled using EPIONCHO-IBM is 22,000 (6,000–60,000) [38], illustrating that at the upper end of microfilarial prevalence, the relationship between ABR and BMP is very non-linear [35,36].

Supplementary Table 4. Annual biting rates measured at vector capture points prior to vector control, and baseline microfilarial prevalence (BMP) in surveyed villages

**River basin [Reference]**	**Vector capture point**	**Baseline annual biting rate (ABR, bites/person/year) at vector capture point** (survey year)	**Age- and sex-standardised mean BMP in village (%)** (range)	**Surveyed villages** (survey year)
Kara/Oti [39,40]	Landa-Pozanda	27,951 (1976-77)	83 (NA)	Landa-Pozanda (1976)
	Sarakawa Kpelou	14,538 (1976-77)	65 (62 – 68)	Anima (1976); Leon (1976)
Kéran/Oti [39,40]	Naboulgou	17,364 (1976-77)	NA	Several villages without recorded BMP following hyper- to holoendemic trends
	Sola	7,379 (1976-77)	NA	A village without recorded BMP (Sola) following a holoendemic trend
	Tapoundé	9,128 (1976-77)	NA	Villages without recorded BMP following hypo- to hyperendemic trends
	Titira	28,266 (1976-77)	89 (NA)	Tchitchira/Titira (1976)
Kéran/Oti (Binah/Bimah) [39]	Pouda	13,945 (1977-77)	NA	Villages without recorded BMP following meso- to hyperendemic trends
Koumongou/Oti [39]	Korontiere and Kouporgon	7,255 (1977-77)	50.1 (NA)	Fare (1976). Villages without recorded BMP following hypo- to holoendemic trends
Mono [41,42]	Atchinedji	54,283 (1978–1981)	74 (64 – 83)	Adouroukopé/Assanté (1990); Oniakopé (1977)

Supplementary Table 4. Continued

River basin [Reference]	Vector capture point	Baseline annual biting rate (ABR, bites/person/year) at vector capture point (survey year)	Age- and sex-standardised mean BMP in village (%) (range)	Surveyed villages (survey year)
Mono [41,42]	Kpessi	46,764 (1978–1981)	52 (38 – 65)	Alemondji (1990); Atotoie (1990); Babame (1990); Kodjodakopé (1990); Kokote (1989); Konta (1990); Maroukou II (1990); Tchankpa (1990); Yambakopé (1990)
	Landa Mono	39,894 (1978–1981)	59 (46 – 67)	Bodowda (1990); Boungolo (1989); Djomé (1977); Kaza (1990); Kassikide (1989); Kendjeria (1990); Laoude (1990); Landa-Mono (1989); Mono 1 (1989); Sessaro (1990); Souroutawi (1989)
	Tetetou/Tététou	106,325 (1978–1981)	62 (26 – 82)	Aglamassoe (1990); Diome (1977); Djikame (1990); Hoevime (1990); Kpodji (1989); Siyime (1989); Tetetou (1977)
Mono (Amou) [41,42]	Amou-Oblo	33,514 (1978–1981)	54 (11 – 73)	Abouloukopé (1989); Adjabouloukoukopé (1989); Afikopé (1989); Agote (1989); Amouta (1980); Aroukakopé (1989); Fedigbe/Fétigbé (1990); Safou-Kopé Atiba (1990); Wetrope (1989)
Mono (Anié) [41,42]	Fazao	24,675 (1978–1981)	52 (44 – 60)	Fazao (1977); N'Djavezi (1990)
	Pagala	13,795 (1978–1981)	58 (25 – 79)	Agodeka (1990); Anamanie (1990); Katakpe (1990); Kpawa (1990); Niama-Niama (1990); Tchanie (1990); Yoloum (1990)
	Alamassou/Alamansou	58,334 (1978–1981)	86 (NA)	Alamassou (1977)

Supplementary Table 4. Continued

**River basin [Reference]**	**Vector capture point**	**Baseline annual biting rate (ABR, bites/person/year) at vector capture point** (survey year)	**Age- and sex-standardised mean BMP in village (%)** (range)	**Surveyed villages** (survey year)
Mô/Oti [29,40]	Bagan/Bangan/Banghan/ Baghan	46,983 (1976-77)	70 (68 – 72)	Bangan (1976); Mo-village (1975)
	Bouzalo/Mo	40,919 (1976-77)	73.5 (72 – 75)	Bouzalo (1975); Sagbadai (1980)
	Kéméni/Aleheride	22,557 (1976)	53 (NA)	Kemini/Kéméni (1976)
Mô/Oti (Aleheride/Boualé) [39]	Aleheride/Alèchéridé	28,342 (1976-77)	53.4 (NA)	Kemini
Mô/Oti (Kama) [39]	Pont Kama	16,174	65.4 (NA)	Bigabo (1976). Another village without recorded BMP (Saboundi) following a hyperendemic trend.
Ogou/Mono [41,42]	Sirka	16,486 (1978–1981)	41 (13 – 62)	Adibo (1990); Dote-Copé (1990); Efoufami-Yeye (1990); Flama (1990); Gbagbadjakou I (1990); Nangbeto-Asanté (1990); Tele-Kopé (1990)
Oti (Porga) [39]	Porga Pont	12,367 (1976-77)	53.4 (NA)	A village without recorded BMP (Sougtangou) following a hyperendemic trend.
Volta Lac [Gban-Houa] [41-43]	Djodji[a]	138,026 – 246,125 (1978–1981)	61 (44 – 78)	Azigo (1990); Dayes-Dodzi (1980)
Zio [34]	Tokpo, other villages	7,102 (1980)	85.1 (NA)	Tokpo (1980)

[a]The site of Djodji presented the highest transmission potentials (ATP = 2,157) [34,43] of the Eastern extension until the Djodji form of *Simulium sanctipauli* sensu stricto was eliminated as a result of larviciding [18].

Supplementary Text 4. Minimal, reference and enhanced intervention scenarios

Three distinct levels of control interventions: “minimal”, “reference”, and “enhanced” were used in the simulations, based on variations in VC efficacy, MDA therapeutic coverage and the proportion of systematic non-adherence (SNA, representing the proportion of the population that never receives ivermectin). The MDA coverage increased over time. Initially, MDA was delivered by mobile teams with lower coverage until 1995 [9]. Subsequently, during the remaining period of the OCP, CDTI aimed for a minimum coverage of 65% of the total population (approximately 80% coverage of eligibles) to attain EPHP (1996-2001), followed by a target coverage of 80% of the total population (100% of eligibles) for EOT (2002 to date). Although the efficacy of VC has exceeded 90% in several OCP regions [44,45], certain areas exhibited lower efficacy, such as in the mountainous region of Oti [46] and Kara. Consequently, for the minimal and reference scenarios, VC efficacies of 60% and 75% were assumed, respectively. The proportion of SNA was set at 1% for the enhanced scenario, as SNA tends to decrease with higher therapeutic coverage [36,47,48]. For the minimal and reference scenarios, the proportion of SNA was assumed to be 5% and 2.5%, respectively [49].

Reported ivermectin treatment coverage of total population (%) per region and prefecture from 1991 to 2018 in Togo

Supplementary Table 5. Reported coverage (% of total population) of ivermectin MDA for 1991-2018 in Savanes

Year	Prefecture				
	Cinkassé	Kpendjal	Oti	Tandjouaré	Tône
1991	0	0	0	0	0
1992	0	0	0	0	0
1993	0	NA	0	NA	NA
1994	0	NA	0	NA	NA
1995	0	NA	82.1	NA	NA
1996	0	NA	NA	NA	NA
1997	0	0	0	0	0
1998	0	NA	NA	NA	NA
1999	0	NA	NA	NA	NA
2000	0	69.8	70.3	79.0	68.0
2001	72.7	69.1	73.5	77.6	73.1
2002	NA	72.0	76.1	81.9	76.8
2003	NA	70.3	74.6	77.7	75.5
2004	NA	69.5	73.5	78.1	81.6
2005	NA	69.5	73.5	78.1	83.4
2006	NA	83.9	80.8	63.4	84.0
2007	NA	83.7	83.9	83.6	84.4
2008	NA	85.1	84.6	85.0	85.0
2009	NA	85.1	84.9	85.7	84.1
2010	NA	81.3	85.3	85.0	84.8
2011	NA	85.0	83.7	85.4	79.8
2012	NA	84.9	83.5	85.5	68.0
2013	80.5	83.0	80.8	85.9	82.6
2014	80.3	83.3	81.3	82.4	77.6
2015	81.7	85.1	82.2	83.0	81.5
2016	81.4	83.3	82.5	83.2	83.4
2017	81.7	82.3	80.7	79.2	82.7
2018	80.5	82.2	80.3	82.0	84.5

Some parts of Savanes may have started receiving ivermectin MDA in 1988-1990 [13]. Coverage data from [13,15]. NA: Not available.

Supplementary Table 6. Reported coverage (% of total population) of ivermectin MDA for 1991-2018 in Kara

Year	Prefecture						
	Assoli	Bassar	Binah	Dankpen	Doufelgou	Kéran	Kozah
1991	0	0	0	0	0	55.7	60.9
1992	65.2	63.5	68.2	0	65.9	59.6	71.5
1993	50.1	58.4	62.5	0	54.1	53.6	66.0
1994	NA	64.4	71.2	0	66.4	59.3	61.4
1995	82.5	73.0	71.5	64.1	71.9	73.0	76.5
1996	73.7	73.8	75.8	NA	77.4	75.5	82.6
1997	0	0	0	0	0	0	0
1998	83.9	79.8	87.7	80.4	81.8	71.8	75.9
1999	69.3	74.3	76.1	79.9	80.7	86.7	73.8
2000	66.4	69.4	79.3	75.5	77.8	77.5	75.6
2001	77.2	76.1	77.5	75.7	75.1	80.3	72.6
2002	80.7	85.0	77.6	75.2	82.4	79.7	80.0
2003	87.8	87.5	87.7	85.3	86.3	86.3	82.6
2004	87.1	86.3	86.9	85.6	84.4	85.7	85.7
2005	85.0	86.4	86.6	82.2	85.9	85.8	85.8
2006	85.8	86.5	85.3	85.8	85.6	85.5	87.0
2007	85.1	85.0	83.5	85.9	86.3	84.9	86.5
2008	86.8	84.5	84.8	85.3	86.3	85.6	86.7
2009	87.0	86.7	87.2	84.8	87.1	85.5	86.9
2010	86.2	80.4	86.9	83.8	86.6	85.4	83.8
2011	83.6	85.5	85.3	84.8	88.2	85.4	85.7
2012	85.9	83.7	83.3	85.2	85.8	85.6	85.3
2013	86.4	85.3	86.4	83.8	85.6	85.0	86.4
2014	81.9	79.9	82.5	81.2	77.9	85.1	79.1
2015	85.6	82.1	85.1	80.4	83.7	85.2	83.9
2016	82.2	82.4	85.2	80.8	81.5	82.7	82.6
2017	80.8	82.3	83.2	78.9	82.6	83.7	80.4
2018	81.6	81.8	84.8	78.1	81.1	81.5	84.7

Some parts of Kara may have started receiving ivermectin MDA in 1988-1990 [4]. Coverage data from [13,15].
NA: Not available.

Supplementary Table 7. Reported coverage (% of total population) of ivermectin MDA for 1991-2018 in Centrale

Year	Prefecture			
	Blitta	Sotouboua	Tchamba	Tchaoudjo
1991	62.8	59.3	62.1	57.6
1992	73.1	NA	NA	NA
1993	67.2	67.4	69.3	71.7
1994	58.6	54.8	59.8	58.5
1995	75.9	68.9	84.1	81.7
1996	82.6	81.5	87.9	86.8
1997	0	0	0	0
1998	NA	NA	NA	NA
1999	NA	NA	NA	NA
2000	73.7	72.1	75.4	68.5
2001	77.1	72.2	72.5	73.1
2002	76.3	72.2	72.9	75.8
2003	82.0	82.5	82.3	85.9
2004	83.3	84.0	85.8	86.4
2005	84.5	85.3	81.9	85.2
2006	85.8	79.4	84.0	84.5
2007	88.7	82.4	87.3	85.5
2008	88.3	86.1	86.6	85.7
2009	88.3	85.8	86.8	85.8
2010	86.4	81.8	80.1	84.2
2011	87.1	86.1	75.7	84.6
2012	85.2	59.4	89.3	84.7
2013	89.5	83.4	82.3	85.2
2014	76.6	83.6	74.5	80.3
2015	86.4	84.0	82.8	85.5
2016	84.4	84.0	82.6	84.6
2017	86.4	84.9	81.9	83.5
2018	82.5	85.8	NA	88.7

Coverage data from [13,15]. NA: Not available.

Supplemantary Table 8. Reported coverage (% of total population) of ivermectin MDA for 1991-2018 in Plateaux

Year	Prefecture											
	Agou	Akébou	Amou	Anié	Danyi	Est-Mono	Haho	Kloto	Kpélé	Moyen-Mono	Ogou	Wawa
1991	41.3	0	0	51.5	0	54.3	0	0	0	0	54.1	0
1992	NA	0	59.3	NA	0	73.3	0	0	0	0	66.0	91.3
1993	46.2	62.9	57.5	64.8	62.8	66.8	55.7	70.7	64.3	66.0	58.8	62.7
1994	72.1	65.7	59.7	45.4	67.7	58.2	68.1	75.0	74.7	60.3	60.7	64.9
1995	71.5	NA	76.4	69.7	83.9	79.0	73.0	63.0	NA	76.7	75.1	78.2
1996	78.2	81.6	80.7	80.8	90.4	80.5	19.1	74.2	NA	NA	80.0	75.8
1997	0	0	0	0	0	0	0	0	0	0	0	0
1998	NA	NA	NA	NA	NA	NA	NA	NA	NA	NA	NA	NA
1999	NA	NA	NA	NA	NA	NA	NA	NA	NA	NA	NA	NA
2000	53.7	NA	67.1	70.1	63.4	47.5	71.8	67.7	NA	71.0	55.4	62.4
2001	56.5	74.3	68.9	NA	73.3	50.6	68.3	73.3	76.5	77.1	65.6	58.1
2002	74.0	NA	73.8	NA	75.2	72.8	78.8	76.9	NA	73.7	76.1	73.6
2003	79.9	NA	77.9	NA	78.7	74.9	79.9	81.4	NA	80.5	74.0	70.9
2004	85.0	NA	81.4	NA	81.3	80.3	81.1	86.0	NA	85.1	84.1	80.7
2005	85.1	NA	85.9	NA	84.5	84.2	83.2	86.0	NA	85.5	83.1	81.1
2006	84.9	NA	85.1	NA	85.1	85.4	85.1	85.7	NA	85.6	83.7	85.5
2007	85.2	NA	85.6	NA	85.4	83.7	85.2	85.3	NA	84.2	85.6	85.2
2008	85.2	NA	85.4	NA	85.3	85.2	85.8	85.7	NA	86.0	87.0	84.8
2009	85.5	NA	86.9	NA	84.8	85.7	86.0	85.9	NA	88.1	85.2	85.3
2010	86.6	NA	85.7	NA	85.8	85.0	86.5	86.0	NA	85.7	85.3	85.1
2011	81.5	NA	84.5	NA	83.0	78.2	79.1	84.0	NA	81.5	83.8	80.5
2012	89.1	NA	84.2	NA	82.7	81.1	79.4	83.4	NA	84.8	82.2	81.2
2013	85.0	79.9	81.7	84.1	82.5	82.1	82.6	84.0	84.1	83.6	84.1	80.2
2014	93.0	83.7	81.0	84.7	82.9	80.4	83.8	82.6	83.6	82.6	82.4	69.8
2015	83.9	82.4	81.4	83.6	84.4	76.7	85.0	84.7	84.3	82.3	78.3	80.6
2016	84.0	85.3	83.5	82.7	83.9	81.1	84.0	85.4	85.8	83.0	82.4	85.3
2017	84.4	85.8	79.5	77.5	86.2	82.2	83.8	84.8	85.5	82.0	82.4	85.4
2018	NA	NA	79.8	NA	86.0	NA	85.7	87.5	NA	NA	83.3	NA

Coverage data from [13,15]. NA: Not available.

Supplementary Table 9. Reported coverage (% of total population) of ivermectin MDA for 1991-2018 in Maritime

Year	Prefecture						
	Avé	Bas-Mono	Golfe	Lacs	Vo	Yoto	Zio
1991	0	0	0	0	NA	0	0
1992	0	0	0	0	NA	0	0
1993	68.6	69.6	65.8	85.7	NA	63.7	61.4
1994	57.3	59.3	47.5	62.7	NA	59.8	70.7
1995	NA	75.6	80.4	NA	NA	NA	72.3
1996	NA	74.8	83.5	NA	NA	19.1	70.8
1997	0	0	0	0	NA	0	0
1998	NA	NA	NA	NA	NA	NA	NA
1999	NA	NA	NA	NA	NA	NA	NA
2000	65.2	72.0	NA	NA	NA	60.8	64.3
2001	62.7	74.0	75.3	85.8	NA	64.1	76.6
2002	79.6	NA	NA	76.5	NA	79.5	80.2
2003	78.3	NA	NA	74.2	NA	81.4	85.3
2004	86.7	NA	NA	86.5	NA	89.2	85.3
2005	86.1	NA	NA	87.2	NA	88.4	85.4
2006	86.7	NA	NA	88.9	NA	85.8	85.4
2007	82.8	NA	NA	82.7	NA	80.4	85.6
2008	85.5	NA	NA	84.5	NA	86.7	85.4
2009	87.6	NA	NA	88.0	NA	86.2	85.5
2010	85.9	NA	NA	87.0	NA	87.9	85.3
2011	84.1	NA	NA	82.0	NA	78.7	84.6
2012	58.0	NA	NA	82.8	NA	85.8	77.8
2013	85.7	83.6	NA	NA	NA	84.6	81.8
2014	82.8	79.1	NA	NA	NA	81.7	78.3
2015	84.0	83.0	NA	NA	NA	81.6	81.7
2016	84.1	84.9	2.4	NA	NA	80.8	81.7
2017	NA	0	NA	NA	NA	NA	NA
2018	NA	0	NA	NA	NA	NA	NA

Coverage data from [13,15]. NA: Not available.

Supplementary Text 5. Proportion of the population surveyed over time

There was a statistically significant and negative linear relationship between the proportion of the population surveyed per village and the survey year. On average, the proportion of the village population surveyed per year decreased by 0.5% (95% confidence interval (CI) = 0.4%–0.6%; linear regression p-value <0.001). For instance, over 80% of the population was examined for skin microfilariae at the beginning of the OCP, which dropped to below 70% between 2006 and 2015 (Supplementary Fig. 7), a decade during which prevalence decreased, particularly between 2006 and 2010 (Supplementary Fig. 3). The reduction in the proportion of the population tested may denote a failure to test certain high-risk population groups absent at the time of examination (e.g., fishermen and gold prospectors [50], or an increased reluctance of the population to present at the parasitological examination by skin-snip microscopy [51], as this is a mildly invasive and painful procedure. In Plateaux, the proportion of the village populations examined (ranging between 60% and 90%) did not decrease over time.

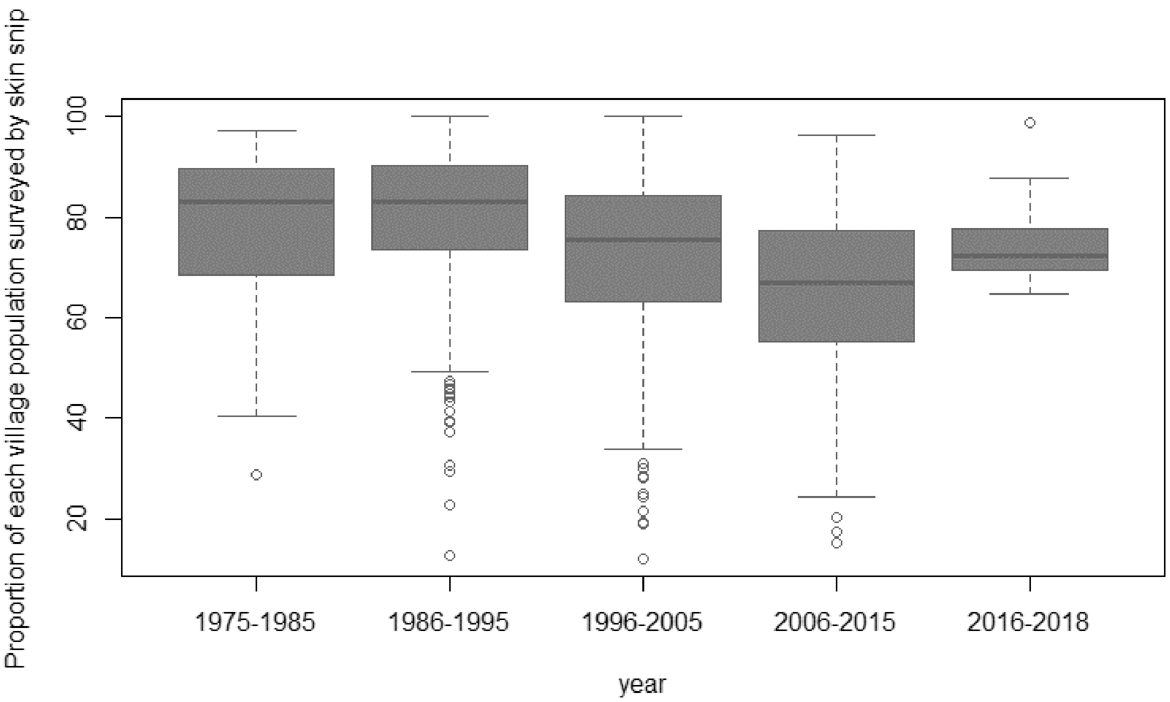

Supplementary Figure 7. Box-and-whisker plots of the proportion of the population surveyed per village according to survey years. The horizontal lines within each box represent the median proportion, with the lower and upper box edges indicating the 25th and 75th percentiles, respectively (interquartile range, IQR). Whiskers extend to 1.5 times the IQR, and the white dots indicate outliers.

Villages with recorded baseline microfilarial prevalence estimates in the OCP database

Supplementary Table 10. Villages with baseline microfilarial prevalence (BMP) estimates of *Onchocerca volvulus* by region, endemicity level and special intervention zone (SIZ) status in Togo. The model outputs and temporal infection trends of these villages are presented in Figures 2-6 of the Main Text

**Baseline endemicity level (Microfilarial prevalence)**	**Region and Figure (Main Text)**						
	**Savanes** Prefectures (*Villages*)[a] **Figure 2**		**Kara** Prefectures (*Villages*)[a] **Figure 3**	**Centrale** Prefectures (*Villages*)[a] **Figure 4**		**Plateaux** Prefectures (*Villages*)[a] **Figure 5**	**Maritime** Prefectures (*Villages*)[a] **Figure 6**
	**SIZ**	**non-SIZ**	**SIZ**	**SIZ**	**non-SIZ**	**non-SIZ**	**non-SIZ**
**Hypoendemic (<40%)**	**1:** Oti (*Fare*)	–	**2:** Assoli (*Soreda*), and Dankpen (*Natchitipi*)	–	**13:** Blitta (*Anamanie*, *Babame, Koulan-Centre, Lalamila, Nikingbe, Yambakopé, Yoloum*), Sotouboua (*Katchanke, Kaza, Ketcheboua*), Tchamba (*Assoula, Atafa II*), and Tchaoudjo (*Tchalanide*)	**16:** Agou (*Aglago-Kopé*), Akébou (*Azigo*), Anié (*Gbagbadjakou I, Tele-Kopé*), Est-Mono (*Efoufami-Yeye, Fassow, Gbomedji, Ogou-Allah*), Haho (*Alati-Pani & Medje, Kpodji-Kopé, Tcharome-Kopé*), Kloto (*Agote, Kouma-Kunda*), Moyen-Mono (*Djikame*), Ogou (*Dote-Copé*), and Wawa (*Eketo-Elavanyo*)	**6:** Avé (*Nyitakpo*), Yoto (*Akladjenou, Esse-Nadje*), and Zio (*Avedje, Dafolenyame, Kpetoe*)
**Mesoendemic (40–59.9%)**	**3:** Kpendjal (*Borgou*), and Oti (*Mogou, Panga*)	**1:** Tône (*Wokambo*)	**2:** Assoli (*Kemini/ Kemeni*), and Bassar (*Bigabo*)	–	**26:** Blitta (*Didjaré-Edjaré Kopé/Katakpui Kopé, Katakpé, Soussoukparovi, Tchanie, Toumoulmou*), Sotouboua (*Bodowda, Boungolo, Kassikide, Kedjebi-Lohou, Kpendjeria, Landa-Mono, Laoude/Somieda-Laoude, N'Djavezi/Fazao, Sessaro, Tigbada*), Tchamba (*Agoumana, Alibi 1, Djomé, Goubi, Hezoude, Mono 1, Samayi, Souroutawi*), and Tchaoudjo (*Aou-Losso, Koboyo, Salaou*)	**27:** Amou (*Ogomé Yabui*), Anié (*Flama, Kabre-Kopé, Niampopo*), Danyi (*Amouta, Wetropé, Zoubega Ouga*), Est-Mono (*Adibo, Alemondji, Atotoie, Kodjodakopé, Konta, Maroukou II, Tchankpa*), Haho (*Djemigni, Hoevime, Siyime*), Kpélé (*Kpélé-Guebakui*), Moyen-Mono (*Aglamassoe/Tététou, Pativeme*), Ogou (*Abuloukopé, Adjabouloukoukopé, Adouroukopé/Assante, Afikopé, Nangbeto-Asante*), and Wawa (*Kemedisso, Obe/Pyacope (Obetodji)*)	–

Supplementary Table 10. Continued

Baseline endemicity level (Microfilarial prevalence)	Region and Figure (Main Text)						
	**Savanes** Prefectures (*Villages*)[a] **Figure 2**		**Kara** Prefectures (*Villages*)[a] **Figure 3**	**Centrale** Prefectures (*Villages*)[a] **Figure 4**		**Plateaux** Prefectures (*Villages*)[a] **Figure 5**	**Maritime** Prefectures (*Villages*)[a] **Figure 6**
	**SIZ**	**non-SIZ**	**SIZ**	**SIZ**	**non-SIZ**	**non-SIZ**	**non-SIZ**
**Hyperendemic (60–79.9%)**	–	**2:** Kpendjal (*Koundjouaré*)and Tône (*Samomoni*)	**6:** Bassar (*Bangan, Mo-village*), Doufelgou (*Anima, Leon*), and Kozah (*Kpesside, Landa-Pozanda*)	**2:** Tchaoudjo (*Bouzalo, Sagbadai*)	**5:** Blitta (*Abossoumkopé, Agodeka, Gnama-Gnama, Kpawa, Niama-Niama*)	**25:** Agou (*Tokpo*), Akébou (*Anani/Dogokopé*), Amou (*Gnamassilé*), Anié (*Kamalo-Kopé, Konigbo*), Est-Mono (*Alabade Atsoude, Aroukakopé, Kokote, Oniakopé*), Haho (*Kokpli, Kpodji, Tetetou*), Moyen-Mono (*Diome, Gama-Ekeme, Game-Togbuihoe*), Ogou (*Alamassou, Ateoue, Atome, Fedigbe/Fétigbé, Illougba, Kpogandji, Otsanani-Adedakopé, Safou-Kopé Atiba, Tchagri*), and Wawa (*Dayes-Dodzji/Kessibo-Dzodzi/Dayi Dodji*)	**1:** Yoto (*Yoto-Kopé*)
**Holoendemic (≥80%)**	–	–	**2:** Kéran (*Titira, Tchitchira*)	–	–	–	–
**Total**	**4**	**3**	**12**	**2**	**44**	**68**	**7**

[a]Number (in bold) and name (in italics) of villages.

Supplementary Text 6. Modelled infection trends by region and Special Intervention Zone (SIZ) status for villages without recorded baseline microfilarial prevalence estimates of *Onchocerca volvulus*

The results presented here are based on trend analysis. Baseline microfilarial prevalence (BMP) estimates for these villages had not been recorded. The most likely baseline endemicity categories and intervention scenarios for these villages were inferred visually and by calculating the (median) mean squared error (MSE) between observed and simulated prevalence trends across 100 model repeats, with the best-fit scenario indicated by the smallest MSE value.

For each region and endemicity level, the best-fit scenario for villages without BMP estimates was generally consistent with the scenarios identified among villages with recorded BMP estimates. Supplementary Table 11 presents, for each region and endemicity category: (1) the scenario with the lowest MSE among villages with known BMP = ‘Best-fit for villages with BMP estimates’; (2) MSE values for villages without BMP when applying the same scenario as in (1) = ‘Best-fit for villages without BMP estimates’; (3) MSE values when the best-fit scenario for villages without BMP departed from (2) or there were no villages in (1) = ‘Alternative scenario for villages without BMP estimates’.

Supplementary Table 11. Best-fit intervention scenarios, indicated by the smallest (median) mean square error (MSE) values across 100 model repeats for villages with and without recorded baseline microfilarial prevalence (BMP), by region, Special Intervention Zone (SIZ) status and endemicity

**Region**<br><br>**(SIZ status)**	**Modelled endemicity level and prevalence trends followed by villages**	**(1) Best-fit for villages with BMP estimates[a], no. villages (Median MSE)[c]**	**(2) Best-fit for villages without BMP estimates[b], no. villages (Median MSE)[c]**	**(3) Alternative scenario for villages without BMP estimates[b], no. villages (Median MSE)[c]**
Savanes (SIZ)	Hypoendemic <100% VC efficacy, biannual MDA	NA, 0 villages	–	Reference/Enhanced, 9 villages (1–2)
	Mesoendemic <100% VC efficacy, biannual MDA	Reference, 1 village (6)	Reference, 5 villages (3)	–
	Hyperendemic <100% VC efficacy, biannual MDA	NA, 0 villages	–	Enhanced, 21 villages (38)
	Hypoendemic 100% VC efficacy, biannual MDA	Any scenario, 1 village (42)	Any scenario, 9 villages (1)	–
	Mesoendemic 100% VC efficacy, biannual MDA	Any scenario, 2 villages (2)	Any scenario, 1 village (11)	–
	Hyperendemic 100% VC efficacy, biannual MDA	NA, 0 villages	–	Enhanced, 21 villages (46)
Savanes (Non-SIZ)	Hypoendemic, annual MDA	NA, 0 villages	–	Any scenario, 2 vilages (1)
	Mesoendemic, annual MDA	Enhanced, 1 village (0)	NA, 0 villages	NA, 0 villages
	Hyperendemic 100% VC efficacy, annual MDA	Any scenario, 2 villages (1)	NA, 0 villages	NA, 0 villages
Kara (SIZ)	Hypoendemic, biannual MDA	Minimal, 2 villages (2)	Minimal, 25 villages (1)	–
	Mesoendemic, biannual MDA	Reference, 2 villages (1)	–	Minimal/ Reference/ Enhanced, 17 villages (11)
	Hyperendemic, biannual MDA	Minimal/ Reference/Enhanced, 6 villages (82)	Minimal/ Reference/Enhanced, 18 villages (93)	–
	Holoendemic, biannual MDA	Enhanced, 2 villages (175)	Enhanced, 13 villages (186)	–

[a]Figures 1-6 in Main Text; [b]Supplementary Fig. 8-14; [c]Median MSE value rounded to the nearest whole number; NA = not applicable.

Supplementary Table 11. Continued

Region<br><br>(SIZ status)	Modelled endemicity level and prevalence trends followed by villages	(1) Best-fit for villages with BMP estimates[a], no. villages (Median MSE)[c]	(2) Best-fit for villages without BMP estimates[b], no. villages (Median MSE)[c]	(3) Alternative scenario for villages without BMP estimates[b], no. villages (Median MSE)[c]
Centrale (SIZ)	Hypoendemic, biannual MDA	NA, 0 villages	–	Minimal, 1 village (1)
	Hyperendemic, biannual MDA	Minimal/ Reference/Enhanced, 2 villages (5)	Minimal/ Reference/Enhanced, 7 villages (31)	–
	Holoendemic, biannual MDA	NA, 0 villages	–	Minimal/ Reference/Enhanced, 6 villages (192)
Centrale (Non-SIZ)	Hypoendemic, annual MDA	Any scenario, 13 villages (4)	Any scenario, 8 villages (1)	–
	Mesoendemic, annual MDA	Minimal/ Reference/Enhanced, 26 villages (2)	Minimal/ Reference/Enhanced, 13 villages (6)	–
	Hyperendemic, annual MDA	Enhanced, 5 villages (13)	Enhanced, 4 villages (30)	Minimal 1 village (13)
Plateaux (Non-SIZ)	Hypoendemic annual MDA	Any scenario 12 villages (1–2)	Any scenario 14 villages (2)	–
	Mesoendemic annual MDA	Minimal/ Reference/Enhanced, 15 villages (30–31)	Minimal/ Reference/Enhanced, 3 villages (9)	–
	Hyperendemic annual MDA	Enhanced 12 villages (32)	–	Reference/Enhanced 9 villages (34)
	Hypoendemic biannual MDA	Minimal/Reference 4 villages (1)	Minimal/Reference 16 villages (12)	–
	Mesoendemic biannual MDA	Minimal/ Reference/Enhanced, 12 villages (35)	–	Minimal/ Reference, 9 villages (2)
	Hyperendemic biannual MDA	Minimal/ Reference/Enhanced, 13 villages (35)	Minimal/ Reference/Enhanced, 17 villages (56)	–
Maritime (Non SIZ)	Hypoendemic, annual MDA	Reference/Enhanced, 6 villages (1)	Reference/Enhanced, 23 villages (1)	–
	Mesoendemic, annual MDA	NA, 0 villages	–	Minimal/Reference 3 villages (1)
	Hyperendemic, annual MDA	Enhanced, 1 village (42)	Enhanced, 8 villages (1)	–

[a]Figures 1-6 in Main Text; [b]Supplementary Fig. 8-14; [c]Median MSE value rounded to the nearest whole number; NA = not applicable.

Savanes SIZ prefectures: Oti, Kpendjal and Tandjouaré

These villages were captured by hypo- to hyperendemic prevalence trends (Supplementary Fig. 8). Hyperendemic prevalence trends were observed along the Oti River Basin. Probabilities of EOT for these villages are presented in Supplementary Table 12.

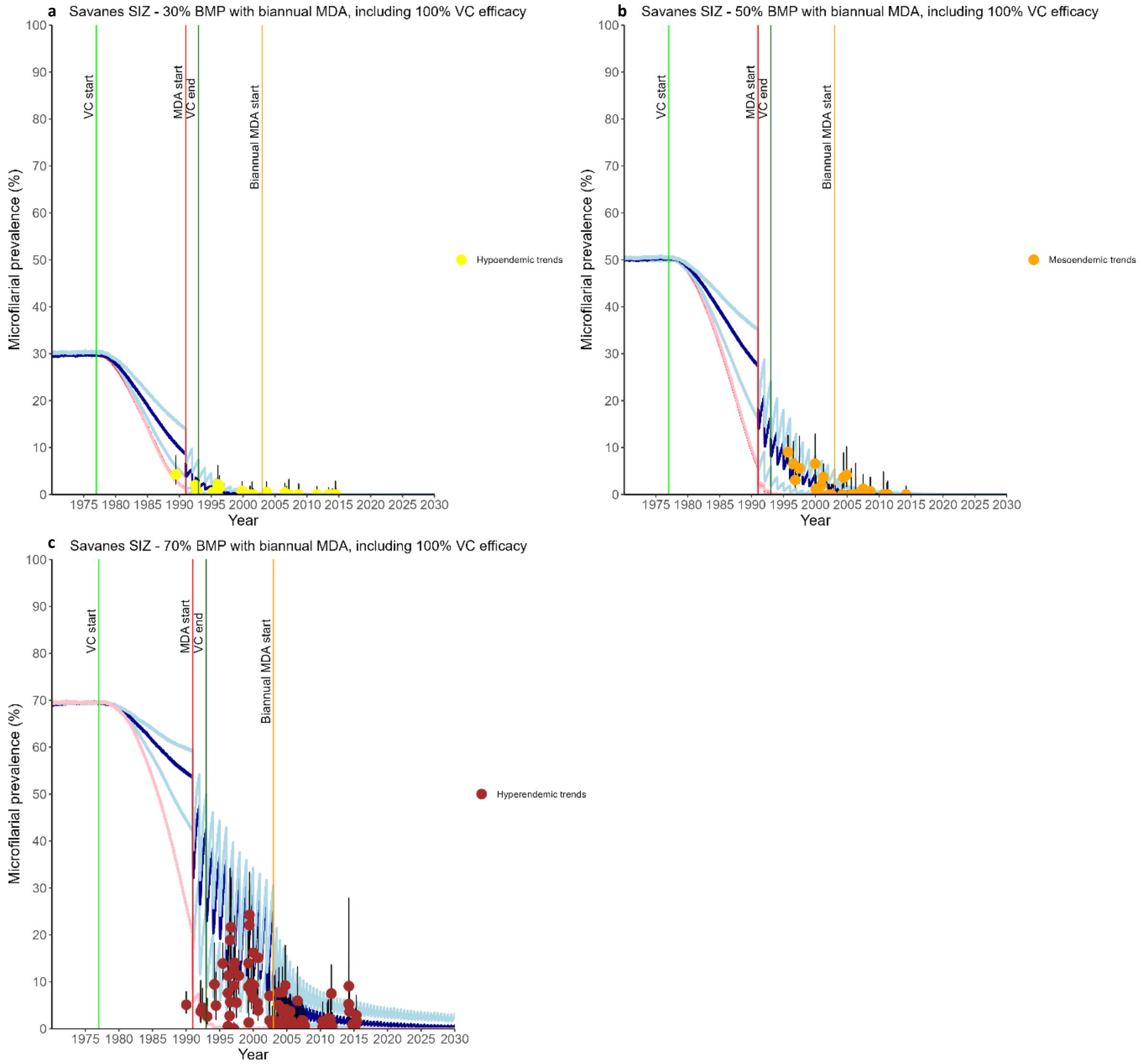

Supplementary Figure 8. *Onchocerca volvulus* microfilarial prevalence trends simulated using EPIONCHO-IBM (until 2030) and survey data for villages without recorded baseline microfilarial prevalence (BMP) estimates for Savanes Region within the Special Intervention Zone (SIZ), with vector control (VC) and ivermectin mass drug administration (MDA). (a) villages (*n*=9) following hypoendemic prevalence trends with 30% BMP and VC efficacy as in Table 2 of Main Text (blue lines) or 100% VC efficacy (red lines). (b) villages (*n*=5) following mesoendemic prevalence trends with 50% BMP and VC efficacy as in Table 2 of Main Text (blue lines) or 100% VC efficacy (red lines). (c) villages (*n*=21) following hyperendemic prevalence trends with 70% BMP and VC efficacy as in Table 2 of Main Text (blue lines) or 100% VC efficacy (red lines). The village survey data are represented by coloured circles and the error bars are the 95% (Wilson score) confidence intervals (95%CIs). Yellow circles correspond to prefectures (*villages*) following hypoendemic trends in (a): Oti (*Fareo, Gbemba-Bas, Gnangbandi, Legbando, Nagbakou, N'Kpe II, Sadori, Toutionga*), and Tandjouaré (*Dimongue*). Orange circles denote prefectures (*villages*) following mesoendemic trends in (b): Oti (*Kpinkparpak, Mantche, Nalogbandi,*

Tchitchilinga, Togou). Brown circles indicate prefectures (*villages*) following hyperendemic trends in (c): Kpendjal (*Kpintidjouaga, Moukaga, Natoundjenga, Natounkpargou, Nassiele, Pancerys, Sougtangou*), Oti (*Bonsougou, Boutchakou, Djandjatie, Kpatibori, Koukoumbou, Koulagniere, Naboli, Nambossi, Poporkou, Simbo, Tchountchonga, Tchri, Yiyingou*), and Tandjouaré (*Lokpano*). For each BMP setting and intervention scenario, the average of 100 model repeats was used to calculate the mean microfilarial prevalence dynamics (blue or red lines). Dark blue/red lines represent the reference scenario; light blue/red lines above and below dark blue/red lines indicate the minimal and enhanced scenarios, respectively. Vertical coloured lines indicate: start of VC (light green); start of annual MDA (red); end of VC (dark green); start of biannual MDA (orange). Source data and code are available [52].

Savanes non-SIZ prefectures: Tône

These villages followed hypoendemic prevalence trends (Supplementary Fig. 9). Probabilities of EOT for these villages are presented in Supplementary Table 12.

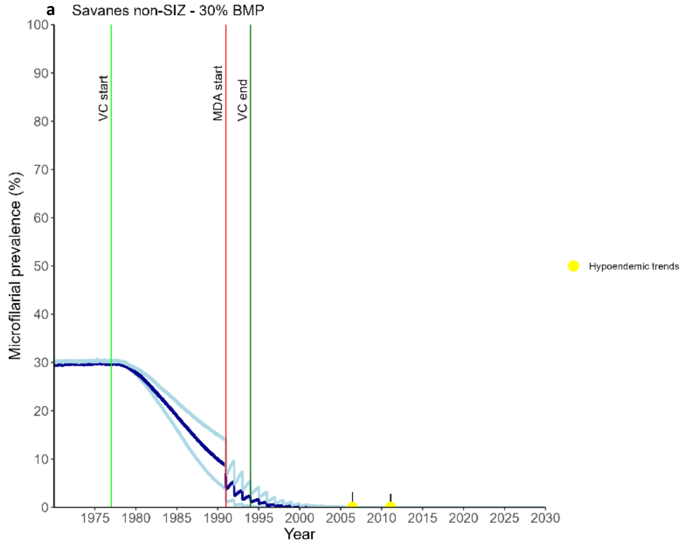

Supplementary Figure 9. *Onchocerca volvulus* microfilarial prevalence trends simulated using EPIONCHO-IBM (until 2030) and survey data for villages without recorded baseline microfilarial prevalence (BMP) estimates for Savanes Region outside the Special Intervention Zone (SIZ), with vector control (VC) and annual ivermectin mass drug administration (MDA). (a) villages (*n*=2) following hypoendemic prevalence trends with 30% BMP. The village survey data are represented by coloured circles and the error bars are the 95% (Wilson score) confidence intervals (95%CIs). Yellow circles correspond to prefectures (*villages*) following hypoendemic trends: Tône (*Lougou, Tinnogo*). For each BMP setting and intervention scenario, the average of 100 model repeats was used to calculate the mean microfilarial prevalence dynamics (blue lines). Dark blue lines represent the reference scenario; light blue lines above and below dark blue lines indicate the minimal and enhanced scenarios, respectively. Vertical coloured lines indicate: start of VC (light green); start of annual MDA (red); end of VC (dark green); start of biannual MDA (orange). Source data and code are available [52].

Kara SIZ: All prefectures

Approximately half (32 out of 75) of the villages followed hyper- to holoendemic prevalence trends (Supplementary Fig. 10). In the Oti and Mô River Basins, a few villages (two in Oti and five in Mô) exhibited patterns suggesting hyperendemic trends with "reference" and "minimal" interventions, which could hamper their achievement of EOT. Similarly, in the Kara and Kéran River Basins, several villages displayed trends indicative of hyperendemic prevalence (five in Kara and four in Kéran), following the "reference" and "minimal" intervention scenarios, or holoendemic prevalence (five in Kara and eight in Kéran), which could hinder their progress towards EOT. Probabilities of EOT for these villages are presented in Supplementary Table 13.

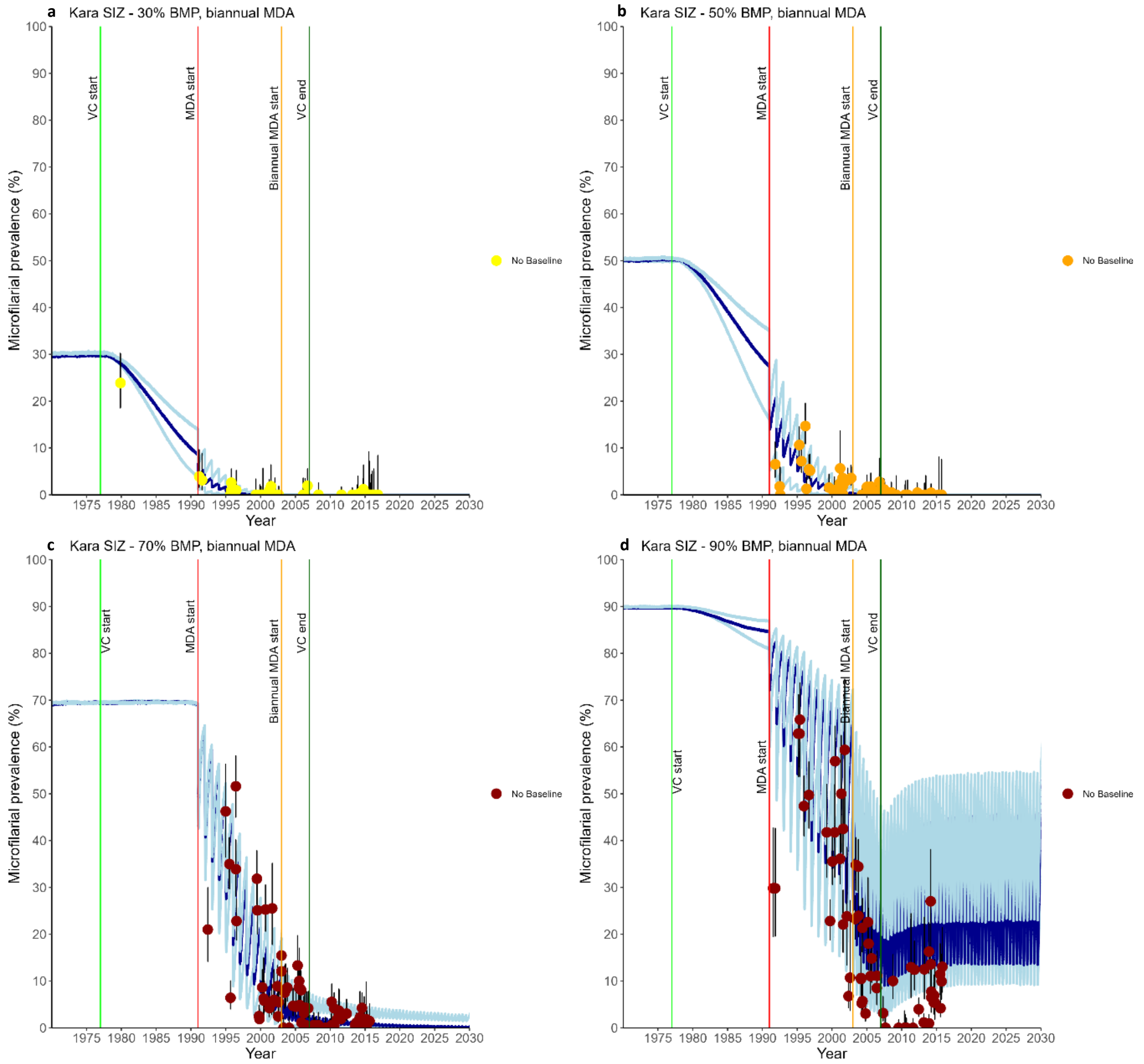

Supplementary Figure 10. ***Onchocerca volvulus*** **microfilarial prevalence trends simulated using EPIONCHO-IBM (until 2030) and survey data for villages without recorded baseline microfilarial prevalence (BMP)**

estimates for Kara Region within the Special Intervention Zone (SIZ), with vector control (VC) and ivermectin mass drug administration (MDA). (a) villages (*n*=25) following hypoendemic prevalence trends with 30% BMP; (b) villages (*n*=17) following mesoendemic prevalence trends with 50% BMP; (c) villages (*n*=18) following hyperendemic prevalence trends with 70% BMP; (d) villages (*n*=13) following holoendemic prevalence trends with 90% BMP. The village survey data are represented by coloured circles and the error bars are the 95% (Wilson score) confidence intervals (95%CIs). Yellow circles correspond to prefectures (*villages*) following hypoendemic trends in (a): Assoli (*Ouro-Gaode (Dako)*), Bassar (*Baoulinse, Bawlesi, Bougabou, Boulare*), Dankpen (*Kandjo, Kounkoumboule, Kpetab, Langa, Nandoungbale, Pesside-Ancien*), and Kozah (*Adeteyo, Agbang 2, Agbansoda, Kawa Bassar II, Koudjoukada, Kpagbazibiyo, Kpelouwai, Leziyo, Piyade, Poyo, Tchaloude, Tcholokoude, Toumboua, Toundounon*). Orange circles denote prefectures (*villages*) following mesoendemic trends in (b): Bassar (*Kassou, Tchaboua*), Binah (*Agbarada*), Dankpen (*Bowindo, Karbongou, Konfouh & Diab, Oti-village & Bidjab, Tchirkpeni (Katchamba), Tchitchikpola*), Doufelgou (*Hounde*), and Kozah (*Abouda, Bounoh, Halalomou (Filandi), Kassi (Landa), Kawa, Kpangbassibiyo, Powai*). Brown circles indicate prefectures (*villages*) following hyperendemic trends in (c): Bassar (*Dandjessi, Katcha-Konkomba, Kawa-Bassar, Kissafo, Madjatom, Saboundi*), Dankpen (*Kadjol II, Possao, Sakpone, Sekou-Bas*), Doufelgou (*Koulwere, Kpabte*), Kéran (*Hourta, Koutantagou / Koutantagou & Tapount, Wartema*), and Kozah (*Djamde Kawa, Weloude (Kpayabow), Zone Maraichere*), or those captured by holoendemic trends in (d): Bassar (*Tchakassou, Wassi*), Dankpen (*Sikan, Touguel*), Kéran (*Goulbi, Koffi-Ferme, Koutougou Solla, Kpantiiyagou, Narita/Pesside, Pesside Ferme & Wassite/Wassite, Sola, Tchitchira Ferme*), and Kozah (*Aho-Lao*). For each BMP setting and intervention scenario, the average of 100 model repeats was used to calculate the mean microfilarial prevalence dynamics (blue lines). Dark blue lines represent the reference scenario; light blue lines above and below dark blue lines indicate the minimal and enhanced scenarios, respectively. Vertical coloured lines indicate: start of VC (light green); start of annual MDA (red); start of biannual MDA (orange); end of VC (dark green). Source data and code are available [52].

Centrale SIZ prefectures: Sotouboua (including Mô)

Nearly all villages (13/14) exhibited hyper- to holoendemic trends (Mô River Basin); one village followed hypoendemic prevalence trends (Supplementary Fig. 11). Probabilities of EOT for these villages are presented in Supplementary Table 14.

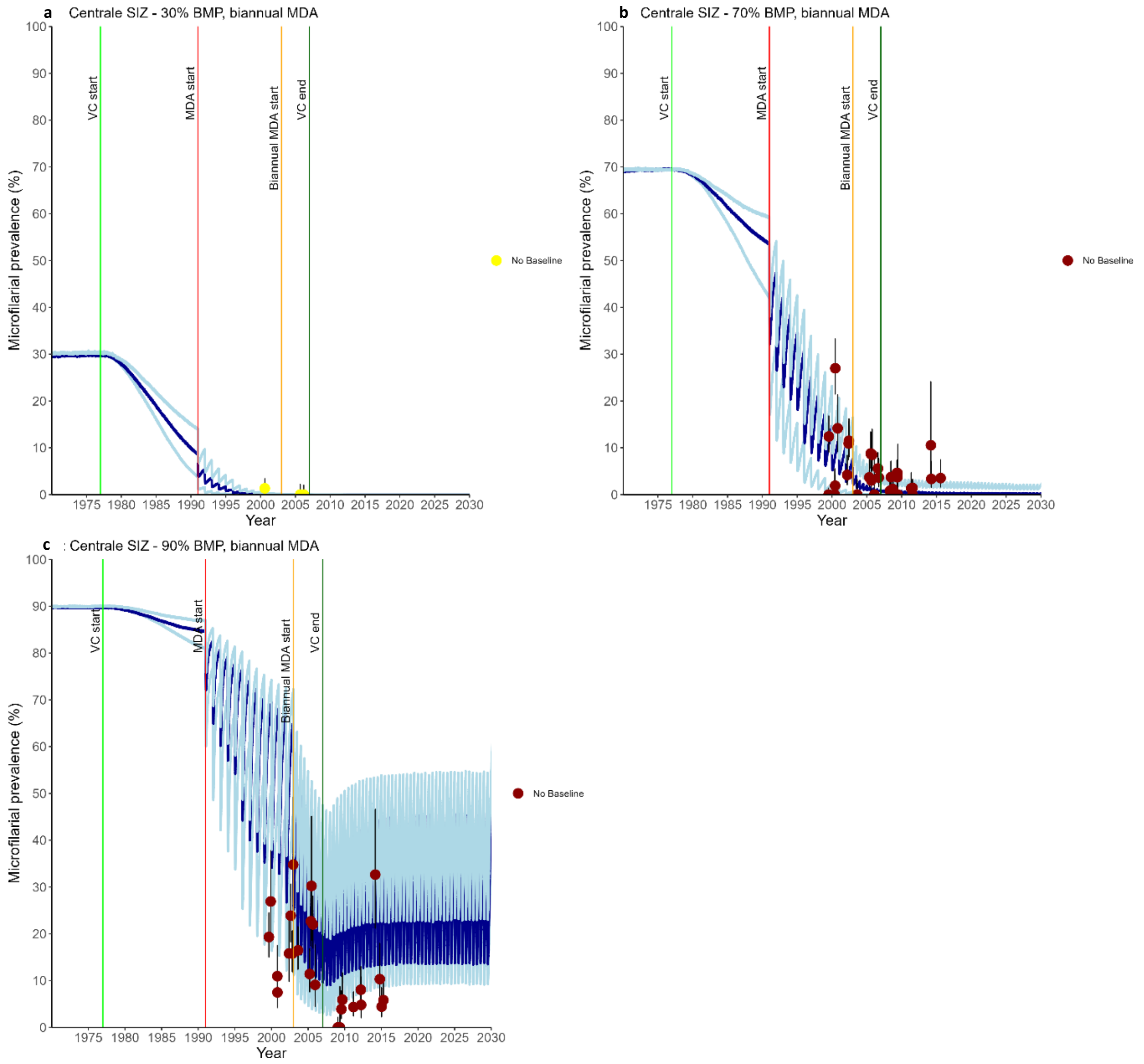

Supplementary Figure 11. *Onchocerca volvulus* microfilarial prevalence trends simulated using EPIONCHO-IBM (until 2030) and survey data for villages without recorded baseline microfilarial prevalence (BMP) estimates for Centrale Region within the Special Intervention Zone (SIZ), with vector control (VC) and ivermectin mass drug administration (MDA). (a) village (*n*=1) following hypoendemic prevalence trends with 30% BMP; (b) villages (*n*=7) following hyperendemic prevalence trends with 70% BMP; (c) villages (*n*=6) following holoendemic prevalence trends with 90% BMP. The village survey data are represented by coloured circles and the error bars are the 95% (Wilson score) confidence intervals (95%CIs). Yellow circles correspond to prefectures (*villages*) following hypoendemic trends in (a): Sotouboua (*Gnezime*). Brown circles indicate prefectures (*villages*) following hyperendemic trends in (b): Sotouboua (*Agbamassoumou, Dantchessi, Moussoukoudjou, Naboun-Koura, Tchatou Koura, Tchetchekou, Tchidao*), or those captured by holoendemic trends in (c): Sotouboua (*Assawoh-Koura, Banda, Batto, Koida, Sakpagninga, Tchakpissi*). For each BMP setting and intervention scenario, the average of 100 model repeats was used to calculate the mean microfilarial prevalence dynamics (blue lines). Dark blue lines represent the reference scenario; light blue lines above and below dark blue lines indicate the minimal and enhanced scenarios, respectively. Vertical coloured lines indicate: start of VC (light green); start of annual MDA (red); start of biannual MDA (orange); end of VC (dark green). Source data and code are available [52].

Centrale non-SIZ prefectures: Blitta, Sotouboua, Tchaoudjo and Tchamba

The villages in these prefectures, predominantly under annual MDA, followed hypo- to hyperendemic prevalence trends (Supplementary Fig. 12). Probabilities of EOT for these villages are presented in Supplementary Table 14.

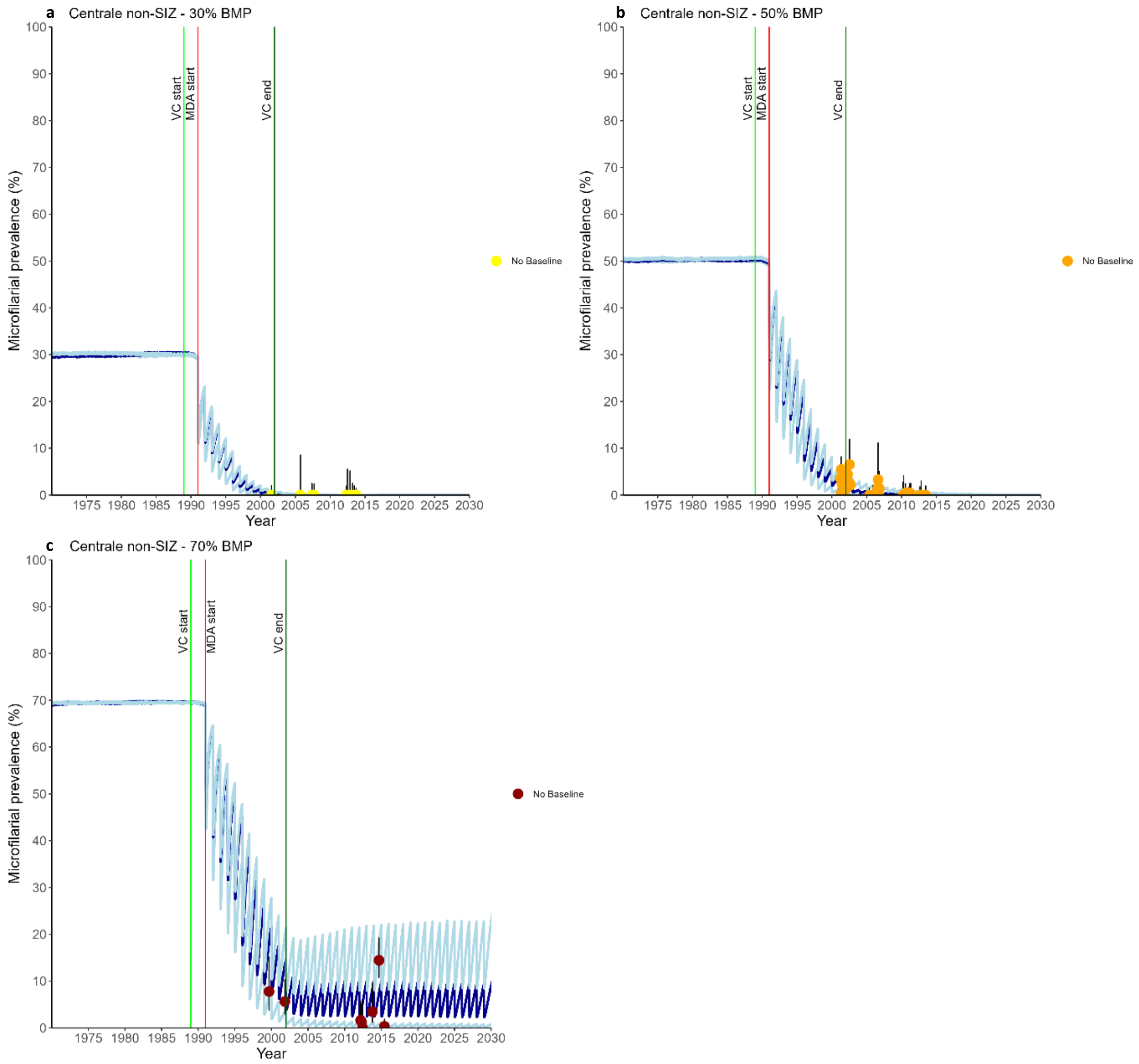

Supplementary Figure 12. *Onchocerca volvulus* microfilarial prevalence trends simulated using EPIONCHO-IBM (until 2030) and survey data for villages without recorded baseline microfilarial prevalence (BMP) estimates for Centrale Region outside the Special Intervention Zone (SIZ), with vector control (VC) and annual ivermectin mass drug administration (MDA). (a) villages (*n*=8) following hypoendemic trends with 30% BMP; (b) villages (*n*=13) following mesoendemic trends with 50% BMP; (c) villages (*n*=5) following hyperendemic trends with 70% BMP. The village survey data are represented by coloured circles and the error bars are the 95% (Wilson score) confidence intervals (95%CIs). Yellow circles correspond to prefectures (*villages*) following hypoendemic trends in (a): Blitta (*Dikpéléou/Djaoulla, Kpakparassou & Ngobo, Motchokpli, N'Kengbe, Yourourou*), Sotouboua (*Lama Were-Laouda*), and Tchaoudjo (*Baleride, Tchemberi*). Orange circles denote prefectures (*villages*) following mesoendemic trends in (b): Blitta (*Atchave, Okou-Kope, Pagala-Bouziya, Yovo-*

Kopé), Sotouboua (*Kpamboure, Kpeida, Panlao, Sada-Mono*), and Tchamba (*Akawolo, Blou-Elavagnon, Oudjomboi, Soukounde, Talaba*). Brown circles indicate prefectures (*villages*) following hyperendemic trends in (c): Blitta (*Agbandi-Mono, Yeloum Bagnan*), Sotouboua (*Katchalikadi, Takade*), and Tchamba (Ogouda & Sombo). For each BMP setting and intervention scenario, the average of 100 model repeats was used to calculate the mean microfilarial prevalence dynamics (blue lines). Dark blue lines represent the reference scenario; light blue lines above and below dark blue lines indicate the minimal and enhanced scenarios, respectively. Vertical coloured lines indicate: start of VC (light green); start of annual MDA (red); end of VC (dark green). Source data and code are available [52].

Plateaux non-SIZ: All prefectures

These villages followed hypo- to hyperendemic prevalence trends. Villages in the Agou, Akébou, Anié, Est-Mono, Kloto, Kpélé, Moyen-Mono and Wawa Prefectures received annual MDA, while those in Amou, Danyi, Haho and Ogou Prefectures switched to biannual MDA since 2014 (Supplementary Fig. 13). Probabilities of EOT for these villages are presented in Supplementary Table 15.

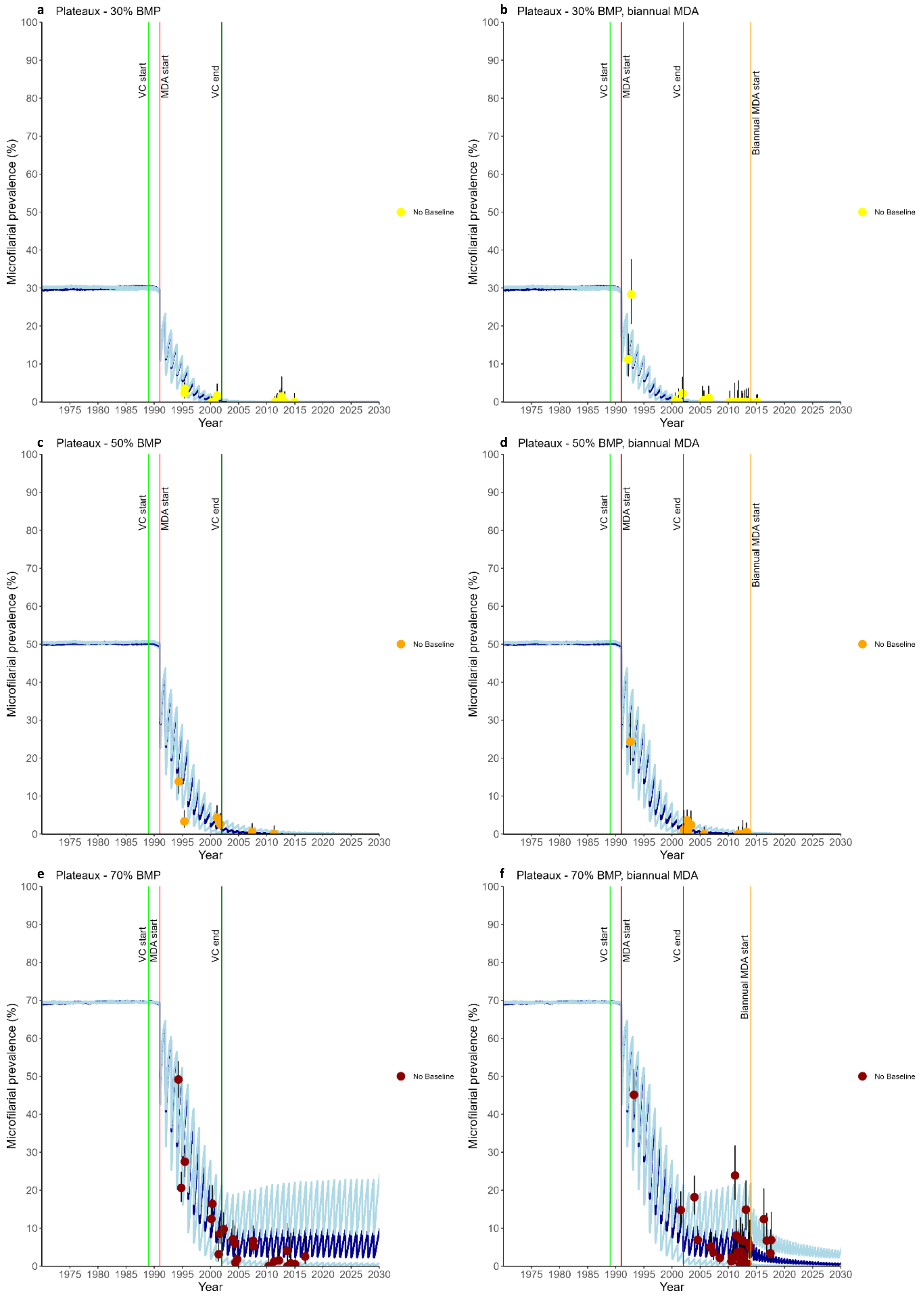

Supplementary Figure 13. ***Onchocerca volvulus*** **microfilarial prevalence trends simulated using EPIONCHO-IBM (until 2030) and survey data for villages without recorded baseline microfilarial prevalence (BMP) estimates for Plateaux Region, not included in the Special Intervention Zone (SIZ), with vector control (VC)**

and ivermectin mass drug administration (MDA). (a) villages (*n*=14) following hypoendemic prevalence trends with 30% BMP and annual MDA; (b) villages (*n*=16) following hypoendemic prevalence trends with 30% BMP and biannual MDA since 2014; (c) villages (*n*=3) following mesoendemic trends with 50% BMP and annual MDA; (d) villages (*n*=9) following mesoendemic trends with 50% BMP and biannual MDA since 2014; (e) villages (*n*=9) following hyperendemic trends with 70% BMP and annual MDA; (f) villages (*n*=17) following hyperendemic trends with 70% BMP and biannual MDA since 2014. The village survey data are represented by coloured circles and the error bars are the 95% (Wilson score) confidence intervals (95%CIs). Yellow circles correspond to prefectures (*villages*) following hypoendemic trends in (a) (annual MDA): Agou (*Agokplame, Agoudouvou, Bloudokopé, Develebe, Kpovenou, Letsoukopé, Woglokopé, Zionou*), Anié (*Alé Kopé, Gavo Kossi, Mangotigomé, Toyigbo*), and Wawa (Nougbessou Kopé, Zogbegan-Oga), or (b) (switched to biannual MDA in 2014): Amou (*Kpélé Kopé*), Haho (*Anyam-Kopé/Agnam-Copé, Gotha Adja, Gotha Kabye, Hahonou, Kome, Medze*), and Ogou (*Agborou Kopé, Akpaka, Assante, Bagaou, Ebafei-Kopé, Grokopé, Haoussa Kpédji, Kpédji, Kpété Mava*). Orange circles denote prefectures (*villages*) following mesoendemic trends in (c) (annual MDA): Agou (*Koumasse, Tome*), and Wawa (*Ahlon Dzindzi*), or (d) (switched to biannual MDA in 2014): Haho (*Amouzoukopé (Djemeni), Atalakpota, Djakpo, Houno-Kopé, Fawukpe*), and Ogou (*Amou Akpekpe, Mayaba-Kopé, Otchanari, Tchékélé*). Brown circles indicate prefectures (*villages*) following hyperendemic trends in (e) (annual MDA): Agou (*Ananivikodzi*), Anié (*Atewe-Zongo*), Kloto (*Klo-Mayondi, Kpime-Seva, Nyive*), Kpélé (*Tutu Zionou*), and Wawa (*Guin Kopé, Odomi Abra, Sukul-Kpodji*), or (f) (switched to biannual MDA in 2014): Amou (*Glelou & Omouva, Igbowou-Amou, Kpati Copé, Pidina, Tsokple*), Danyi (*Denou Bumuebi, S. Outouala*), Haho (*Amouto*), and Ogou (*Amoutchou, Atinkpassa, Glive, Hetre, Ilekohan, Moba Kopé, Tanago, Tchékélé, Toigbo*). For each BMP setting and intervention scenario, the average of 100 model repeats was used to calculate the mean microfilarial prevalence dynamics (blue lines). Dark blue lines represent the reference scenario; light blue lines above and below dark blue lines indicate the minimal and enhanced scenarios, respectively. Vertical coloured lines indicate: start of VC (light green); start of annual MDA (red); end of VC (dark green); start of biannual MDA (orange). Source data and code are available [52].

Maritime non-SIZ: All prefectures

Villages generally exhibited low endemicity, except in Yoto and possibly Avé prefectures, where some trends suggested hyperendemic levels under the enhanced intervention scenario (Supplementary Fig. 14). Probabilities of EOT for these villages are presented in Supplementary Tables 16-17.

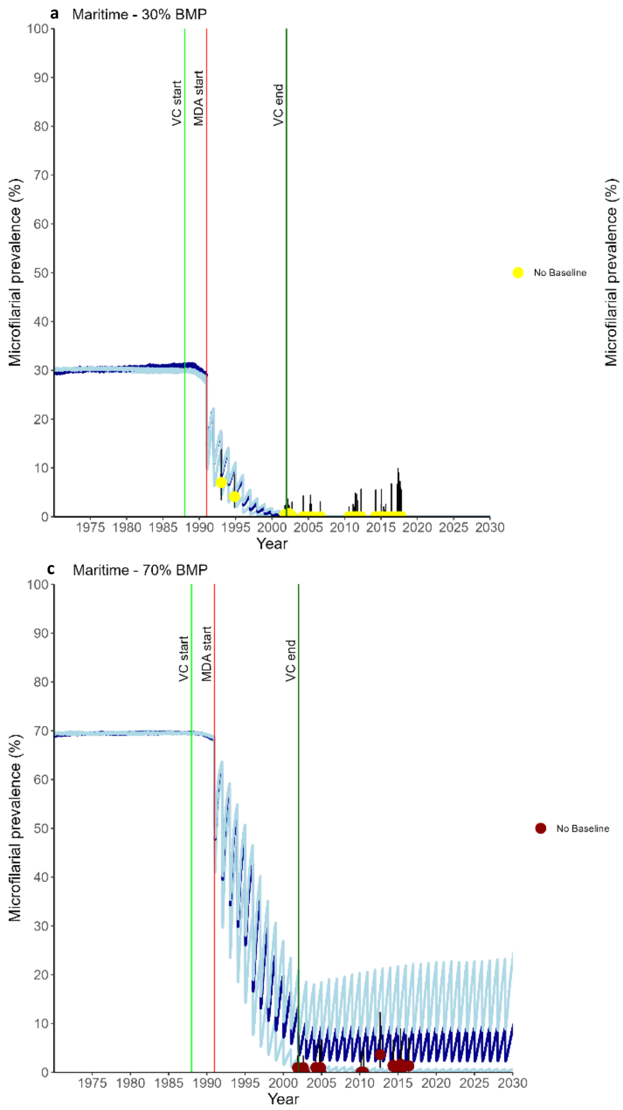

Supplementary Figure 14. *Onchocerca volvulus* microfilarial prevalence trends simulated using EPIONCHO-IBM (until 2030) and survey data for villages without recorded baseline microfilarial prevalence (BMP) estimates for Maritime Region, not included in the Special Intervention Zone (SIZ), with vector control (VC) and annual ivermectin mass drug administration (MDA). (a) villages (*n*=23) following hypoendemic prevalence trends with 30% BMP; (b) villages (*n*=3) following mesoendemic trends with 50% BMP; (c) villages (*n*=8) following hyperendemic trends with 70% BMP. The village survey data are represented by coloured circles and the error bars are the 95% (Wilson score) confidence intervals (95%CIs). Yellow circles correspond to prefectures (*villages*) following hypoendemic trends in (a): Avé (*Agotime, Alokpa*), Yoto (*Adikpe, Agoto, Atikpatafo, Avegodoe, Batoe, Drougbokopé, Haho-Kpodji, Kpeho, Moussouhoe, Noussoukopé, Sakpa-Kpensi, Tofa-Kopé, Tokpli (Zoume), Tove*), and Zio (*Agomenou, Akati Zogbe, Ake-Kondji, Dekpo, Esse Koleve, Frangadoua, Voule*). Orange circles denote prefectures (villages) following mesoendemic trends in (b): Bas-Mono (*Afomonou, Gbandidi*), and Zio (*Afokonou*). Brown circles indicate prefectures (*villages*) following hyperendemic trends in (c): Avé (*Kayido, Konta & Agbatehi*), Yoto (*Afangadji, Dzrekpon/Djrekpon, Gogokondji, Lakata-Kondji, Mawussou*) and Zio (*Togba*). For each BMP setting and intervention scenario, the average of 100 model repeats was used to calculate the mean microfilarial prevalence dynamics (blue lines). Dark blue lines represent the reference scenario; light blue lines above and below dark blue lines indicate the minimal and enhanced scenarios, respectively. Vertical coloured lines indicate: start of VC (light green); start of annual MDA (red); end of VC (dark green). Source data and code are available [52].

Projected probabilities of elimination of onchocerciasis transmission

Supplementary Table 12. Probability of elimination of onchocerciasis transmission (EOT) when simulating that ivermectin mass drug administration (MDA) stops in 2024, 2027 or 2030 per modelled (minimal, reference and enhanced) intervention scenarios in Savanes

<table>
<tr><th rowspan="3">Region and SIZ status
• Interventions</th><th rowspan="3">Modelled baseline endemicity</th><th colspan="9">Probability of elimination of transmission (EOT, %)[a]
(Number of villages following the scenario, with (BMP) or without (No BMP) recorded baseline microfilarial prevalence estimates)</th></tr>
<tr><th colspan="3">2024</th><th colspan="3">2027</th><th colspan="3">2030</th></tr>
<tr><th>Minimal</th><th>Reference</th><th>Enhanced</th><th>Minimal</th><th>Reference</th><th>Enhanced</th><th>Minimal</th><th>Reference</th><th>Enhanced</th></tr>
<tr><td rowspan="3">Savanes SIZ
• VC 1977-1993
• Annual MDA 1991-2002
• Biannual MDA from 2003</td><td>Hypoendemic</td><td colspan="9" rowspan="2">≥90
(1 BMP; 14 No BMP)</td></tr>
<tr><td>Mesoendemic</td></tr>
<tr><td>Hyperendemic</td><td><5
(3 No BMP)</td><td>20 – 59
(4 No BMP)</td><td>60 – 89
(10 No BMP)</td><td><5
(3 No BMP)</td><td>20 – 59
(4 No BMP)</td><td>60 – 89
(10 No BMP)</td><td><5
(3 No BMP)</td><td>20 – 59
(4 No BMP)</td><td>60 – 89
(10 No BMP)</td></tr>
<tr><td rowspan="3">Savanes SIZ
• VC 1977-1993 (100%)[a]
• Annual MDA 1991-2002
• Biannual MDA from 2003</td><td>Hypoendemic</td><td colspan="9" rowspan="2">≥90
(3 BMP; 14 No BMP)</td></tr>
<tr><td>Mesoendemic</td></tr>
<tr><td>Hyperendemic</td><td>60 – 89
(3 No BMP)</td><td colspan="2">≥90
(1 No BMP)</td><td>60 – 89
(3 No BMP)</td><td colspan="2">≥90
(1 No BMP)</td><td>60 – 89
(3 No BMP)</td><td colspan="2">≥90
(1 No BMP)</td></tr>
<tr><td rowspan="3">Savanes non-SIZ
• VC 1977-1993
• Annual MDA from 1991</td><td>Hypoendemic</td><td colspan="9">≥90
(2 No BMP; 3 BMP[b])</td></tr>
<tr><td>Mesoendemic</td><td>60 – 89
(1 BMP[b])</td><td colspan="2">≥90
(1 BMP)</td><td>60 – 89
(1 BMP[b])</td><td colspan="2">≥90
(1 BMP)</td><td>60 – 89
(1 BMP[b])</td><td colspan="2">≥90
(1 BMP)</td></tr>
<tr><td>Hyperendemic</td><td colspan="9"><5
(0)</td></tr>
</table>

Supplementary Table 12. Continued

<table>
<tr><th rowspan="3">Region and SIZ status

• Interventions</th><th rowspan="3">Modelled baseline endemicity</th><th colspan="9">Probability of elimination of transmission (EOT, %)[a]
(Number of villages following the scenario, with (BMP) or without (No BMP)
recorded baseline microfilarial prevalence estimates)</th></tr>
<tr><th colspan="3">2024</th><th colspan="3">2027</th><th colspan="3">2030</th></tr>
<tr><th>Minimal</th><th>Reference</th><th>Enhanced</th><th>Minimal</th><th>Reference</th><th>Enhanced</th><th>Minimal</th><th>Reference</th><th>Enhanced</th></tr>
<tr><td rowspan="3">Savanes non-SIZ

• VC 1977-1993 (100%)[a]
• Annual MDA from 1991</td><td>Hypoendemic</td><td colspan="9" rowspan="2">≥90
(0)</td></tr>
<tr><td>Mesoendemic</td></tr>
<tr><td>Hyperendemic</td><td>20 – 59
(2 No BMP;
1 BMP[b])</td><td>60 – 89
(0)</td><td>≥90
(0)</td><td>20 – 59
(2 BMP;
1 BMP[b])</td><td>60 – 89
(0)</td><td>≥90
(0)</td><td>20 – 59
(2 BMP;
1 BMP[b])</td><td>60 – 89
(0)</td><td>≥90
(0)</td></tr>
</table>

[a]Probability of elimination of onchocerciasis transmission simulated as the proportion (%) of 100 model runs for each baseline endemicity level and intervention scenario with 0% microfilarial prevalence 50 years after stopping ivermectin MDA.

[b]Village from preparatory surveys prior to the commencement of the OCP [1,7] (not in OCP database).

BMP, baseline microfilarial prevalence; EOT, elimination of transmission; MDA, mass drug administration with ivermectin; SIZ, special intervention zone; VC, vector control by aerial larviciding of breeding sites. [a]100% vector control efficacy [46].

Supplementary Table 13. Probability of elimination of onchocerciasis transmission (EOT) when simulating that ivermectin mass drug administration (MDA) stops in 2024, 2027 or 2030 per modelled (minimal, reference and enhanced) intervention scenarios in Kara

Region and SIZ status<br>• Interventions	Modelled baseline endemicity	Probability of elimination of transmission (EOT, %)[a]<br>(Number of villages following the scenario, with (BMP) or without (No BMP) recorded baseline microfilarial prevalence estimates)								
		2024			2027			2030		
		Minimal	Reference	Enhanced	Minimal	Reference	Enhanced	Minimal	Reference	Enhanced
Kara SIZ<br>• VC 1977-2007<br>• Annual MDA 1991-2002<br>• Biannual MDA from 2003	Hypoendemic	≥90<br>(4 BMP; 2 BMP[b]; 42 No BMP)								
	Mesoendemic									
	Hyperendemic	5 – 19<br>(2 BMP; 11 No BMP)	20 – 59<br>(2 BMP; 1 BMP[b]; 5 No BMP)	60 – 89<br>(2 BMP; 2 No BMP)	5 – 19<br>(2 BMP; 11 No BMP)	60 – 89<br>(2 BMP; 1 BMP[b]; 5 No BMP)	≥90<br>(2 BMP; 2 No BMP)	20 – 59<br>(2 BMP; 11 No BMP)	60 – 89<br>(2 BMP; 1 BMP[b]; 5 No BMP)	≥90<br>(2 BMP; 2 No BMP)
	Holoendemic	<5%<br>(2 BMP; 13 No BMP)								

[a]Probability of elimination of onchocerciasis transmission simulated as the proportion (%) of 100 model runs for each baseline endemicity level and intervention scenario with 0% microfilarial prevalence 50 years after stopping ivermectin MDA.

[b]Village from preparatory surveys prior to the commencement of the OCP [1,7] (not in OCP database).

BMP, baseline microfilarial prevalence; EOT, elimination of transmission; MDA, mass drug administration with ivermectin; SIZ, special intervention zone; VC, vector control by aerial larviciding of breeding sites.

Supplementary Table 14. Probability of elimination of onchocerciasis transmission (EOT) when simulating that ivermectin mass drug administration (MDA) stops in 2024, 2027 or 2030 per modelled (minimal, reference and enhanced) intervention scenarios in Centrale

Region and SIZ status<br>• Interventions	Modelled baseline endemicity	Probability of elimination of transmission (EOT, %)[a]<br>(Number of villages following the scenario, with (BMP) or without (No BMP) recorded baseline microfilarial prevalence estimates)								
		2024			2027			2030		
		Minimal	Reference	Enhanced	Minimal	Reference	Enhanced	Minimal	Reference	Enhanced
Centrale SIZ<br>• VC 1977-2007<br>• Annual MDA 1991-2002<br>• Biannual MDA from 2003	Hypoendemic	≥90<br>(1 No BMP)								
	Mesoendemic									
	Hyperendemic	5 – 19<br>(1 BMP; 3 No BMP)	60 – 89<br>(3 No BMP)	60 – 89<br>(1 BMP; 1 No BMP)	5 – 19<br>(1 BMP; 3 No BMP)	60 – 89<br>(3 No BMP)	≥90<br>(1 BMP; 1 No BMP)	20 – 59<br>(1 BMP; 3 No BMP)	60 – 89<br>(3 No BMP)	≥90<br>(1 BMP; 1 No BMP)
	Holoendemic	<5<br>(6 No BMP)								
Centrale non-SIZ<br>• VC 1989-2002<br>• Annual MDA from 1991	Hypoendemic	≥90<br>(13 BMP; 8 No BMP)								
	Mesoendemic	60 – 89<br>(8 BMP; 11 No BMP)	≥90<br>(18 BMP; 2 No BMP)		60 – 89<br>(8 BMP; 11 No BMP)	≥90<br>(18 BMP; 2 No BMP)		60 – 89<br>(8 BMP; 11 No BMP)	≥90<br>(18 BMP; 2 No BMP)	
	Hyperendemic	<5<br>(2 No BMP)	60 – 89<br>(5 BMP; 3 No BMP)		<5<br>(2 No BMP)	60 – 89<br>(5 BMP; 3 No BMP)		<5<br>(2 No BMP)	60 – 89<br>(5 BMP; 3 No BMP)	

[a]Probability of elimination of onchocerciasis transmission simulated as the proportion (%) of 100 model runs for each baseline endemicity level and intervention scenario with 0% microfilarial prevalence 50 years after stopping ivermectin MDA.

BMP, baseline microfilarial prevalence; EOT, elimination of transmission; MDA, mass drug administration with ivermectin; SIZ, special intervention zone; VC, vector control by aerial larviciding of breeding sites.

Supplementary Table 15. Probability of elimination of onchocerciasis transmission (EOT) when simulating that ivermectin mass drug administration (MDA) stops in 2024, 2027 or 2030 per modelled (minimal, reference and enhanced) intervention scenario in Plateaux

Region and SIZ status<br>• Interventions	Modelled baseline endemicity	Probability of elimination of transmission (EOT, %)[a] (Number of villages following the scenario, with (BMP) or without (No BMP) recorded baseline microfilarial prevalence estimates)								
		2024			2027			2030		
		Minimal	Reference	Enhanced	Minimal	Reference	Enhanced	Minimal	Reference	Enhanced
Plateaux non-SIZ<br>• VC 1989-2002<br>• Annual MDA from 1991	Hypoendemic	≥90 (12 BMP; 14 No BMP)								
	Mesoendemic	60 – 89 (3 BMP; 2 No BMP)	≥90 (12 BMP; 1 No BMP)		60 – 89 (3 BMP; 2 No BMP)	≥90 (12 BMP; 1 No BMP)		60 – 89 (3 BMP; 2 No BMP)	≥90 (12 BMP; 1 No BMP)	
	Hyperendemic	<5 (0)	<5 (3 No BMP)	60 – 89 (12 BMP; 6 No BMP)	<5 (0)	<5 (3 No BMP)	60 – 89 (12 BMP; 6 No BMP)	<5 (0)	<5 (3 No BMP)	60 – 89 (12 BMP; 6 No BMP)
Plateaux non-SIZ<br>• VC 1989-2002<br>• Annual MDA 1991-2013<br>• Biannual MDA from 2014	Hypoendemic	≥90 (16 BMP; 25 No BMP)								
	Mesoendemic									
	Hyperendemic	<5 (3 No BMP)	5 – 19 (2 BMP; 2 No BMP)	60 – 89 (11 BMP; 12 No BMP)	<5 (3 No BMP)	5 – 19 (2 BMP; 2 No BMP)	60 – 89 (11 BMP; 12 No BMP)	<5 (3 No BMP)	20 – 59 (2 BMP; 2 No BMP)	≥90 (11 BMP; 12 No BMP)

[a]Probability of elimination of onchocerciasis transmission simulated as the proportion (%) of 100 model runs for each baseline endemicity level and intervention scenario with 0% microfilarial prevalence 50 years after stopping ivermectin MDA.

BMP, baseline microfilarial prevalence; EOT, elimination of transmission; MDA, mass drug administration with ivermectin; SIZ, special intervention zone; VC, vector control by aerial larviciding of breeding sites.

Supplementary Table 16. Probability of elimination of onchocerciasis transmission (EOT) when simulating that ivermectin mass drug administration (MDA) stops in 2014 or 2020 per modelled (minimal, reference and enhanced) intervention scenario in Maritime

<table>
<tr><th rowspan="3">Region and SIZ status

Interventions</th><th rowspan="3">Modelled baseline endemicity</th><th colspan="6">Probability of elimination of transmission (EOT, %)[a]
(Number of villages following the scenario, with (BMP) or without (No BMP) recorded baseline microfilarial prevalence estimates)</th></tr>
<tr><th colspan="3">2014</th><th colspan="3">2020</th></tr>
<tr><th>Minimal</th><th>Reference</th><th>Enhanced</th><th>Minimal</th><th>Reference</th><th>Enhanced</th></tr>
<tr><td rowspan="3">Maritime non-SIZ

• VC 1989-2002
• Annual MDA from 1991</td><td>Hypoendemic</td><td colspan="6">≥90
(6 BMP; 23 No BMP)</td></tr>
<tr><td>Mesoendemic</td><td>20 – 59
(0)</td><td colspan="2">≥90
(0)</td><td>60 – 89
(3 No BMP)</td><td colspan="2">≥90
(0)</td></tr>
<tr><td>Hyperendemic</td><td colspan="2"><5
(0)</td><td>20 – 59
(0)</td><td colspan="2"><5
(0)</td><td>20 – 59
(6 No BMP)</td></tr>
</table>

[a]Probability of elimination of onchocerciasis transmission simulated as the proportion (%) of 100 model runs for each baseline endemicity level and intervention scenario with 0% microfilarial prevalence 50 years after stopping ivermectin MDA.

BMP, baseline microfilarial prevalence; EOT, elimination of transmission; MDA, mass drug administration with ivermectin; SIZ, special intervention zone; VC, vector control by aerial larviciding of breeding sites.

Supplementary Table 17. Probability of elimination of onchocerciasis transmission (EOT) when simulating that ivermectin mass drug administration (MDA) stops in 2024, 2027 or 2030 per modelled (minimal, reference and enhanced) intervention scenario in Maritime

**Region and SIZ status**<br><br>**Interventions**	**Modelled baseline endemicity**	**Probability of elimination of transmission (EOT, %)[a]**<br>**(Number of villages following the scenario, with (BMP) or without (No BMP) recorded baseline microfilarial prevalence estimates)**								
		**2024**			**2027**			**2030**		
		**Minimal**	**Reference**	**Enhanced**	**Minimal**	**Reference**	**Enhanced**	**Minimal**	**Reference**	**Enhanced**
Maritime non-SIZ	Hypoendemic	≥90<br>(0)								
• VC 1989-2002<br>• Annual MDA from 1991	Mesoendemic	60 – 89<br>(0)		≥90<br>(0)	60 – 89<br>(0)		≥90<br>(0)	≥90<br>(0)		
	Hyperendemic	<5<br>(0)		60 – 89<br>(1 BMP;<br>2 No BMP)	<5<br>(0)		60 – 89<br>(1 BMP;<br>2 No BMP)	<5<br>(0)		60 – 89<br>(1 BMP;<br>2 No BMP)

[a]Probability of elimination of onchocerciasis transmission simulated as the proportion (%) of 100 model runs for each baseline endemicity level and intervention scenario with 0% microfilarial prevalence 50 years after stopping ivermectin MDA.

BMP, baseline microfilarial prevalence; EOT, elimination of transmission; MDA, mass drug administration with ivermectin; SIZ, special intervention zone; VC, vector control by aerial larviciding of breeding sites.

Supplementary Text 7. Villages projected not to reach elimination of onchocerciasis transmission (EOT) if ivermectin MDA stops in 2027, per region and special intervention zone (SIZ) status

Supplementary Tables 18-23 (villages with recorded BMP estimates) and 24-29 (villages without recorded BMP estimates) provide details, by region, prefecture and river basin, of villages for which EPIONCHO-IBM projects that elimination of onchocerciasis transmission (EOT) may not be achieved if ivermectin MDA stops in 2027 (i.e., with predicted EOT probability <90%). (For calculation of EOT probabilities see Main Text and Supplementary Tables 12-17.) These villages could be prioritised for focused pre-stop or stop-MDA surveys to evaluate their progress and obtain a more complete assessment of the epidemiological situation in the region towards the 2030 elimination goals. For example, in Plateaux non-SIZ, the formerly hyperendemic village of Kokote (Mono River Basin) had a sharp prevalence decline from 76% in 1977 to 56% in 1990 and to 2% in 2000. A survey in 2007, and a small survey (51 individuals) in 2014, detected no positive cases. However, a larger survey (253 individuals), also in 2014, identified one positive case, suggesting residual transmission. This finding is consistent with model outputs (Figure 5E of the Main Text), with an EOT probability of 60–89% (Supplementary Table 22).

7.1. Villages with recorded baseline microfilarial prevalence estimates (with BMP)

Supplementary Table 18. Villages in Savanes not included in the special intervention zone (non-SIZ)

Village	Prefecture	**Modelled prevalence trends followed by villages** (intervention scenario)	Latest parasitological survey		EOT probability (%) if MDA stops in 2027
			Year	**Microfilarial prevalence (%)** (95% CI)	
**White Volta/Oti River Basin**					
Samomoni[a,b]	Tône	Hyperendemic with 100% vector control (minimal)	2015	0.8 (0.1–5.2)	20–59
Koundjouaré[b]	Kpendjal	Hyperendemic with 100% vector control (minimal)	2001	0.1 (0.0–0.3)	20–59

[a]Recorded in a baseline survey prior to the start of the Onchocerciasis Control Programme in West Africa [1,7].
[b]For these two villages the model indicates an EOT probability of 20-59%; surveys conducted in the 2000s showed residual prevalence, which along with model simulations, suggest that prevalence may have been sustained over time if EOT was not achieved by the mid-1990s, when VC stopped.

Supplementary Table 19. Villages in Kara included in the special intervention zone (SIZ)

Village	Prefecture	Modelled prevalence trends followed by villages (intervention scenario)	Latest parasitological survey		EOT probability (%) if MDA stops in 2027
			Year	Microfilarial prevalence (%) (95% CI)	
**Kara River Basin**					
Kpesside	Kozah	Hyperendemic (minimal)	2014	1.4 (0.5–3.9)	5–19
Leon	Doufelgou	Hyperendemic (reference)	2011	0.0 (0.0–1.4)	60–89
**Kéran River Basin**					
Unknown[a]	Binah or Bimah	Hyperendemic (reference)	1970	>70%	60–89
Tchitchira[b]	Kéran	Holoendemic (enhanced)	2015	8.2 (5.1–12.9)	<5
Titira[c]	Kéran	Holoendemic (enhanced)	2006	1.6 (0.5–4.6)	<5
**Mô River Basin**					
Bangan[b]	Bassar	Hyperendemic (reference)	2011	2.5 (1.2–5.4)	60–89
Mô-village or Mo-village	Bassar	Hyperendemic (minimal)	2015	7.0 (4.2–11.7)	5–19

[a]Recorded in a baseline survey prior to the start of the Onchocerciasis Control Programme in West Africa [1,7].
[b]In 2015, prevalence of *O. volvulus* infection in *Simulium damnosum* sensu lato was 0.1% (95% CI: 0.03-0.5%) in Bangan and 1.0% (95% CI: 0.9-2.1%) in Tchitchira [13].
[c]In Tititra, annual biting rates decreased from 25,000 bites/person/year before control to 10,000-15,000 [45].

Supplementary Table 20. Villages in Centrale included in the special intervention zone (SIZ)

Village	Prefecture	Modelled prevalence trends followed by villages (intervention scenario)	Latest parasitological survey		EOT probability (%) if MDA stops in 2027
			Year	Microfilarial prevalence (%) (95% CI)	
**Mô River Basin**					
Bouzalo[a,b]	Tchaoudjo	Hyperendemic (reference)	1993	7.5 (5.3–10.5)	60–89
Sagbadai	Tchaoudjo	Hyperendemic (minimal)	2007	1.8 (0.3–9.5)	5–19

[a]In 2015, the prevalence of *O. volvulus* infection in in *Simulium damnosum* sensu lato was 0.5% (95% CI: 0.2-1.3%%) in Bouzalo [13]. In 2018-2019, the prevalence was 0.6% [29] (calculated with the methodology presented in Katholi et al. [53]). [b]In Bouzalo, annual biting rates decreased from over 40,000 bites/person/year before vector control to 30,000. The vector control extension during the Southern Extension of the OCP further decreased this to 10,000-15,000. In Tititra, annual biting rates decreased from 25,000 bites/person/year before vector control to 10,000-15,000 [45].

Supplementary Table 21. Villages in Centrale not included in the special intervention zone (non-SIZ)

Village	Prefecture	Modelled prevalence trends followed by villages (intervention scenario)	Latest parasitological survey		EOT probability (%) if MDA stops in 2027
			Year	Microfilarial prevalence (%) (95% CI)	
**Anié River Basin (Mono)**					
Agodeka[a]	Blitta	Hyperendemic (enhanced)	2012	0.0 (0.0–2.2)	60–89
Didjaré-Edjaré Kopé/Katakpui Kopé (Pagala)	Blitta	Mesoendemic (minimal)	2014	0.3 (0.1–1.9)	60–89
Gnama-Gnama	Blitta	Hyperendemic (enhanced)	2012	0.0 (0.0–5.4)	60–89
Kpawa (Pagala)	Blitta	Hyperendemic (enhanced)	2013	0.0 (0.0–1.3)	60–89
Niama-Niama (Pagala)	Blitta	Hyperendemic (enhanced)	2006	2.6 (0.9–7.5)	60–89
N'Djavezi/Fazao	Sotouboua	Mesoendemic (minimal)	2014	0.0 (0.0–3.8)	60–89
Tigbada	Sotouboua	Mesoendemic (minimal)	2013	1.0 (0.3–3.5)	60–89
**Asukawkaw River Basin**					
Abossoumkopé[a]	Blitta	Hyperendemic (enhanced)	2013	0.0 (0.0–6.1)	60–89
**Landa-Mono River Basin (Mono)**					
Bodowda	Sotouboua	Mesoendemic (minimal)	2013	0.0 (0.0–4.5)	60–89
Laoude/Somieda-Laoude	Sotouboua	Mesoendemic (minimal)	2013	0.6 (0.1–3.3)	60–89
Sessaro	Sotouboua	Mesoendemic (minimal)	2014	0.3 (0.1–1.9)	60–89
Souroutawi	Tchamba	Mesoendemic (minimal)	2013	0.3 (0.1–1.7)	60–89
**Mono River Basin (Aou)**					
Aou-Losso	Tchaoudjo	Mesoendemic (minimal)	2013	0.6 (0.1–3.1)	60–89

[a]Villages with the latest two (Agodeka) or three (Abossoumkopé) surveys with 0% prevalence [15].

Supplementary Table 22. Villages in Plateaux not included in the special intervention zone (non-SIZ)

Village	Prefecture	Modelled prevalence trends followed by villages (intervention scenario)	Latest parasitological survey		EOT probability (%) if MDA stops in 2027
			Year	Microfilarial prevalence (%) (95% CI)	
**Amou River Basin (Mono)**					
Otsanani-Adedakope	Ogou	Hyperendemic (enhanced) under biannual CDTI since 2014	2012	1.2 (0.3–3.5)	60–89
**Anié River Basin (Mono)**					
Alamassou	Ogou	Hyperendemic (enhanced) under biannual CDTI since 2014	2012	0.0 (0.0–1.5)	60–89
Anani/Dogo Kopé	Akébou	Hyperendemic (enhanced) under annual CDTI	2012	1.0 (0.4–3.0)	60–89
Gnamassilé	Amou	Hyperendemic (enhanced) under biannual CDTI since 2014	2014	0.0 (0.0–2.7)	60–89
Illougba	Ogou	Hyperendemic (enhanced) under biannual CDTI since 2014	2007	0.0 (0.0–1.9)	60–89
Kamalo-Kopé[a]	Anié	Hyperendemic (enhanced) under annual CDTI	2007	0.0 (0.0–2.9)	60–89
Konigbo	Anié	Hyperendemic (enhanced) under annual CDTI	2012	0.0 (0.0–1.1)	60–89
**Wawa/Asukawkaw River Basin**					
Kemedisso	Wawa	Mesoendemic (minimal) under annual CDTI	2007	0.0 (0.0–4.5)	60–89
**Kra River Basin (Mono)**					
Kokpli	Haho	Hyperendemic (enhanced) under biannual CDTI since 2014	2006	1.0 (0.3–3.4)	60–89
**Mono River Basin**					
Aglamassoe/ Tététou	Moyen-Mono	Mesoendemic (minimal) under annual CDTI	2014	0.0 (0.0–10.7)	60–89
Alabade Atsoude	Est-Mono	Hyperendemic (enhanced) under annual CDTI	2013	0.0 (0.0–1.9)	60–89

Supplementary Table 22. Continued

Village	Prefecture	Modelled prevalence trends followed by villages (intervention scenario)	Latest parasitological survey		EOT probability (%) if MDA stops in 2027
			Year	Microfilarial prevalence (%) (95% CI)	
**Mono River Basin (continued)**					
Atome	Ogou	Hyperendemic (enhanced) under biannual CDTI since 2014	2015	0.4 (0.1–2.3)	60–89
Aroukakopé (Amou-Oblo)	Est-Mono	Hyperendemic (enhanced) under annual CDTI	2011	0.0 (0.0–2.5)	60–89
Diome (Tététou)	Moyen-Mono	Hyperendemic (enhanced) under annual CDTI	1977	77.0 (71.8–81.4)	60–89
Fedigbe or Fétigbé	Ogou	Hyperendemic (enhanced) under biannual CDTI since 2014	2007	0.0 (0.0–1.8)	60–89
Game-Ekeme	Moyen-Mono	Hyperendemic (enhanced) under annual CDTI	2011	0.0 (0.0–5.8)	60–89
Game-Togbuihoe	Moyen-Mono	Hyperendemic (enhanced) under annual CDTI	2011	0.0 (0.0–4.8)	60–89
Kokote[b]	Est-Mono	Hyperendemic (enhanced) under annual CDTI	2014	0.4 (0.1–2.2)	60–89
Kpodji (Tététou)	Haho	Hyperendemic (reference) under biannual CDTI since 2014	2014	17.5 (9.8–29.4)	5–19
Kpogandi[a]	Ogou	Hyperendemic (enhanced) under biannual CDTI since 2014	2013	0.0 (0.0–2.9)	60–89
Onia-Kopé	Est-Mono	Hyperendemic (enhanced) under annual CDTI	1977	75.2 (67.5–81.6)	60–89
Safou-Kopé Atiba (Amou-Oblo)	Ogou	Hyperendemic (reference) under biannual CDTI since 2014	2014	4.1 (1.4–11.4)	5–19
Siyime (Tététou)	Haho	Mesoendemic/Hyper-endemic (reference) under biannual CDTI since 2014	2015	2.9 (1.2–6.6)	5–19

Supplementary Table 22. Continued

Village	Prefecture	Modelled prevalence trends followed by villages (intervention scenario)	Latest parasitological survey		EOT probability (%) if MDA stops in 2027
			Year	Microfilarial prevalence (%) (95% CI)	
**Mono River Basin (continued)**					
Tchagri	Ogou	Hyperendemic (enhanced) under biannual CDTI since 2014	2012	0.0 (0.0–1.0)	60–89
Tététou or Tetetou	Haho	Hyperendemic (enhanced) under biannual CDTI since 2014	2002	0.0 (0.0–1.6)	60–89
**Ogou River Basin (Mono)**					
Ateoue	Ogou	Hyperendemic (enhanced) under biannual CDTI since 2014	2000	4.1 (2.3–7.2)	60–89
**Wawa River Basin (Gban-Houa)**[c]					
Dayes-Dodzi (Djodji)/ Kessibo-Dzodzi[d]	Wawa	Hyperendemic (enhanced) under annual CDTI	2000	2.2 (0.7–5.8)	60–89
**Zio River Basin (Volta Lac-East)**					
Tokpo	Agou	Hyperendemic (enhanced) under annual CDTI	2014	0.0 (0.0–2.4)	60–89

CDTI: Community-directed treatment with ivermectin
[a]Villages with the latest two (Kpogandji) or four (Kamalo-Kopé) surveys with 0% prevalence [15].
[b]Kokote is located around the Kpessi vector capture point of the Mono River Basin (Supplementary Table 4). In contrast to the modelled projections followed by most hypoendemic villages in Plateaux, the village of Babame, also in Kpessi, had a microfilarial prevalence of 0.6% (95%CI: 0.1–3.4%) in 2014 [15].
[c]In locations where the Djodji form of *Simulium sanctipauli* was present, recent data indicate still high biting rates following its elimination, by other species in the *damnosum* complex [54,55].
[d]Vector control started earlier, in 1981, in this village's river basin to eliminate the Djodji form of *Simulium sanctipauli* [18], preceding its first survey, in which hyperendemicity was determined.

Supplementary Table 23. Villages in Maritime not included in the special intervention zone (non-SIZ)

Village	Prefecture	Modelled prevalence trends followed by villages (intervention scenario)	Latest parasitological survey		EOT probability (%) if MDA stops in 2027
			Year	Microfilarial prevalence (%) (95% CI)	
**Yoto/Haho River Basin**					
Yoto-Kopé	Yoto	Hyperendemic (enhanced)	2005	0.0 (0.0–2.5)	60–89

7.2. Villages without recorded baseline microfilarial prevalence (without BMP)

Supplementary Table 24. Villages in Savanes included in the special intervention zone (SIZ)

Village	Prefecture	Modelled prevalence trends followed by villages (intervention scenario)	Latest parasitological survey		EOT probability (%) if MDA stops in 2027
			Year	Microfilarial prevalence (%) (95% CI)	
**Oti River Basin**					
Bonsougou	Oti	Hyperendemic (enhanced)	2007	0.0 (0.0–3.8)	60–89
Boutchakou	Oti	Hyperendemic (enhanced)	2015	0.8 (0.1–4.3)	60–89
Djandjatie	Oti	Hyperendemic (enhanced)	2011	0.6 (0.1–3.5)	60–89
Koukoumbou	Oti	Hyperendemic (minimal)	2015	3.7 (1.3–10.2)	<5
Kpatibori	Oti	Hyperendemic (minimal)	2014	9.1 (2.6–27.8)	<5
Kpintidjouaga	Kpendjal	Hyperendemic (enhanced)	2011	0.4 (0.1–2.1)	60–89
Moukaga	Kpendjal	Hyperendemic (enhanced)	2006	0.0 (0.0–4.4)	60–89
Naboli	Oti	Hyperendemic (enhanced)	2015	0.0 (0.0–2.6)	60–89
Nambossi	Oti	Hyperendemic (enhanced)	2011	0.4 (0.1–2.0)	60–89
Nassiele	Kpendjal	Hyperendemic (enhanced)	2006	1.3 (0.2–7.0)	60–89
Natoundjenga	Kpendjal	Hyperendemic (enhanced)	2011	0.0 (0.0–2.9)	60–89
Natounkpargou	Kpendjal	Hyperendemic with 100% vector control (enhanced)	2011	0.0 (0.0–1.6)	60–89
Pancerys[a]	Kpendjal	Hyperendemic (reference)	2015	1.5 (0.6–3.9)	20–59
Poporkou	Oti	Hyperendemic with 100% vector control (enhanced)	2015	0.0 (0.0–2.3)	60–89

[a]In 2015, the prevalence of *O. volvulus* infection in *Simulium damnosum* sensu lato was 0.2% (95% CI: 0.03-1.3%) in Pancerys [13].

Supplementary Table 24. Continued

Village	Prefecture	**Modelled prevalence trends followed by villages** (intervention scenario)	**Latest parasitological survey**		**EOT probability (%) if MDA stops in 2027**
			**Year**	**Microfilarial prevalence (%)** (95% CI)	
**Oti River Basin**					
Simbo[a]	Oti	Hyperendemic (reference)	2011	2.0 (0.7–5.7)	20–59
Sougtangou	Kpendjal	Hyperendemic (reference)	2011	1.3 (0.4–4.5)	20–59
Tchountchonga[a]	Oti	Hyperendemic (reference)	2011	0.5 (0.1–3.0)	20–59
Tchri[a]	Oti	Hyperendemic (minimal)	2015	1.0 (0.2–5.7)	<5
Yiyingou	Oti	Hyperendemic (enhanced)	2011	1.1 (0.4–3.1)	60–89
**Oti-Pendjari River Basin / Volta Blanche (White Volta)**					
Lokpano	Tandjouaré	Hyperendemic with 100% vector control (enhanced)	2014	0.5 (0.1–2.7)	60–89

[a]Villages for which it has been reported that the epidemiological situation was unsatisfactory, and may have received biannual CDTI in the late 1990's for approximately 2 years [56].

Supplementary Table 25. Villages in Kara included in the special intervention zone (SIZ)

Village	Prefecture	Modelled prevalence trends followed by villages (intervention scenario)	Latest parasitological survey		EOT probability (%) if MDA stops in 2027
			Year	Microfilarial prevalence (%) (95% CI)	
**Oti River Basin**					
Kpabte	Doufelgou	Hyperendemic (reference)	2006	4.9 (2.8–8.5)	60–89
Possao	Dankpen	Hyperendemic (minimal)	2014	1.1 (0.4–3.2)	5–19
**Kara River Basin**					
Aho-Lao	Kozah	Holoendemic (enhanced)	2000	22.8 (18.8–27.4)	<5
Djamde Kawa	Kozah	Hyperendemic (minimal)	2011	0.6 (0.1–3.1)	5–19
Kadjol II	Dankpen	Hyperendemic (minimal)	2014	0.5 (0.1–2.6)	5–19
Kawa-Bassar	Bassar	Hyperendemic (reference)	2015	0.0 (0.0–2.7)	60–89
Koulwere	Doufelgou	Hyperendemic (minimal)	2015	1.7 (0.6–4.9)	5–19
Sakponé	Dankpen	Hyperendemic (minimal)	2014	0.7 (0.2–2.3)	5–19
Sekou-Bas	Dankpen	Hyperendemic (reference)	2014	0.4 (0.1–2.2)	60–89
Sikan[a]	Dankpen	Holoendemic (enhanced)	2014	1.2 (0.5–3.0)	<5
Tchakassou	Bassar	Holoendemic (enhanced)	2015	4.2 (2.4–7.2)	<5
Touguel	Dankpen	Holoendemic (enhanced)	2014	1.0 (0.3–2.8)	<5
Wassi	Bassar	Holoendemic (reference)	2014	27.0 (18.2–38.1)	<5
**Kerán River Basin**					
Goulbi	Kéran	Holoendemic (enhanced)	2015	9.9 (5.9–16.1)	<5
Hourta	Kéran	Hyperendemic (minimal)	2015	2.3 (0.6–7.8)	5–19
Koffi-Ferme	Kéran	Holoendemic (enhanced)	2014	5.4 (1.8–14.6)	<5
Koutantagou/ Koutantagou & Tapount	Kéran	Hyperendemic (minimal)	2015	1.9 (0.3–9.8)	5–19

Supplementary Table 25. Continued

Village	Prefecture	Modelled prevalence trends followed by villages (intervention scenario)	Latest parasitological survey		EOT probability (%) if MDA stops in 2027
			Year	Microfilarial prevalence (%) (95% CI)	
**Kerán River Basin (continued)**					
Koutougou Solla	Kéran	Holoendemic (enhanced)	2015	13.6 (7.8–22.7)	<5
Kpantiiyagou	Kéran	Holoendemic (enhanced)	2015	7.7 (4.5–12.9)	<5
Narita / Pesside	Kéran	Holoendemic (enhanced)	2014	6.5 (3.3–12.3)	<5
Sola	Kéran	Holoendemic (enhanced)	2000	41.8 (32.2–52.0)	<5
Tchitchira Ferme	Kéran	Holoendemic (reference)	2002	59.0 (42.3–74.5)	<5
Wasite & Pesside Ferme/Wassite	Kéran	Holoendemic (reference)	2004	16.3 (11.6–22.4)	<5
Wartema	Kéran	Hyperendemic (minimal)	2002	25.5 (17.8–35.2)	5–19
**Mô River Basin**					
Dandjessi	Bassar	Hyperendemic (minimal)	2012	3.0 (1.5–6.1)	5–19
Katcha-Konkomba	Bassar	Hyperendemic (minimal)	2015	4.3 (2.3–7.7)	5–19
Kissafo	Bassar	Hyperendemic (minimal)	2012	3.3% 1.9–5.8)	5–19
Madjatom[b]	Bassar	Hyperendemic (minimal)	2015	0.7 (0.1–3.7)	5–19
Saboundi	Bassar	Hyperendemic (minimal)	2015	1.5 (0.3–5.7)	5–19

[a]Vector control was very effective in Sikan, bringing the annual transmission potential to 0 in 2006 [45]. Ivermectin treatment coverage was reported at 90% of total population in 2003 [57].
[b]Surveys with consistently low prevalence for 15 years [15].

Supplementary Table 26. Villages in Centrale included in the special intervention zone (SIZ)

Village	Prefecture	Modelled prevalence trends followed by villages (intervention scenario)	Latest parasitological survey		EOT probability (%) if MDA stops in 2027
			Year	Microfilarial prevalence (%) (95% CI)	
**Mô River Basin**					
Agbamassoumou	Sotouboua	Hyperendemic (reference)	2012	0.0 (0.0–1.5)	60–89
Assawoh-Koura	Sotouboua	Holoendemic (enhanced)	2015	10.3 (5.7–18.0)	<5
Banda	Sotouboua	Holoendemic (enhanced)	2015	4.4 (2.3–8.5)	<5
Batto	Sotouboua	Holoendemic (minimal)	2014	32.7 (21.2–46.6)	<5
Dantchessi	Sotouboua	Hyperendemic (minimal)	2006	8.5 (5.0–14.0)	5–19
Koida or Kouida	Sotouboua	Holoendemic (enhanced)	2015	5.8 (3.5–9.6)	<5
Moussoukoudjou	Sotouboua	Hyperendemic (reference)	2006	3.0 (1.0–8.5)	60–89
Naboun-Koura	Sotouboua	Hyperendemic (reference)	2009	0.6 (0.1–3.1)	60–89
Sakpagninga	Sotouboua	Holoendemic (enhanced)	2003	15.8 (9.4–25.0)	<5
Tchakpissi	Sotouboua	Holoendemic (enhanced)	2015	10.5 (4.2–24.1)	<5
Tchatou Koura	Sotouboua	Hyperendemic (minimal)	2015	3.3 (1.5–7.1)	5–19
Tchetchekou	Sotouboua	Hyperendemic (minimal)	2015	3.5 (1.6–7.4)	5–19

Supplementary Table 27. Villages in Centrale not included in the special intervention zone (non-SIZ)

Village	Prefecture	Modelled prevalence trends followed by villages (intervention scenario)	Latest parasitological survey		EOT probability (%) if MDA stops in 2027
			Year	Microfilarial prevalence (%) (95% CI)	
**Anié River Basin (Mono) / Asukawkaw**					
Agbandi-Mono	Blitta	Hyperendemic (enhanced)	2015	0.3 (0.1–1.8)	60–89
Yeloum Bagnan	Blitta	Hyperendemic (enhanced)	2012	1.3 (0.5–3.9)	60–89
Katchalikadi	Sotouboua	Hyperendemic (enhanced)	2012	1.6 (0.5–5.7)	60–89
Kpeida	Sotouboua	Mesoendemic (minimal)	2013	0.0 (0.0–2.0)	60–89
Okou-Kopé	Blitta	Mesoendemic (minimal)	2012	0.0 (0.0–3.0)	60–89
Panlao	Sotouboua	Mesoendemic (minimal)	2013	0.0 (0.0–1.9)	60–89
Yovo-Kopé	Blitta	Mesoendemic (minimal)	2012	0.0 (0.0–2.5)	60–89
**Mono River Basin**					
Akawolo	Tchamba	Mesoendemic (minimal)	2011	0.0 (0.0–1.6)	60–89
Kpambouré (Aou)	Sotouboua	Mesoendemic (minimal)	2002	3.3 (1.8–5.9)	60–89
Oudjomboi	Tchamba	Mesoendemic (minimal)	2011	0.5 (0.1–2.5)	60–89
Sada-Mono	Sotouboua	Mesoendemic (minimal)	2002	4.5 (2.7–7.2)	60–89
**Ogou River Basin (Mono)**					
Blou-Elavagnon	Tchamba	Mesoendemic (minimal)	2011	0.4 (0.1–2.5)	60–89
Ogouda & Sombo	Tchamba	Hyperendemic (reference)	2015	3.5 (1.2–9.7)	60–89
Soukounde	Tchamba	Mesoendemic (minimal)	2011	0.0 (0.0–4.2)	60–89
Talaba	Tchamba	Mesoendemic (minimal)	2011	0.0 (0.0–2.7)	60–89
**Kpaza Koue River Basin**					
Takade	Sotouboua	Hyperendemic (minimal)	2015	14.5 (10.7–19.2)	<5

Supplementary Table 28. Villages in Plateaux not included in the special intervention zone (non-SIZ)

Village	Prefecture	Modelled prevalence trends followed by villages (intervention scenario)	Latest parasitological survey		EOT probability (%) if MDA stops in 2027
			Year	Microfilarial prevalence (%) (95% CI)	
**Amou River Basin (Mono)**					
Amoutchou	Ogou	Hyperendemic (enhanced) under biannual CDTI since 2014	2012	1.5 (0.5–4.4)	60–89
Amouto (Amou-Oblo)	Haho	Hyperendemic (reference) under biannual CDTI since 2014	2012	2.7 (1.1–6.0)	5–19
Atinkpassa	Ogou	Hyperendemic (reference) under biannual CDTI since 2014	2017	3.4 (1.2–9.6)	5–19
Glelou & Omouva	Amou	Hyperendemic (enhanced) under biannual CDTI since 2014	2011	1.3 (0.5–3.8)	60–89
Igbowou-Amou[a]	Amou	Hyperendemic (minimal) under biannual CDTI since 2014	2017	6.9 (3.2–14.2)	<5
Ilekohan	Ogou	Hyperendemic (enhanced) under biannual CDTI since 2014	2012	1.2 (0.3–4.2)	60–89
Kpati Copé[a]	Amou	Hyperendemic (minimal) under biannual CDTI since 2014	2017	6.7 (3.1–13.9)	<5
Tsokple or Tchokple[a]	Amou	Hyperendemic (minimal) under biannual CDTI since 2014	2017	12.4 (7.2–20.4)	<5
**Anié River Basin (Mono)**					
Atewe-Zongo	Anié	Hyperendemic (enhanced) under annual CDTI	2015	0.5 (0.1–2.9)	60–89
Pidina	Amou	Hyperendemic (enhanced) under biannual CDTI since 2014	2014	0.7 (0.1–3.6)	60–89
Tchékélé	Ogou	Hyperendemic (enhanced) under biannual CDTI since 2014	2013	0.5 (0.1–3.0)	60–89

[a]Anti-OvAg seroprevalence in children under 15 years of age was 52.9% (9/17) in Igbowou-Amou, 60% (15/25) in Kpati Copé, and 48.4% (15/31) in Tsokple [26].

Supplementary Table 28. Continued

Village	Prefecture	Modelled prevalence trends followed by villages (intervention scenario)	Latest parasitological survey		EOT probability (%) if MDA stops in 2027
			Year	Microfilarial prevalence (%) (95% CI)	
**Deveho River Basin (Mono) / Zio River Basin**					
Tutu Zionou[a]	Kpélé	Hyperendemic (reference) under annual CDTI	2017	2.5 (0.7–8.8)	<5
**Menou (or Menu) River Basin (Asukawkaw)**					
Ahlon Dzindzi	Wawa	Mesoendemic (minimal) under annual CDTI	2008	0.5 (0.1–2.8)	60–89
Denou Bumuebi	Danyi	Hyperendemic (enhanced) under biannual CDTI since 2014	2008	2.2 (0.7–6.2)	60–89
Guin Kopé	Wawa	Hyperendemic (reference) under annual CDTI	2008	5.3 (3.3–8.4)	<5
Odomi Abra	Wawa	Hyperendemic (reference) under annual CDTI	2008	6.7 (4.2–10.6)	<5
S. Outouala	Danyi	Hyperendemic (enhanced) under biannual CDTI since 2014	2008	3.6 (1.7–7.8)	60–89
**Mono River Basin**					
Glive	Ogou	Hyperendemic (enhanced) under biannual CDTI since 2014	2012	2.4 (0.7–8.5)	60–89
Hetre	Ogou	Hyperendemic (enhanced) under biannual CDTI since 2014	2012	0.6 (0.1–3.2)	60–89
Moba Kopé	Ogou	Hyperendemic (enhanced) under biannual CDTI since 2014	2013	1.2 (0.3–4.2)	60–89
Tanago	Ogou	Hyperendemic (enhanced) under biannual CDTI since 2014	2012	1.1 (0.2–6.0)	60–89
Toigbo	Ogou	Hyperendemic (enhanced) under biannual CDTI since 2005	2005	6.9 (4.5–10.5)	60–89

[a]The only other surveyed village (Kouma-Kunda) from the Deveho (Mono) River Basin was classified as hypoendemic at baseline. In contrast to the model projections followed by most of the other hypoendemic villages in Plateaux, this village recorded a microfilarial prevalence of 1.3% (95%CI: 0.2–5.2%) in 2014 [15].

Supplementary Table 28. Continued

Village	Prefecture	**Modelled prevalence trends followed by villages** (intervention scenario)	**Latest parasitological survey**		**EOT probability (%) if MDA stops in 2027**
			**Year**	**Microfilarial prevalence (%)** (95% CI)	
**Todje (or Todzie) River Basin (Lake Volta)**					
Ananivikodzi	Agou	Hyperendemic (enhanced) under annual CDTI	2000	0.0 0.0–8.8)	60–89
Klo-Mayondi	Kloto	Hyperendemic (reference) under annual CDTI	2000	16.4 (12.5–21.3)	<5
Kpime-Seva (Tététou)	Kloto	Hyperendemic (reference) under annual CDTI	2000	12.5 (8.6–17.6)	<5
Nyive	Kloto	Hyperendemic (reference) under annual CDTI	2004	7.1 (4.1–12.0)	<5
Tome	Agou	Mesoendemic (minimal) under annual CDTI	2001	4.3 (2.4–7.5)	60–89
**Wawa/Asukawkaw River Basin**					
Sukul-Kpodji	Wawa	Hyperendemic (enhanced) under annual CDTI	2014	0.7 (0.1–4.1)	60–89

Supplementary Table 29. Villages in Maritime not included in the special intervention zone (non-SIZ)

Village	Prefecture	**Modelled prevalence trends followed by villages** (intervention scenario)	Latest parasitological survey		EOT probability (%) if MDA stops in 2027
			Year	**Microfilarial prevalence (%)** (95% CI)	
**Haho River Basin (Mono)**					
Afangadji[a]	Yoto	Hyperendemic (enhanced)	2017	1.3 (0.2–7.1)	60–89
Togba	Yoto	Hyperendemic (enhanced)	2015	0.6 (0.1–3.5)	60–89
**Mono River Basin**					
Afomonou	Bas-Mono	Mesoendemic (minimal)	2012	0.9 (0.2–4.9)	60–89
Dzrekpon/Djrekpon	Yoto	Hyperendemic (enhanced)	2015	1.6 (0.3–8.9)	60–89
Gbandidi	Bas-Mono	Mesoendemic (minimal)	2012	0.6 (0.1–3.1)	60–89
Gogokondji	Yoto	Hyperendemic (enhanced)	2017	0.0 (0.0–9.6)	60–89
Lakata-Kondji	Yoto	Hyperendemic (enhanced)	2015	0.0 (0.0–5.7)	60–89
Mawussou	Yoto	Hyperendemic (enhanced)	2015	1.2 (0.2–6.7)	60–89
**Zio River Basin**					
Afokonou	Zio	Mesoendemic (minimal)	2015	0.0 (0.0–5.7)	60–89
Kayido	Avé	Hyperendemic (reference) or Hyperendemic (enhanced)	2012	3.6 (1.0–12.3)	<5 or 60–89
Konta & Agbatehi	Avé	Hyperendemic (enhanced)	2012	2.4 (1.0–5.4)	60–89

CDTI: Community-directed treatment with ivermectin.

[a]In 2020-2023, stop-MDA surveys in Afangadji indicated active transmission [20].

Supplementary Text 8. Calculation of prefecture-level likelihood of reaching elimination of onchocerciasis transmission

The term "likelihood" is employed here as a categorical variable ('very likely', 'likely', 'possibly', 'unlikely' and 'very unlikely') to denote the range of joint elimination of transmission (EOT) probabilities, rather than referring to the formal statistical notion of likelihood used in inferential analyses. To calculate the likelihood of reaching EOT for each prefecture, we multiplied village-level EOT probabilities (for surveyed villages) using midpoint values across the village-level EOT probability ranges (Supplementary Table 30).

Supplementary Table 30. Assigned midpoint values for village-level EOT probability ranges

EOT probability range per village (%)	Midpoint value (%)
<5	2.5
5–19	12.0
20–59	39.5
60–89	74.5
≥90	100.0[a]

[a]For the ≥90% EOT probability, a value of 100.0 was used in the calculation of the prefecture-level EOT likelihood. Most villages projected to reach ≥90% EOT probabilities have values close to 100%; therefore, this approach prevents underestimating the overall likelihood in prefectures that have many villages with high EOT probabilities.

Each village's midpoint was used to calculate the joint EOT probability for the entire prefecture, by multiplying the midpoint probabilities (Table 26) of *all* surveyed villages within that prefecture (i.e., with or without recorded baseline microfilarial prevalence). This approach assumes independence of EOT probabilities across villages within prefectures, and across prefectures, as EPIONCHO-IBM models closed populations (i.e., not considering movement between villages or prefectures of humans or flies),

$$P_j = \prod_{i=1}^{i=n_j} P_{i,j}$$

Where P_j is the joint EOT probability for prefecture j ($j = 1, \ldots, 34$) and $P_{i,j}$ the midpoint EOT probability for village i in prefecture j. The calculated joint EOT probabilities for each prefecture were assigned to one of the five likelihood categories (Supplementary Table 31).

Supplementary Table 31. Definitions of prefecture-level EOT likelihood categories

EOT likelihood category	Probability range	Description
Very likely	≥90.00%	Very high probability of reaching EOT, with all surveyed villages being projected to reach ≥90% EOT probability
Likely	50.00–89.99%	High probability of reaching EOT, with most surveyed villages being projected to reach ≥90% EOT probability
Possibly	5.00–49.99%	Moderate probability of reaching EOT, with most surveyed villages being projected to reach at least 60–89% EOT probability
Unlikely	0.01–4.99%	Low probability of reaching EOT, reflecting the presence of surveyed villages with projected <20% EOT probability
Very unlikely	<0.01%	Very low probability of reaching EOT, indicating the presence of several surveyed villages with projected <5% EOT probability

Supplementary Tables 32-34 present prefecture-specific EOT likelihood categories when simulating that ivermectin MDA stops in 2024, 2027 or 2030, indicating the total number of surveyed villages per prefecture and the number of villages for each midpoint value. Extending treatment to 2027 slightly improves the EOT likelihood for the prefectures in Kara, although it does only alter the overall likelihood categories for Doufelgou from 'Unlikely' to 'Possibly' and for Binah (from 'Possibly' to 'Likely') (Table 33). Extending treatment to 2030 increases somewhat the EOT likelihood for Dankpen prefecture (from 'Very unlikely' to 'Unlikely') (Table 34). Under biannual MDA, extending treatment to 2030 improves the likelihood for Plateaux prefectures, with Haho and Ogou prefectures changing from 'Unlikely' to 'Possibly', and with Danyi prefecture changing from 'Possibly' to 'Very likely'. The villages within each prefecture with projected EOT probabilities <90% if MDA stops in 2027 are presented in Supplementary Tables 18-23 (for villages with recorded BMP estimates) and 24-29 (for those without recorded BMP estimates). As the calculation of joint EOT probabilities is strongly dependent on the number of surveyed villages in each prefecture, Figure 7 of the Main Text also shows, as pie-charts, the proportions of surveyed villages in each prefecture according to their projected EOT probability ranges if ivermectin MDA stops in 2027. The size of the pie-charts reflects the number of surveyed villages.

Supplementary Table 32. Prefecture-level likelihood of reaching EOT when simulating that ivermectin MDA stops in 2024

**Region** Prefecture	**No. villages**	**Midpoint value (%)** (EOT probability range per village, %)					**Joint EOT probability (%)**	**EOT likelihood category**
		**2.5%** (<5%)	**12.0%** (5–19%)	**39.5%** (20–59%)	**74.5%** (60–89%)	**100.0** (≥90%)		
**Savanes**								
Kpendjal, including Kpendjal-Ouest	9	0	0	3	5	1	1.4	Unlikely
Oti, including Oti-Sud	29	3	0	2	7	17	<0.01	Very unlikely
Tandjoaré or Tandjouaré	5	0	0	0	1	4	74.5	Likely
Tône, including Cinkassé	6	0	0	2	0	4	15.6	Possibly
**Kara**								
Assoli	3	0	0	0	0	3	≥90	Very likely
Bassar	17	2	8	0	0	7	<0.01	Very unlikely
Binah or Bimah	4	0	0	1	0	3	39.5	Possibly
Dankpen	19	2	3	1	1	12	<0.01	Very unlikely
Doufelgou	5	0	1	2	1	1	1.4	Unlikely
Kéran	13	10	3	0	0	0	<0.01	Very unlikely
Kozah	27	1	2	0	1	23	0.03	Unlikely

Supplementary Table 32. Continued

**Region**<br>Prefecture	**No. villages**	**Midpoint value (%)**<br>(EOT probability range per village, %)					**Joint EOT probability (%)**	**EOT likelihood category**
		**2.5%**<br>(<5%)	**12.0%**<br>(5–19%)	**39.5%**<br>(20–59%)	**74.5%**<br>(60–89%)	**100.0**<br>(≥90%)		
**Centrale**								
Blitta	28	0	0	0	10	18	5.3	Possibly
Sotouboua, including Mô	34	7	3	0	14	10	<0.01	Very unlikely
Tchamba	16	0	0	0	7	9	12.7	Possibly
Tchaoudjo or Tchaudjo	8	0	1	0	2	5	6.7	Possibly
**Plateaux**								
Agou	13	0	0	0	3	10	30.8	Possibly
Akébou	2	0	0	0	1	1	74.5	Likely
Amou	8	3	0	0	3	2	<0.01	Very unlikely
Anié	12	0	0	0	3	9	41.4	Possibly
Danyi	5	0	0	0	2	3	55.5	Likely
Est-Mono	15	0	0	0	4	11	30.8	Possibly
Haho	21	0	3	0	3	15	0.07	Unlikely
Kloto	5	3	0	0	0	2	<0.01	Very unlikely

Supplementary Table 32. Continued

**Region** Prefecture	**No. villages**	**Midpoint value (%)** (EOT probability range per village, %)					**Joint EOT probability (%)**	**EOT likelihood category**
		**2.5%** (<5%)	**12.0%** (5–19%)	**39.5%** (20–59%)	**74.5%** (60–89%)	**100.0** (≥90%)		
**Plateaux (continued)**								
Kpélé	2	1	0	0	0	1	2.5	Unlikely
Moyen-Mono	6	0	0	0	4	2	30.8	Possibly
Ogou	37	0	2	0	16	19	0.01	Unlikely
Wawa	10	2	0	0	4	4	0.02	Unlikely
**Maritime**								
Avé[a]	5	0	0	1	1	3	29.4	Possibly
Bas-Mono, includes areas previously from Lacs[a]	2	0	0	0	2	0	55.5	Likely
Golfe, including Lomé and Agoè-Nyivé[a]	0	0	0	0	0	0	–	Non-endemic
Lacs[b]	0	0	0	0	0	0	–	Non-endemic
Vo[a]	0	0	0	0	0	0	–	Non-endemic
Yoto[a]	22	0	0	4	3	15	1.0	Unlikely
Zio[a]	12	0	0	0	1	11	74.5	Likely

[a]In all of Maritime, excepting the endemic villages in the Haho River Basin (Mono) of Yoto Prefecture, control interventions may have stopped earlier than 2024 [15].

Supplementary Table 33. Prefecture-level likelihood of reaching EOT when simulating that ivermectin MDA stops in 2027

**Region** Prefecture	**No. villages**	**Midpoint value (%)** (EOT probability range per village, %)					**Joint EOT probability (%)**	**EOT likelihood category**
		**2.5%** (<5%)	**12.0%** (5–19%)	**39.5%** (20–59%)	**74.5%** (60–89%)	**100.0** (≥90%)		
**Savanes**								
Kpendjal, including Kpendjal-Ouest	9	0	0	3	5	1	1.4	Unlikely
Oti, including Oti-Sud	29	3	0	2	7	17	<0.01	Very unlikely
Tandjoaré or Tandjouaré	5	0	0	0	1	4	74.5	Likely
Tône, including Cinkassé	6	0	0	2	0	4	15.6	Possibly
**Kara**								
Assoli	3	0	0	0	0	3	≥90	Very likely
Bassar	17	2	6	0	2	7	<0.01	Very unlikely
Binah or Bimah	4	0	0	0	1	3	74.5	Likely
Dankpen	19	2	3	0	1	13	<0.01	Very unlikely
Doufelgou	5	0	1	0	2	2	6.7	Possibly
Kéran	13	10	3	0	0	0	<0.01	Very unlikely
Kozah	27	1	2	0	0	24	0.04	Unlikely

Supplementary Table 33. Continued

**Region**<br>Prefecture	**No. villages**	**Midpoint value (%)**<br>(EOT probability range per village, %)					**Joint EOT probability (%)**	**EOT likelihood category**
		**2.5%**<br>(<5%)	**12.0%**<br>(5–19%)	**39.5%**<br>(20–59%)	**74.5%**<br>(60–89%)	**100.0**<br>(≥90%)		
**Centrale**								
Blitta	28	0	0	0	10	18	5.3	Possibly
Sotouboua, including Mô	34	7	3	0	14	10	<0.01	Very unlikely
Tchamba	16	0	0	0	7	9	12.7	Possibly
Tchaoudjo or Tchaudjo	8	0	1	0	2	5	6.7	Possibly
**Plateaux**								
Agou	13	0	0	0	3	10	30.8	Possibly
Akébou	2	0	0	0	1	1	74.5	Likely
Amou	8	3	0	0	3	2	<0.01	Very unlikely
Anié	12	0	0	0	3	9	41.4	Possibly
Danyi	5	0	0	0	2	3	55.5	Likely
Est-Mono	15	0	0	0	4	11	30.8	Possibly
Haho	21	0	3	0	3	15	0.07	Unlikely
Kloto	5	3	0	0	0	2	<0.01	Very unlikely

Supplementary Table 33. Continued

**Region**<br>Prefecture	**No. villages**	**Midpoint value (%)**<br>(EOT probability range per village, %)					**Joint EOT probability (%)**	**EOT likelihood category**
		**2.5%**<br>(<5%)	**12.0%**<br>(5–19%)	**39.5%**<br>(20–59%)	**74.5%**<br>(60–89%)	**100.0**<br>(≥90%)		
**Plateaux (continued)**								
Kpélé	2	1	0	0	0	1	2.5	Unlikely
Moyen-Mono	6	0	0	0	4	2	30.8	Possibly
Ogou	37	0	2	0	16	19	0.01	Unlikely
Wawa	10	2	0	0	4	4	0.02	Unlikely
**Maritime**								
Avé[a]	5	0	0	1	1	3	29.4	Possibly
Bas-Mono, includes areas previously from Lacs[a]	2	0	0	0	2	0	55.5	Likely
Golfe, including Lomé and Agoè-Nyivé[a]	0	0	0	0	0	0	–	Non-endemic
Lacs[a]	0	0	0	0	0	0	–	Non-endemic
Vo[a]	0	0	0	0	0	0	–	Non-endemic
Yoto[a]	22	0	0	4	3	15	1.0	Unlikely
Zio[a]	12	0	0	0	1	11	74.5	Likely

[a]In all of Maritime, excepting the endemic villages in the Haho River Basin (Mono) of Yoto Prefecture, control interventions may have stopped earlier than 2024 [20].

Supplementary Table 34. Prefecture-level likelihood of reaching EOT when simulating that ivermectin MDA stops in 2030

**Region** Prefecture	**No. villages**	**Midpoint value (%)** (EOT probability range per village, %)					**Joint EOT probability (%)**	**EOT likelihood category**
		**2.5%** (<5%)	**12.0%** (5–19%)	**39.5%** (20–59%)	**74.5%** (60–89%)	**100.0** (≥90%)		
**Savanes**								
Kpendjal, including Kpendjal-Ouest	9	0	0	3	5	1	1.4	Unlikely
Oti, including Oti-Sud	29	3	0	2	7	17	<0.01	Very unlikely
Tandjoaré or Tandjouaré	5	0	0	0	1	4	74.5	Likely
Tône, including Cinkassé	6	0	0	2	0	4	15.6	Possibly
**Kara**								
Assoli	3	0	0	0	0	3	≥90	Very likely
Bassar	17	2	0	6	2	7	<0.01	Very unlikely
Binah or Bimah	4	0	0	0	1	3	74.5	Likely
Dankpen	19	2	0	3	1	13	0.01	Unlikely
Doufelgou	5	0	0	1	2	2	19.2	Possibly
Kéran	13	10	0	3	0	0	<0.01	Very unlikely
Kozah	27	1	0	2	0	24	0.30	Unlikely

Supplementary Table 34. Continued

**Region** Prefecture	**No. villages**	**Midpoint value (%)** (EOT probability range per village, %)					**Joint EOT probability (%)**	**EOT likelihood category**
		**2.5%** (<5%)	**12.0%** (5–19%)	**39.5%** (20–59%)	**74.5%** (60–89%)	**100.0** (≥90%)		
**Centrale**								
Blitta	28	0	0	0	10	18	5.3	Possibly
Sotouboua, including Mô	34	7	0	3	14	10	<0.01	Very unlikely
Tchamba	16	0	0	0	7	9	12.7	Possibly
Tchaoudjo or Tchaudjo	8	0	1	0	2	5	6.7	Possibly
**Plateaux**								
Agou	13	0	0	0	3	10	30.8	Possibly
Akébou	2	0	0	0	1	1	74.5	Likely
Amou	8	3	0	0	0	5	<0.01	Very unlikely
Anié	12	0	0	0	3	9	41.4	Possibly
Danyi	5	0	0	0	0	5	≥90	Very likely
Est-Mono	15	0	0	0	4	11	30.8	Possibly
Haho	21	0	0	3	0	18	6.2	Possibly
Kloto	5	3	0	0	0	2	<0.01	Very unlikely

Supplementary Table 34. Continued

Region<br>Prefecture	No. villages	Midpoint value (%)<br>(EOT probability range per village, %)					Joint EOT probability (%)	EOT likelihood category
		**2.5%**<br>(<5%)	**12.0%**<br>(5–19%)	**39.5%**<br>(20–59%)	**74.5%**<br>(60–89%)	**100.0**<br>(≥90%)		
**Plateaux (continued)**								
Kpélé	2	1	0	0	0	1	2.5	Unlikely
Moyen-Mono	6	0	0	0	4	2	30.8	Possibly
Ogou	37	0	0	2	0	35	15.6	Possibly
Wawa	10	2	0	0	4	4	0.02	Unlikely
**Maritime**								
Avé[a]	5	0	0	1	1	3	29.4	Possibly
Bas-Mono, includes areas previously from Lacs[a]	2	0	0	0	2	0	55.5	Likely
Golfe, including Lomé and Agoè-Nyivé[a]	0	0	0	0	0	0	–	Non-endemic
Lacs[a]	0	0	0	0	0	0	–	Non-endemic
Vo[a]	0	0	0	0	0	0	–	Non-endemic
Yoto[a]	22	0	0	4	3	15	1.0	Unlikely[b]
Zio[a]	12	0	0	0	1	11	74.5	Likely

[a]In all of Maritime, excepting the endemic villages in the Haho River Basin (Mono) of Yoto Prefecture, control interventions may have stopped earlier than 2024 [20].
[b]Biannual MDA not modelled.

Supplementary Table 35. Prefecture-level EoT likelihood category, current interventions and recommendations

**Region**<br>Prefecture	**EOT likelihood category**<br>2024/2027/2030	**Current intervention**	**Recommendations[a]**
**Savanes**			
Kpendjal, including Kpendjal-Ouest	Unlikely/Unlikely/Unlikely	Biannual ivermectin MDA	ATS (e.g. biannual moxidectin MDA)
Oti, including Oti-Sud	Very unlikely/Very unlikely/Very unlikely	Biannual ivermectin MDA	
Tandjoaré or Tandjouaré	Likely/Likely/Likely	Annual ivermectin MDA	Insufficient data from representative endemic villages to provide an evidence-based recommendation
Tône, including Cinkassé	Possibly/Possibly/Possibly	Annual ivermectin MDA	
**Kara**			
Assoli	Very likely/Very likely/Very likely	Biannual ivermectin MDA	Proceed to Stop-MDA assessment
Bassar	Very unlikely/Very unlikely/Very unlikely	Biannual ivermectin MDA	ATS (e.g. biannual moxidectin MDA)
Binah or Bimah	Possibly/Likely/Likely	Biannual ivermectin MDA	Continue biannual ivermectin until 2027 followed by stop-MDA assessment. If unsuccessful, consider ATS (e.g. biannual moxidectin)
Doufelgou	Unlikely/Possibly/Possibly	Biannual ivermectin MDA	
Dankpen	Very unlikely/Very unlikely/Unlikely	Biannual ivermectin MDA	ATS (e.g. biannual moxidectin MDA)
Kéran	Very unlikely/Very unlikely/Very unlikely	Biannual ivermectin MDA	
Kozah	Unlikely/Unlikely/Unlikely	Biannual ivermectin MDA	

Supplementary Table 35. Continued

**Region**<br>Prefecture	**EOT likelihood category**<br>2024/2027/2030	**Current intervention**	**Recommendations**[a]
**Centrale**			
Blitta	Possibly/Possibly/Possibly	Annual ivermectin MDA	Proceed to Stop-MDA assessment. If unsuccessful, consider biannual ivermectin
Sotouboua, including Mô	Very unlikely/Very unlikely/Very unlikely	Biannual ivermectin MDA	ATS (e.g. biannual moxidectin MDA)
Tchamba	Possibly/Possibly/Possibly	Annual ivermectin MDA	Proceed to Stop-MDA assessment. If unsuccessful, consider biannual ivermectin
Tchaoudjo or Tchaudjo	Possibly/Possibly/Possibly	Biannual ivermectin MDA	Proceed to Stop-MDA assessment. If unsuccessful, consider ATS (e.g. biannual moxidectin MDA)
**Plateaux**			
Agou	Possibly/Possibly/Possibly	Annual ivermectin MDA	Proceed to Stop-MDA assessment. If unsuccessful, consider biannual ivermectin
Akébou	Likely/Likely/Likely	Annual ivermectin MDA	
Amou	Very unlikely/Very unlikely/Very unlikely	Biannual ivermectin MDA	ATS (e.g. biannual moxidectin MDA)
Anié	Possibly/Possibly/Possibly	Annual ivermectin MDA	Proceed to Stop-MDA assessment. If unsuccessful, consider biannual ivermectin
Danyi	Likely/Likely/Very likely	Biannual ivermectin MDA	Proceed to Stop-MDA assessment. If unsuccessful, continue biannual ivermectin MDA until 2030; then repeat Stop-MDA assessment
Est-Mono	Possibly/Possibly/Possibly	Annual ivermectin MDA	Proceed to Stop-MDA assessment. If unsuccessful, consider biannual ivermectin
Haho	Unlikely/Unlikely/Possibly	Biannual ivermectin MDA	Continue biannual ivermectin MDA until 2030 followed by stop-MDA assessment. If unsuccessful, consider ATS (e.g. biannual moxidectin)
Kloto	Very unlikely/Very unlikely/Very unlikely	Annual ivermectin MDA	ATS (e.g. biannual moxidectin MDA)

Supplementary Table 35. Continued

**Region**<br>Prefecture	**EOT likelihood category**<br>2024/2027/2030	**Current intervention**	**Recommendations**[a]
**Plateaux (continued)**			
Kpélé	Unlikely/Unlikely/Unlikely	Annual ivermectin MDA	Consider biannual ivermectin
Moyen-Mono	Possibly/Possibly/Possibly	Biannual ivermectin MDA	Proceed to Stop-MDA assessment. If unsuccessful, consider ATS (e.g. biannual moxidectin)
Ogou	Unlikely/Unlikely/Possibly	Biannual ivermectin MDA	Continue biannual ivermectin MDA until 2030 followed by stop-MDA assessment. If unsuccessful, consider ATS (e.g. biannual moxidectin)
Wawa	Unlikely/Unlikely/Unlikely	Annual ivermectin MDA	Consider biannual ivermectin
**Maritime**			
Avé[a]	Possibly/Possibly/Possibly	Stop-MDA assessment successful in 2022	Post-treatment surveillance (2025-2027). If successful proceed to post-elimination surveillance
Bas-Mono, includes areas previously from Lacs[a]	Likely/Likely/Likely	Stop-MDA assessment successful in 2022	
Golfe, including Lomé and Agoè-Nyivé[a]	Non-endemic	Stop-MDA assessment successful in 2017	Non-endemic. No further interventions needed
Lacs[a]	Non-endemic	Stop-MDA assessment successful 2017	
Vo[a]	Non-endemic	Stop-MDA assessment successful in 2017	
Yoto[a]	Unlikely/Biannual ivermectin MDA not modelled/Biannual ivermectin MSA not modelled	Annual ivermectin MDA; switched to biannual MDA in 2023	Continue biannual ivermectin
Zio[a]	Likely/Likely/Likely	Stop-MDA assessment successful in 2022	Post-treatment surveillance (2025-2027). If successful proceed to post-elimination surveillance

[a]ATS: alternative treatment strategies, e.g. moxidectin MDA [37,58], focal vector control (ground larviciding, slash and clear) [59,60]; Stop-MDA assessment using Ov16 serology and molecular xenomonitoring of blackflies according to WHO (2016) [51].

Supplementary Text 9. Modelling for policy: PRIME-NTD

We adhered to the Five Principles of the Neglected Tropical Disease (NTD) Modelling Consortium for good practice in policy-relevant NTD modelling [61]. Supplementary Table 36 briefly describes the five tenets, how they were fulfilled and where in the Main Text and/or Supplementary Material they can be found.

Supplementary Table 36. Policy-Relevant Items for Reporting Models in Epidemiology of Neglected Tropical Diseases (PRIME-NTD) summary table

Principle	What has been done to satisfy the principle?	Where in the manuscript is this described?
**Stakeholder engagement**	Discussions with a range of modelling and policy-focused collaborators, including local stakeholders in Togo	Author list. Acknowledgements section
**Complete model documentation**	References to the full description of EPIONCHO-IBM provided. Link to EPIONCHO-IBM full code given	Methods section: EPIONCHO-IBM. Supplementary Material Text 3. Data availability and Code availability sections of Main Text
**Complete description of data used**	The data used have been published [15] and are described in the Main Text and the Supplementary Material, which complement the published database, with cited references	Methods section: Prevalence data. Supplementary Material. Reference list of Main Text and Supplementary references
**Communicating uncertainty**	Sensitivity and uncertainty analyses performed by simulating a range of parameters, e.g., coverage of ivermectin mass drug administration (MDA) = 50-80% of total population; vector control efficacy = 60-100%; proportion of systematic non-adherence (SNA) = 1-5%, across four baseline microfilarial prevalence endemicity settings = hypoendemic, 30%; mesoendemic, 50%; hyperendemic, 70% and holoendemic, 90% and three intervention scenarios = minimal,	Methods sections: Modelling interventions and scenarios; Elimination probabilities. Tables in Main Text and Supplementary Material. Results section: Prevalence trends by region; Elimination probabilities. Figures 2-6 of Main Text and Supplementary Fig. 8-14. Supplementary Tables

	reference and enhanced, to provide upper and lower bounds for model outputs on microfilarial prevalence temporal trends across regions. One hundred model runs conducted for each endemicity setting and intervention scenario. Ninety-five percent confidence intervals (or ranges, or interquartile ranges) calculated and presented for data	
**Testable model outcomes**	Dynamics of microfilarial prevalence until 2030 modelled under historical and current interventions, showing model outputs together with village survey data to visualise agreement between modelling results and data (for nearly 400 villages and >1600 surveys, conducted between 1975 and 2017). Projected probabilities of reaching elimination of onchocerciasis transmission when simulating that MDA stops in 2024, 2027 or 2030. These probabilities can be compared with observations as stop-MDA surveys have been/are being conducted by the Ministère de la Santé et de l'Hygiène Publique of Togo	Results and Discussion sections of Main Text: Figures 2-7. Supplementary Tables and Figures

Supplementary References

1. World Health Organization, Onchocerciasis Control Programme in the Volta River Basin Area & Brinkmann, U. K. Baseline data on the epidemiology of onchocerciasis in Northern Togo. Onchocerciasis Control Programme in the Volta River Basin Area. https://iris.who.int/handle/10665/339680 (1977).

2. World Health Organization, Onchocerciasis Control Programme in the Volta River Basin Area & Brinkmann, U. K. Medical aspects of onchocerciasis in southern Togo. Onchocerciasis Control Programme in the Volta River Basin Area. https://iris.who.int/handle/10665/339032 (1977).

3. Amazigo, U., Noma, M., Bump, J., Benton, B., Liese, B., Yaméogo, L., Zouré, H. & Sékétéli, A. Chapter 15: Onchocerciasis. In: Jamison, D. T., Feachem, R. G., Makgoba, M. W. et al. (Editors). Disease and Mortality in Sub-Saharan Africa. Second edition. Washington (DC): The International Bank for Reconstruction and Development / The World Bank. https://www.ncbi.nlm.nih.gov/books/NBK2287/ (2006).

4. Cheke, R. C., Post, R. J. & Boakye, D. A. Seasonal variations and other changes in the geographical distributions of different cytospecies of the *Simulium damnosum* complex (Diptera: Simuliidae) in Togo and Benin. *Acta Trop*. **245**, 106970 (2023).

5. O'Hanlon, S. J., Slater, H. C., Cheke, R. A., Boatin, B. A., Coffeng, L. E., Pion, S. D. S., Boussinesq, M., Zouré, H. G.M., Stolk, W. A. & Basáñez, M.-G. Model-based geostatistical mapping of the prevalence of *Onchocerca volvulus* in West Africa. *PLoS Negl. Trop. Dis*. **10**, e0004328 (2016).

6. World Health Organization & Onchocerciasis Control Programme in the Volta River Basin Area. Final report on the Programme extension studies in Bénin, Ghana and Togo. Onchocerciasis Control Programme in the Volta River Basin Area. https://iris.who.int/handle/10665/312303 (1981).

7 World Health Organization, United Nations Development Programme, Food and Agriculture Organization of the United Nations & International Bank for Reconstruction and Development. Onchocerciasis control in the Volta river basin area: report of the mission for preparatory assistance to the governments of Dahomey, Ghana, Ivory Coast, Mali, Niger, Togo and Upper Volta. World Health Organization. https://iris.who.int/handle/10665/277239 (1973).

8. Dadzie, Y., Neira, M. & Hopkins, D. Final report of the Conference on the Eradicability of Onchocerciasis. *Filaria J.* **2,** 2 (2003).

9. Boatin, B. The Onchocerciasis Control Programme in West Africa (OCP). *Ann. Trop. Med. Parasitol.* **102(Suppl 1)**, S13–S17 (2008).

10. World Health Organization & African Programme for Onchocerciasis Control. Report of the fifth activity review and planning meeting of the Special Intervention Zones (SIZ): Ouagadougou, 8-10 November 2006. https://apps.who.int/iris/handle/10665/276197?show=full (2006).

11. Yaméogo, L. Special intervention zones. *Ann. Trop. Med. Parasitol*. **102(Suppl 1)**, S23–S24 (2008).

12. World Health Organization & Onchocerciasis Control Programme in West Africa. Onchocerciasis control in special intervention zones including Sierra Leone in the OCP area. Plan of action and budget. Ouagadougou, 4-6 December 2002. https://iris.who.int/handle/10665/342151 (2002).

13. Komlan, K., Vossberg, P. S., Gantin, R. G., Solim, T., Korbmacher, F., Banla, M., Padjoudoum, K., Karabou, P., Köhler, C. & Soboslay, P. T. *Onchocerca volvulus* infection and serological prevalence, ocular onchocerciasis and parasite transmission in northern and central Togo after decades of *Simulium damnosum* s.l. vector control and mass drug administration of ivermectin. *PLoS Negl. Trop. Dis.* **12**, e0006312 (2018).

14. End Neglected Tropical Diseases in Africa. Semi Annual Report (October 1, 2016–March 31, 2017). https://web.archive.org/web/20230729131933/https://endinafrica.org/wp-content/uploads/2018/04/END-in-Africa-Semi-Annual-Report-No-13.pdf (2017).

15. Vinkeles Melchers, N. V. S., Agoro, S., Togbey, K., Padjoudoum, K., Telou, I. G., Karabou, P., Djatho, T., Datagni, M., Dorkenoo, A. M., Kassankogno, Y., Bronzan, R. & Stolk, W. A. Impact of ivermectin and vector control on onchocerciasis transmission in Togo: Assessing the empirical evidence on trends in infection and entomological indicators. *PLoS Negl. Trop. Dis.* **18**, e0012312 (2024).

16. Act to End NTDs West. FY20. Togo annual work plan (October 1, 2019–September 30, 2020). https://web.archive.org/web/20250429224229/https://www.actntdswest.org/sites/default/files/2019-12/Togo_FY20%20Workplan%20narrative%20USAID%20CLEAN_web.pdf (2020).

17. Gnossike, P. [Country progress towards NTD 2030 Road Map targets for onchocerciasis in Togo: Progress, challenges and critical actions]. In French. World Health Organization & Global Onchocerciasis Network for Elimination (GONE) Togo Webinar, 11 June 2024. https://www.youtube.com/watch?v=jiGxyDWHJy8&list=PLxLC0k8G1p6nvHpAwo1gde2XnNEEerrMM&index=5 (2024).

18. Cheke, R. A., Fiasorgbor, G. K., Walsh, J. F. & Yaméogo, L. Elimination of the Djodji form of the blackfly *Simulium sanctipauli sensu stricto* as a result of larviciding by the WHO Onchocerciasis Control Programme in West Africa. *Med. Vet. Entomol.* **22**, 172–174 (2008).

19. World Health Organization, Onchocerciasis Control Programme in the Volta River Basin & Renz, A. Studies on the reinvasion by *Simulium damnosum* s.l. into the Eastern areas of Onchocerciasis Control Programme and on the vectorial capacity of different species of the *S. damnosum* complex in Togo and Benin 1982. Ouagadougou: Onchocerciasis Control Programme in the Volta River Basin Area. https://iris.who.int/bitstream/handle/10665/326643/326643-eng.pdf;jsessionid=EEC4311DA0CAF18F5AAE7D8A86D56BA7?sequence=1 (1982).

20. Act to End NTDs West. FY23. Work plan-Togo (October 1, 2022–September 30, 2023). https://web.archive.org/web/20240326094740/https://www.actntdswest.org/sites/default/files/inline-files/Act%20West%20FY23%20Workplan-Togo.pdf (2023).

21. Organisation Mondiale de la Santé & Programme de Lutte contre l'Onchocercose en Afrique de l'Ouest. Rapport sur l'état d'avancement du processus de mise en œuvre des activités transférées du programme de lutte contre l'onchocercose (1er septembre 1997–31 août 1998): Togo. Accra 7-9 décembre 1998. https://iris.who.int/handle/10665/311267 (1998).

22. Organisation Mondiale de la Santé & Programme de Lutte contre l'Onchocercose en Afrique de l'Ouest. Rapport sur l'état d'avancement du processus de mise en œuvre des activités transférées aux programmes nationaux de lutte contre l'onchocercose (1er septembre 1999–31 août 2000): Togo. Yaoundé 14-15 décembre 2000. https://iris.who.int/bitstream/handle/10665/311397/JPC21.6k-fre.pdf (2000).

23. Organisation Mondiale de la Santé & Programme de Lutte contre l'Onchocercose en Afrique de l'Ouest. Rapport sur l'état d'avancement du processus de mise en œuvre des activités transférées aux programmes nationaux de lutte contre l'onchocercose (1er janvier–31 septembre 2001): Togo. Washington D.C. 10-11 décembre 2001. https://iris.who.int/handle/10665/311536 (2001).

24. Organisation Mondiale de la Santé & Programme de Lutte contre l'Onchocercose en Afrique de l'Ouest. Rapport sur l'état d'avancement du processus de mise en œuvre des activités transférées aux programmes nationaux de lutte contre l'onchocercose (1er janvier–30 novembre 2002): Togo. Ouagadougou 4-6 décembre 2002. https://iris.who.int/handle/10665/311509 (2002).

25. Biritwum, R. B., Sylla, M., Diarra, T., Amankwa, J., Brika, G. P., Assogba, L. A. & Traore, M. O. Evaluation of ivermectin distribution in Benin, Côte d'Ivoire, Ghana and Togo: estimation of coverage of treatment and operational aspects of the distribution system. *Ann. Trop. Med. Parasitol.* **91**, 297–305 (1997).

26. Korbmacher, F., Komlan, K., Gantin, R. G., Poutouli, W. P., Padjoudoum, K., Karabou, P., Soboslay, P. T. & Köhler, C. *Mansonella perstans*, *Onchocerca volvulus* and *Strongyloides stercoralis* infections in rural populations in central and southern Togo. *Parasite Epidemiol. Control* **3**, 77–87 (2018).

27. Hill, E., Hall, J., Letourneau, I. D., Donkers, K., Shirude, S., Pigott, D. N., Hay, S. I. & Cromwell, E. A. A database of geopositioned onchocerciasis prevalence data. *Sci. Data* **6**, 67 (2019).

28. Noma, M., Zouré, H. G. M., Tekle, A. H., Enyong, P. A. I., Nwoke, B. E. B. & Remme, J. H. F. The geographic distribution of onchocerciasis in the 20 participating countries of the African Programme for Onchocerciasis Control: (1) priority areas for ivermectin treatment. *Parasit. Vectors* **7**, 325 (2014).

29. Johanns, S. I., Gantin, R. G., Wangala, B., Komlan, K., Halatoko, W. A., Banla, M., Karabou, P., Luty, A. J., Schulz-Key, H., Köhler, C. & Soboslay, P. T. *Onchocerca volvulus*-specific antibody and cellular responses in onchocerciasis patients treated annually with ivermectin for 30 years and exposed to parasite transmission in central Togo. *PLoS Negl. Trop. Dis*. **16**, e0010340 (2022).

30. Golden, A., Faulx, D., Kalnoky, M., Stevens, E., Yokobe, L., Peck, R., Karabou, P., Banla, M., Rao, R., Adade, K., Gantin, R. G., Komlan, K., Soboslay, P. T., de Los Santos, T & Domingo GJ.

Analysis of age-dependent trends in Ov16 IgG4 seroprevalence to onchocerciasis. *Parasit. Vectors* **9**, 338 (2016).

31. R Core Team. R: A Language and Environment for Statistical Computing. R Foundation for Statistical Computing, Vienna, Austria. https://www.r-project.org/ (2025).

32. RStudio Team. RStudio: Integrated Development Environment for R. RStudio, Inc., Boston, MA, USA. https://global.rstudio.com/categories/rstudio-ide/ (2025).

33. Imperial College Research Computing Service. Imperial College London. https://doi.org/10.14469/hpc/2232 (2025).

34. Duerr, H. P., Leary, C. C. & Eichner, M. High infection rates at low transmission potentials in West African onchocerciasis. *Int. J. Parasitol.* **36**, 1367–1372 (2006).

35. Walker, M., Stolk, W. A., Dixon, M. A., Bottomley, C., Diawara, L., Traoré, M. O, de Vlas, S. J. & Basáñez, M.-G. Modelling the elimination of river blindness using long-term epidemiological and programmatic data from Mali and Senegal. *Epidemics* **18**, 4–15 (2017).

36. Hamley, J. I. D., Milton, P., Walker, M. & Basáñez, M.-G. Modelling exposure heterogeneity and density dependence in onchocerciasis using a novel individual-based transmission model, EPIONCHO-IBM: Implications for elimination and data needs. *PLoS Negl. Trop. Dis.* **13**, e0007557 (2019).

37. Kura, K., Milton, P., Hamley, J. I. D., Walker, M., Bakajika, D. K., Kanza, E. M., Opoku, N. O., Howard, H., Nigo, M. M., Asare, S., Olipoh, G., Attah, S. K., Mambandu, G. L., Kennedy, K. K., Kataliko, K., Mumbere, M., Halleux, C. M., Hopkins, A., Kuesel, A. C., Kinrade, S. & Basáñez, M.-G. Can mass drug administration of moxidectin accelerate onchocerciasis elimination in Africa? *Philos. Trans. R. Soc. Lond. B Biol. Sci.* **378**, 20220277 (2023).

38. Ramani, A., Stapley, J. N., Dixon, M. A., Hamley, J. I. D., Amaral, L.-J., Basáñez, M.-G. & Walker, M. Modelling anti-Ov16 seroprevalence for the control and elimination of onchocerciasis. https://doi.org/10.21203/rs.3.rs-7140160/v1 (2025).

39. World Health Organization, Onchocerciasis Control Programme in the Volta River Basin Area, Walsh, J. F., Davies, J. B. & Le Berre, R. Methods of entomological evaluation currently in use by VCU with suggestions for establishing criteria for advising on resettlement and development projects. https://iris.who.int/handle/10665/363566 (1977).

40. Organisation Mondiale de la Santé, Programme de Lutte contre l'Onchocercose en Afrique de l'Ouest & Hyacinthe, A. Rapport de synthèse des activités de l'OCP sur les affluents de l'Oti (Keran-Kara-Mo) de 1976 à 2001. https://iris.who.int/handle/10665/367530 (2002).

41. Organisation Mondiale de la Santé & Programme de Lutte contre l'Onchocercose dans la Région du Bassin de la Volta. Rapport final des études d'extensions du programme au Benin, Ghana et Togo. Genève 12-16 octobre 1981. https://iris.who.int/handle/10665/279861 (1981).

42. Organisation Mondiale de la Santé & Programme de Lutte contre l'Onchocercose dans la Région du Bassin de la Volta. Situation au 1er septembre 1979 des études dans les zones d'extension du programme. Genève 3-5 décembre 1979. https://iris.who.int/handle/10665/311765 (1979).

43. De Sole, G., Accorsi, S., Cresveaux, H., Remme, J., Walsh, F. & Hendrickx, J. Distribution and severity of onchocerciasis in southern Benin, Ghana and Togo. *Acta Trop*. **52**, 87–97 (1992).

44. Hougard, J. M., Alley, E. S., Yaméogo, L., Dadzie, K. Y. & Boatin, B. A. Eliminating onchocerciasis after 14 years of vector control: a proved strategy. *J. Infect. Dis.* **184**, 497–503 (2001).

45. World Health Organization & African Programme for Onchocerciasis Control. Progress report of the special intervention zones of the ex-OCP, January-August 2006. Dar-es-Salaam, Tanzania 5-8 December 2006. https://iris.who.int/handle/10665/275951 (2006).

46. Boatin, B., Molyneux, D. H., Hougard, J. M., Christensen, O. W., Alley, E. S., Yaméogo, L., Sékétéli, A. & Dadzie, K. Y. Patterns of epidemiology and control of onchocerciasis in west Africa. *J. Helminthol*. **71**, 91–101 (1997).

47. Krentel, A., Fischer, P. U. & Weil, G. J. A review of factors that influence individual compliance with mass drug administration for elimination of lymphatic filariasis. *PLoS Negl. Trop. Dis.* **7**, e2447 (2013).

48. Senyonjo, L., Oye, J., Bakajika, D., Biholong, B., Tekle, A., Boakye, D., Schmidt, E. & Elhassan, E. Factors associated with ivermectin non-compliance and its potential role in sustaining *Onchocerca volvulus* transmission in the West Region of Cameroon. *PLoS Negl. Trop. Dis.* **10**, e0004905 (2016).

49. Turner, H. C., Churcher, T. S., Walker, M., Osei-Atweneboana, M. Y., Prichard, R. K. & Basáñez, M.-G. Uncertainty surrounding projections of the long-term impact of ivermectin treatment on human onchocerciasis. *PLoS Negl. Trop. Dis.* **7**, e2169 (2013).

50. World Health Organization & African Programme for Onchocerciasis Control. Report of the fifth activity review and planning meeting of the Special Intervention Zones (SIZ). Ouagadougou 8-10 November 2006. https://iris.who.int/handle/10665/276197 (2006).

51. World Health Organization. Guidelines for stopping mass drug administration and verifying elimination of human onchocerciasis: criteria and procedures. 2016. https://www.who.int/publications/i/item/9789241510011 (2016).

52. Amaral, J.-J. & Basáñez, M.-G. GitHub repository: mrc-ide/EPIONCHO.IBM. https://doi.org/10.5281/zenodo.17351356 (2025).

53. Katholi, C. R., Toé, L., Merriweather, A. & Unnasch, T. R. Determining the prevalence of *Onchocerca volvulus* infection in vector populations by polymerase chain reaction screening of pools of black flies. *J. Infect. Dis.* **172**, 1414–1447 (1995).

54. Post, R. J., Cheke, R. A., Boakye, D. A., Wilson, M. D., Osei-Atweneboana, M. Y., Tetteh-Kumah, A., Lamberton, P. H. L., Crainey, J. L., Yaméogo, L. & Basáñez, M.-G. Stability and change in the distribution of cytospecies of the *Simulium damnosum* complex (Diptera: Simuliidae) in southern Ghana from 1971 to 2011. *Parasit. Vectors* **6**, 205 (2013).

55. Lamberton, P. H. L., Cheke, R. A., Walker, M., Winskill, P., Osei-Atweneboana, M. Y., Tirados, I., Tetteh-Kumah, A., Boakye, D. A., Wilson, M. D., Post, R. J. & Basáñez, M.-G. Onchocerciasis transmission in Ghana: biting and parous rates of host-seeking sibling species of the *Simulium damnosum* complex. *Parasit. Vectors* **7**, 511 (2014).

56. World Health Organization & Onchocerciasis Control Programme in West Africa. Progress Report of the World Health Organization for 1998 (1 September 1997-31 August 1998). Joint Programme Committee 19th Session. Accra 7-9 December 1998. https://iris.who.int/bitstream/handle/10665/311230/JPC19.2-eng.pdf?sequence=1 (1998).

57. Organisation Mondiale de la Santé, Programme Africain de Lutte contre l'Onchocercose & Badila, C. Initiation aux techniques de lutte contre l'onchocercose: application des techniques d'épandage de larvicides, d'évaluation entomologique, des techniques d'évaluation épidémiologique et de traitement à l'ivermectine. Rapport de stage dans les Zones d'Interventions Spéciales (SIZ) de lutte contre l'onchocercose au Togo et au Benin, 4 août-14 décembre 2003. https://iris.who.int/handle/10665/367599 (2003).

58. Turner, H. C., Kura, K., Roth, B., Kuesel, A. C., Kinrade, S. & Basáñez, M.-G. An updated economic assessment of moxidectin treatment strategies for onchocerciasis elimination. *Clin. Infect. Dis.* **78(Suppl 2)**, S138–S145 (2024).

59. Jacob, B. G., Loum, D., Lakwo, T. L., Katholi, C. R., Habomugisha, P., Byamukama, E., Tukahebwa, E., Cupp, E. W. & Unnasch, T. R. Community-directed vector control to supplement mass drug distribution for onchocerciasis elimination in the Madi mid-North focus of Northern Uganda. *PLoS Negl. Trop. Dis*. **12**, e0006702 (2018).

60. Lakwo, T. L., Siewe Fodjo, J., Jada, S. R., Alinda, P., Tionga, M., Marcello, C. D. R., War, D. G. D., Saleeb, M. & Colebunders R. A community-based vector control intervention "Slash and Clear" implemented in two onchocerciasis-endemic foci in South Sudan. *PLoS Negl. Trop. Dis*. **19**, e0013309 (2025).

61. Behrend, M. R., Basáñez, M.-G., Hamley, J. I. D., Porco, T. C., Stolk, W. A., Walker, M., de Vlas, S. J. & NTD Modelling Consortium. Modelling for policy: the five principles of the Neglected Tropical Diseases Modelling Consortium. *PLoS Negl. Trop. Dis.* **14**, e0008033 (2020).
