# Supplementary material for: Reaching elimination of onchocerciasis transmission with long-term vector control and ivermectin treatment in Togo
Source: Nat Commun. 2025 Dec 19;17:779. doi: 10.1038/s41467-025-67451-8 (PMC12823694; doi:10.1038/s41467-025-67451-8)
Supplement: Supplementary file 2 — Reporting Summary [file 41467_2025_67451_MOESM2_ESM.pdf]

nature portfolio

Corresponding author(s): Maria-Gloria Basáñez and Luis-Jorge Amaral

Last updated by author(s): Oct 24, 2025

Reporting Summary

Nature Portfolio wishes to improve the reproducibility of the work that we publish. This form provides structure for consistency and transparency in reporting. For further information on Nature Portfolio policies, see our Editorial Policies and the Editorial Policy Checklist.

Statistics

For all statistical analyses, confirm that the following items are present in the figure legend, table legend, main text, or Methods section.

n/a	Confirmed	
☐	☒	The exact sample size (*n*) for each experimental group/condition, given as a discrete number and unit of measurement
☐	☒	A statement on whether measurements were taken from distinct samples or whether the same sample was measured repeatedly
☒	☐	The statistical test(s) used AND whether they are one- or two-sided<br>*Only common tests should be described solely by name; describe more complex techniques in the Methods section.*
☒	☐	A description of all covariates tested
☒	☐	A description of any assumptions or corrections, such as tests of normality and adjustment for multiple comparisons
☐	☒	A full description of the statistical parameters including central tendency (e.g. means) or other basic estimates (e.g. regression coefficient) AND variation (e.g. standard deviation) or associated estimates of uncertainty (e.g. confidence intervals)
☐	☒	For null hypothesis testing, the test statistic (e.g. *F*, *t*, *r*) with confidence intervals, effect sizes, degrees of freedom and *P* value noted<br>*Give P values as exact values whenever suitable.*
☒	☐	For Bayesian analysis, information on the choice of priors and Markov chain Monte Carlo settings
☒	☐	For hierarchical and complex designs, identification of the appropriate level for tests and full reporting of outcomes
☒	☐	Estimates of effect sizes (e.g. Cohen's *d*, Pearson's *r*), indicating how they were calculated

Our web collection on statistics for biologists contains articles on many of the points above.

Software and code

Policy information about availability of computer code

Data collection	No software was used to collect the data. This is a transmission dynamics modelling analysis of data already collected and made publicly available.
Data analysis	Data analysis (modelling of infection trends) was performed using the individual-based, stochastic transmission model EPIONCHO-IBM, published by Hamley et al. (2019) in PLoS Negl Trop Dis 13: e0007557. The model code and full documentation is hosted and maintained at: https://github.com/mrc-ide/EPIONCHO.IBM. The specific model code and scripts for figures is given in Amaral, J.-J. & Basáñez, M.-G. GitHub repository: mrc-ide/EPIONCHO.IBM. https://doi.org/10.5281/zenodo.17351356 (2025). Analyses were conducted in R (v4.4.1). Core packages: dplyr (v1.1.4), tibble (v3.2.1), ggplot2 (v4.0.0), patchwork (v1.3.0) for figure assembly; spatial data handled with sf (v1.0-21), terra (v1.8-50), and geodata (v0.6-2); plot utilities included ggrepel (v0.9.6), ggnewscale (v0.5.1), ggforce (v0.4.2), and scatterpie (v0.2.4).

For manuscripts utilizing custom algorithms or software that are central to the research but not yet described in published literature, software must be made available to editors and reviewers. We strongly encourage code deposition in a community repository (e.g. GitHub). See the Nature Portfolio guidelines for submitting code & software for further information.

Data

Policy information about availability of data

All manuscripts must include a data availability statement. This statement should provide the following information, where applicable:

- Accession codes, unique identifiers, or web links for publicly available datasets
- A description of any restrictions on data availability
- For clinical datasets or third party data, please ensure that the statement adheres to our policy

The database containing the epidemiological data has been made publicly available by Vinkeles Melchers et al. (2024) in PLoS Negl Trop Dis 18: e0012312 at: https://doi.org/10.1371/journal.pntd.0012312.s001. All information used for the analyses is contained in this database, and in the figures, tables, and Supplementary Material files of the paper submitted to Nature Communications.

Research involving human participants, their data, or biological material

Policy information about studies with human participants or human data. See also policy information about sex, gender (identity/presentation), and sexual orientation and race, ethnicity and racism.

Reporting on sex and gender	The analysis presented is based on modelling of the infection trends of village-level prevalence data, categorised by region endemicity level, and Special Intervention Zone (SIZ) status in Togo and, therefore, not disaggregated by sex or gender.
Reporting on race, ethnicity, or other socially relevant groupings	We do not report on race, ethnicity or other socially-relevant groups. Onchocerciasis-endemic villages are mostly rural populations. In this paper, we focus on Togo, in West Africa.
Population characteristics	The data come from parasitological surveys (using the skin-snip microscopy diagnostic method) conducted at village level in participants of all ages. The treatment of onchocerciasis in endemic populations is through mass drug administration (MDA) of ivermectin in the population aged 5 years and older. In Togo, annual (yearly) and biannual (6-monthly) MDA has been implemented.
Recruitment	The epidemiological surveys that we model were conducted by the Onchocerciasis Control Programme in West Africa (OCP) from its inception in 1975 and, at the closure of the programme in 2002, by the National Onchocerciasis Control Programme of the Ministry of Health of Togo. The proportion of the population examined by skin-snip microscopy ranged from 70% to 80% of the total population in the village. There was a slight decrease of the proportion of the population examined over time, and we have reported this in the Supporting Information File 1. We also report 95% confidence intervals for each village to provide a measure of uncertainty in the infection prevalence estimates.
Ethics oversight	When the surveys were conducted by the OCP (1975-2002), the participating countries (in our case, Togo) signed a Memorandum of Agreement that covered all issues pertaining to the operations and covered clearance for the parasitological surveys. Therefore, the surveys satisfied the requirements for ethical clearance within the Memorandum. Additionally, a committee consisting of Chief of Units of the OCP ensured that the plans and methodology of work were correctly followed by the technicians in the field. Communities were free to participate in the taking of skin snip samples. The same principles applied to the data collected by the Ministry of Health of Togo. The authors of this manuscript did not collect primary data, and therefore no ethical approval applies. Among the co-authors of the paper are staff of the National Onchocerciasis Control Programme of Togo.

Note that full information on the approval of the study protocol must also be provided in the manuscript.

Field-specific reporting

Please select the one below that is the best fit for your research. If you are not sure, read the appropriate sections before making your selection.

☒ Life sciences ☐ Behavioural & social sciences ☐ Ecological, evolutionary & environmental sciences

For a reference copy of the document with all sections, see nature.com/documents/nr-reporting-summary-flat.pdf

Life sciences study design

All studies must disclose on these points even when the disclosure is negative.

Sample size	Sample sizes were not predetermined, as the data we model originate from village-level prevalence surveys in which 70% to 80% of the population participated, as described above. Our aim was to model the infection trends over time as a result of the onchocerciasis interventions implemented in Togo, i.e., vector control and ivermectin treatment. By way of providing uncertainty bounds to our model projections, we model minimal, reference and enhanced intervention scenarios, that serve to provide lower, average, and upper simulated epidemiological trends. We also plot 95% confidence intervals for the villages included in each endemicity level according to their baseline (pre-control) prevalence estimates. We also provide 95% confidence intervals for all surveys conducted over time.
Data exclusions	No data were excluded. We used a total of 400 villages.
Replication	This is not an experimental study. Reproducibility will be based on other researchers being able to reproduce our results by using the data and model code we provide.

Randomization	This is not an experimental study. Participants were free to take part in the parasitological surveys. Of the total population in the villages, 70% to 80% of the census population agreed to be examined. Therefore, we consider that the prevalence estimates obtained were representative of the epidemiological situation as it evolved over time under the interventions implemented.
Blinding	This is not applicable. All eligible individuals in the communities were offered treatment with ivermectin.

Reporting for specific materials, systems and methods

We require information from authors about some types of materials, experimental systems and methods used in many studies. Here, indicate whether each material, system or method listed is relevant to your study. If you are not sure if a list item applies to your research, read the appropriate section before selecting a response.

Materials & experimental systems

n/a	Involved in the study
☒	☐ Antibodies
☒	☐ Eukaryotic cell lines
☒	☐ Palaeontology and archaeology
☒	☐ Animals and other organisms
☒	☐ Clinical data
☒	☐ Dual use research of concern
☒	☐ Plants

Methods

n/a	Involved in the study
☒	☐ ChIP-seq
☒	☐ Flow cytometry
☒	☐ MRI-based neuroimaging

Plants

Seed stocks	Not applicable.
Novel plant genotypes	Not applicable.
Authentication	Not applicable.
